# Vision transformer autoencoders captures local and non-local features in brain imaging to reveal novel genetic associations

**DOI:** 10.1038/s42003-026-10430-6

**Published:** 2026-06-09

**Authors:** Samia R. Islam, Tian Xia, Wei He, Ziqian Xie, Degui Zhi

**Affiliations:** https://ror.org/03gds6c39 grid.267308.8 0000 0000 9206 2401 D. Bradley McWilliams School of Biomedical Informatics, The University of Texas Health Science Center at Houston, Houston, USA

**Keywords:** Computational models, Machine learning, Image processing

## Abstract

Linking genetic variation to human brain structure is a key step toward understanding the biological basis of cognition and disease. Progress in this area, however, has been limited by a major challenge: imaging features are often predefined, restricting the discovery of novel associations. Here, we present a framework that applies a Vision Transformer (ViT)-based autoencoder to derive 128-dimensional representations from T1-weighted brain MRI scans of 6,130 UK Biobank participants, which we call unsupervised learning derived image phenotypes from ViT (ViT-UDIP). These ViT-UDIP phenotypes are used in genome-wide association studies (GWAS) of 22,867 UK Biobank participants to identify significant genetic variants, which were further aggregated into genetic loci. The ViT-based approach uncovers a total of 63 loci and out of which 24 were not detected by the CNN-based method. Importantly, feature interpretation reveals that the model captured local as well as non-local anatomical patterns such as left-right hemisphere symmetry within brain MRI data by leveraging its attention mechanism and positional embeddings. This ability of capturing non-local patterns distinguishes the ViT from the previous CNN model. Together, these results demonstrate the value of transformer-based architectures in discovering novel and robust imaging phenotypes for genetic discovery.

**Figure:**
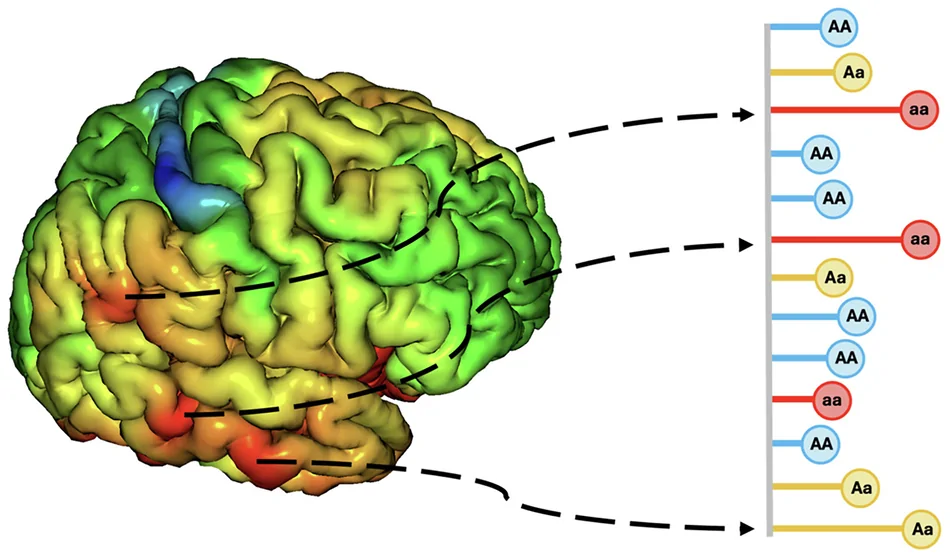

## Abstract (web summary)

A computational framework applies a Vision Transformer (ViT)-based autoencoder to generate unsupervised learning-derived image phenotypes (ViT-UDIPs) based on T1-weighted brain MRI scans from a subset of UK Biobank participants. These ViT-UDIPs were used in a GWAS across a separate pool of UK Biobank participants to identify 63 loci, including 24 loci not detected by CNN-based methods.

## Introduction

Insights into understanding brain structural anatomy and related genetic signals have the potential to pave the way for breakthroughs in neuroscience and precision medicine. Brain structure genome-wide association studies (GWAS) have employed a range of phenotypic representations. One line of work uses direct morphometric measurements or low-dimensional curated features^1–5^ or statistical and mathematical representations of anatomical modeling and population genetics^6,7^. A parallel line has shifted toward high-throughput image-derived phenotypes (IDPs) enabled by large-scale imaging cohorts and standardized processing pipelines. These IDPs features higher-dimensional or data-driven representations of brain structure^8–11^. While these approaches have led to interesting discoveries, they rely on prior knowledge and constraints and may overlook more nuanced or complex patterns in the data.

Recent advances in machine learning techniques offer more flexible and data-driven methods that can learn important features from raw imaging data like T1-weighted magnetic resonance imaging (MRI) brain scans. These methods can capture fine-grained structural information and generate unbiased representations of the underlying anatomy^12^. Some studies have adopted supervised deep learning for extracting image features, taking advantage of Alzheimer’s Disease Neuroimaging Initiative (ADNI) data^13^. One step further, Alzclip leverages supervised learning with labels from ADNI and applies contrastive learning to align genetic and imaging features within a shared latent space, enabling the study of relationships between genetic risk factors and neuroimaging markers^14^. Other studies have similarly explored the integration of genetic and imaging data for disease prediction, facilitating the derivation of informative image features^15–19^. However, a key limitation of these approaches to derive deep image features is the reliance on the label of the datasets, which are often scarce and may introduce biases inherited from human annotation.

On the other hand, unsupervised deep learning has been applied to extract image features without requiring labeled data. Patel et al^20^. used Convolutional Neural Network (CNN)-based unsupervised deep learning to discover phenotypes for GWAS of brain imaging because this approach can bypass the challenges of relying on predefined features, segmentation, and manual annotation by enabling models to learn complex and relevant features directly from the raw image data, where the model creates an *n*-dimensional representation known as Unsupervised Deep learning derived Imaging Phenotypes (UDIPs). Similarly, Dufournet et al utilized a self-supervised learning framework to uncover cortical folding patterns influenced by genetic variation^21^. In another line of work, Rakowski et al applied transfer learning by leveraging models pretrained on ADNI or ImageNet to UK Biobank imaging data, deriving low-dimensional representations suitable for GWAS analyses^22^. Another interesting study is by Yu et al., who used Single Nucleotide Polymorphism (SNPs) as a supervision signal to train CNN models on 2D MRI images, ultimately selecting the top SNPs based on their prediction performance^23^. This is essentially a novel approach to conducting non-linear GWAS using a deep learning architecture, with the sacrifice of interpretablity and the cost of train thousands of CNN models.

Here, we propose an approach where an unsupervised learning vision transformer (ViT) model^24^ is used for brain imaging GWAS. This utilizes a different inductive bias from CNN architectures, which typically build representations through stacked layers of template matching, whereas the ViT uses patch-based self-attention to directly model potentially long-range interactions within the image^25^. ViTs have demonstrated strong performance in various medical imaging applications, including feature extraction from brain MRI ^26^, their architectural design facilitating the direct modeling of global context and long-range dependencies, even in the early layers^26,27^.

This paper presents a novel Vision Transformer autoencoder to derive a new class of phenotypes, ViT-UDIPs, directly from brain MRI data. A genome-wide association study performed on these ViT-UDIPs reveals novel genetic loci that were not captured by previous CNN analyses. Additionally, an interpretability framework is adopted to probe the model’s learned pattern, offering insights into the ViT-derived phenotypes. Using the perturbation-based decoder interpretation (PerDI) method, we uncovered how ViT uniquely and effectively captures non-local patterns and extracts hidden features from the brain in a comprehensive manner—something that CNN-based architectures could not achieve. Together, this manuscript introduces an unbiased, and insightful approach to deriving features from the brain. It also reports many novel genes and GWAS categories that warrant further investigation by biologists to validate their potential links with brain-related genetic mechanisms.

## Results

### ViT-based autoencoder for extracting imaging phenotypes (UDIPs) for genetic discovery

Our overall pipeline is shown in Fig. 1. The four major steps are data selection, ViT-Autoencoder (ViT-AE) model development, GWAS, and finally, ViT-UDIP to brain mapping via the PerDI approach. Details of the steps can be found in the Methods section.

**Fig. 1:**
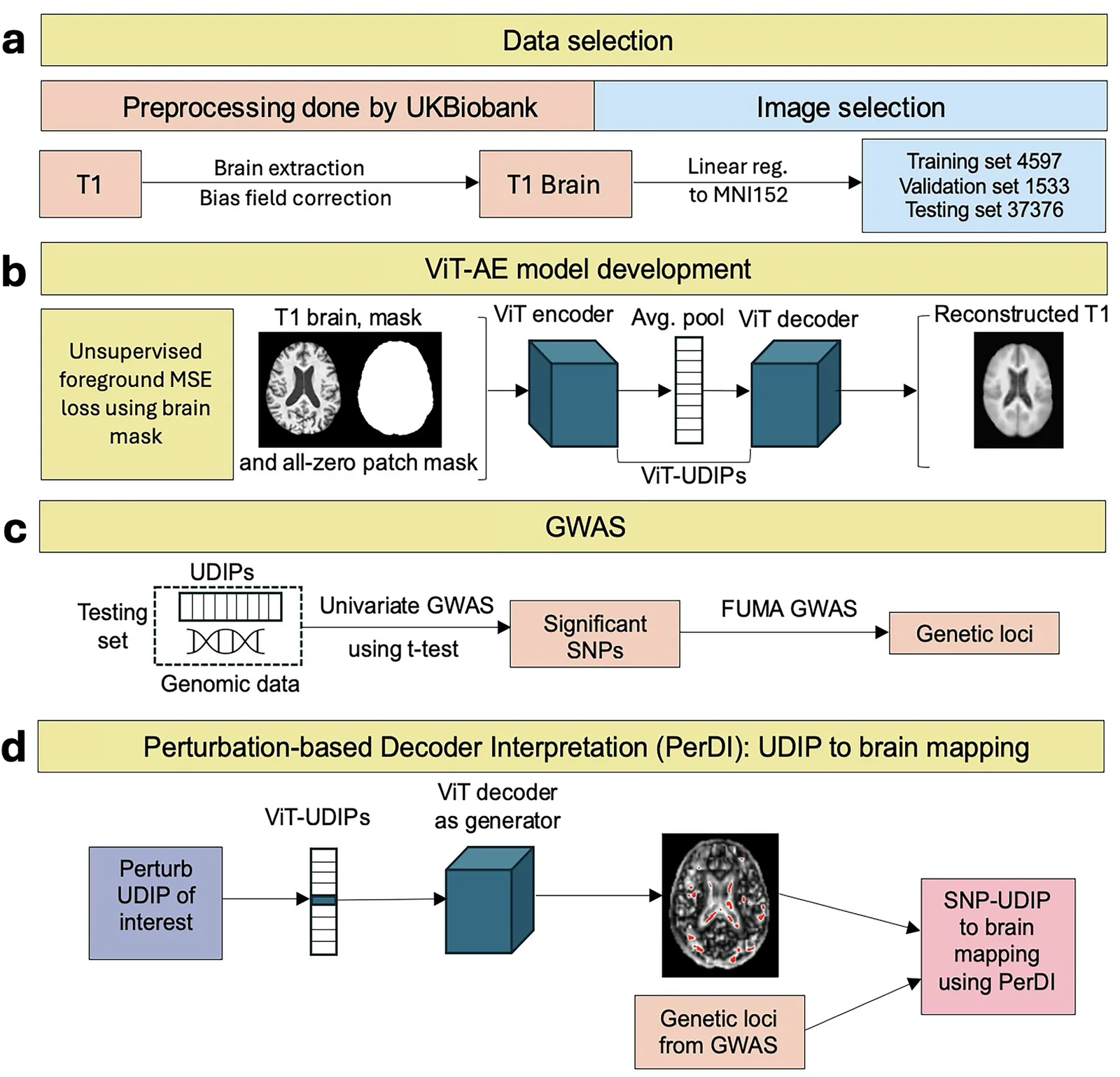
Overall pipeline of the study. **a** T1 brain MRI preprocessed by UKB were used for training, validation and testing. **b** ViT-AE trained by background masked mean square error (MSE) loss of non-zero patches. **c** Genetic loci discovered by univariate GWAS and FUMA GWAS. **d** SNP-ViT-UDIP to brain mapping performed using Perturbation-based Decoder Interpretation (PerDI). Images reproduced by kind permission of UK Biobank ©.

### Optimization based on the total loci discovery and loci overlapping with previous IDP

The ViT-AE model was trained to reconstruct the input using background masked mean square error (see Methods: ViT-AE model). The average validation loss of the ViT-AE model remained at approximately 0.22 after 200 epochs, as shown in Fig. 2a. The training stop point is set at epoch 300, when we did not see any decrease in validation loss and improvement in the number of discovered loci.

**Fig. 2:**
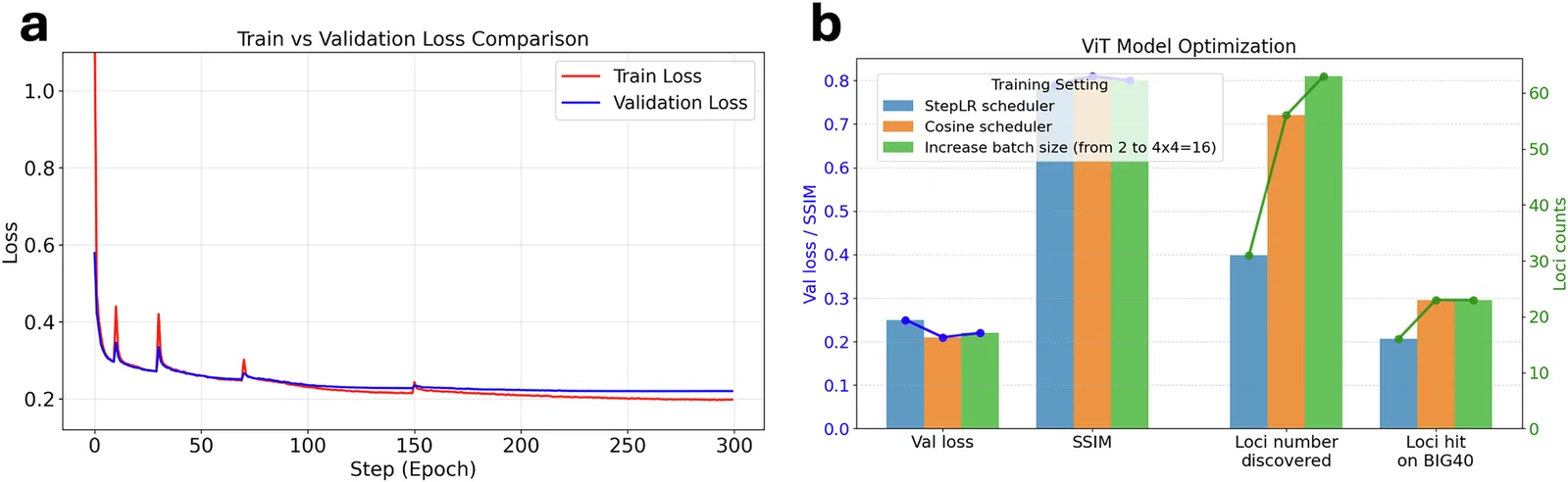
ViT-AE model optimization. **a** Training and validation loss of ViT-AE (train *n* = 4597, valiation *n* = 1533). **b** Comparison of various training configurations in terms of their effects on both model performance and genetic analysis outcomes.

We introduce two metrics to assess the model’s performance: the total number of loci discovered and the number of overlapping loci with those previously reported. Specifically, we used the BIG40 database—a comprehensive GWAS study conducted on approximately 40,000 UK Biobank participants (Smith et al. ^9^) which analyzed 3,929 IDPs (https://open.win.ox.ac.uk/ukbiobank/big40/). We performed both training and architectural optimizations for the ViT-AE model majorly using the reconstruction metric (Validation loss Mean Squared Error (MSE) and Structural Similiarity Index Measure (SSIM)). For training, the optimal configuration was found to be the AdamW optimizer combined with a cosine learning rate scheduler and a batch size of 16. This setup yielded the highest number of discovered loci (63) and the greatest overlap with loci from the BIG40 panel (Fig. 2b). This configuration is adopted as the current setting, and all subsequent results are based on this setup. Regarding architectural improvements, we explored multiple strategies (see Supplementary Note 1, Supplementary Data 1, 2), including patch embedding method, pooling mechanisms to generate the UDIPs, and loss functions. The best performing model was found to be the one that uses average pooling and anisotropic position embedding that emphasizes short axis, which is the configuration in the current model.

As a comparison, the CNN-based UDIP model^20^ achieved an average validation loss of approximately 0.28 after 15 epochs that increased to 0.3 at 25 epochs (see Supplementary Fig 1). Visual observation of reconstruction was similar to that of the ViT-AE model (see Supplementary Fig 2).

To demonstrate the reproducibility of these UDIPs, we trained the network under three sources of variability: different training seeds, different data splits, and a distinct dataset (ADNI). The results show strong alignment in UDIP distributions, as measured by centered kernel analysis (CKA) and a high degree of overlap in the identified loci. Specifically, CKA (range 0 to 1) achieves an average of 0.83 across different training seeds, 0.80 across different data splits, and 0.60 across distinct datasets. Moreover, the explained variance (see Methods) of one UDIP by another averages around 0.7. The overlap analysis shows an average of 37 loci for different training seeds, 35 for different data splits, and 17 for distinct datasets. Additional details are provided in Supplementary Note 2, Supplementary Fig 3-6, and our latest analyses^28^.

### Novel loci discovered associated with brain structure

GWAS was performed on the test dataset UDIPs from the best checkpoint at epoch 300 with the lowest validation loss. We applied the mixed linear model fastGWA to compute p-values for each SNP-ViT-UDIP pair and used FUMA to clump significant SNPs (those with the minimum p-value passing the Bonferroni-corrected threshold) into genomic loci.

Here, we present the FUMA-based GWAS results from the model checkpoint at epoch 300. In total, 63 genomic loci were identified (see Supplementary Datas 3 and 4 for all loci and associated lead SNPs and genes^29^, among which 24 loci were not previously reported by the CNN-based UDIP approach, which we define as novel loci^20^. Addtionally, 20 loci were not previously reported by either the CNN-based UDIP approach or IDP approach^8^ (Supplementary Fig 7 and Supplementary Data 5). The lead SNPs and their nearest genes are highlighted in Fig. 3. Notably, four of the newly identified loci are located on chromosome 2, and three on chromosomes 4, 6, and 14, respectively. As an example, the *SLC39A8* gene on chromosome 4 was found to be significantly associated with the ViT-UDIP feature at dimension 95, suggesting a potential link between the genetic activity of *SLC39A8* and the representation captured in this embedding dimension.

**Fig. 3:**
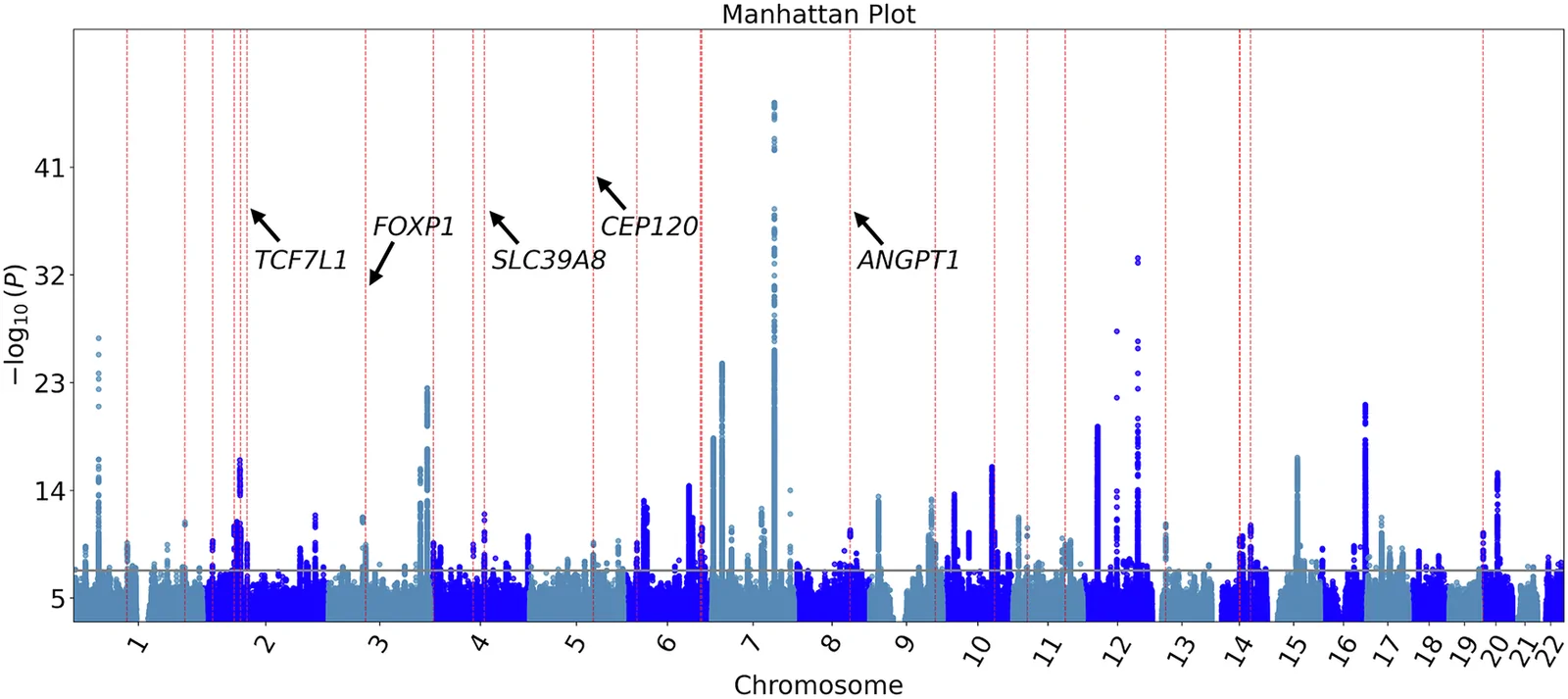
New loci discovered from the ViT-based UDIP approach. The red line indicates the lead SNP within novel loci that were not previously identified by the CNN-based UDIP. The nearest gene previously known to be associated with brain structure and function is highlighted in the graph. A Miami plot compares the genetic discovery between ViT and CNN UDIP can be found in the Supplementary Fig 13.

Table 1 provides a detailed summary of the 24 loci that were not previously reported in the CNN-based UDIP approach. The table lists the locus ID, the corresponding chromosome, the lead SNP (i.e., the variant with the lowest p-value within each locus), the specific dimension contributing to the lowest p-value, and the nearest gene to the lead SNP. Notably, several loci have been previously reported to be associated with multiple GWAScatalog traits from FUMA. The complete table is provided in Supplementary Data 6.

**Table 1:** New discovered loci

Loci ID	Chr	Lead SNP	ViT dim	MAF	BETA	P	Nearest gene
2	1	rs9787064	31	0.415164	0.010316	2.45E-10	*RP5-936J12.1*
4	1	rs6540873	70	0.379592	0.00853342	4.59E-12	*RP11-323K10.1*
6	2	rs2346965	77	0.150558	−0.0197863	1.62E-10	*AC092594.1*
7	2	rs13009393	88	0.413358	−0.0066133	9.72E-12	*AC007381.2*
10	2	rs11126381	20	0.30422	0.00885714	1.61E-11	*EXOC6B*
11	2	rs34644194	50	0.394577	0.010794	2.93E-10	*TCF7L1*
14	3	rs62244889	127	0.427973	−0.0068934	3.43E-10	*FOXP1*
17	4	rs4689932	108	0.442456	−0.0062159	2.43E-10	*STX18-AS1*
18	4	rs112867328	106	0.0178811	0.0330002	3.38E-10	*C4orf22*
19	4	rs13107325	95	0.0704971	−0.0135442	1.00E-12	*SLC39A8*
21	5	rs13156484	44	0.472685	0.00689785	2.33E-10	*CEP120*
23	6	rs7766641	98	0.261631	−0.0079467	2.40E-10	*HIST1H2BE*
28	6	rs2486411	56	0.304494	0.0137253	5.40E-11	*UST*
29	6	rs4869739	50	0.288615	0.0122517	1.30E-11	*CCDC170*
37	8	rs1283690	116	0.417223	0.00724871	2.04E-11	*ANGPT1*
41	9	rs10760293	42	0.366411	0.00751107	2.98E-10	*DENND1A*
45	10	rs10786393	95	0.429466	0.00646394	2.91E-11	*R3HCC1L*
47	11	rs7938467	50	0.454874	0.0111945	1.41E-11	*RP11-159H22.2*
49	11	rs61890632	49	0.166249	−0.0092627	3.27E-10	*PGR*
54	13	rs306395	46	0.434194	0.00553429	6.36E-12	*ATP8A2:AL138815.1*
55	14	rs1959830	91	0.302913	−0.0084635	1.04E-10	*AL163953.3*
56	14	rs4444235	20	0.46131	−0.0078367	9.98E-11	*MIR5580*
58	14	rs7147721	91	0.456405	0.00828745	8.23E-12	*FCF1*
62	20	rs6085574	61	0.278401	0.00739805	3.63E-11	*CASC20*

Out of the 24 loci newly identified by our method, 14 loci show at least one of their top five GWAScatalog traits from FUMA related to brain structure or function. The most frequent GWAScatalog traits include whole-brain restricted isotropic diffusion, whole-brain free water diffusion, whole-brain restricted directional diffusion^30^, and regional brain volumes. That study employed a multivariate GWAS approach, which tends to yield lower p-values compared with univariate analyses, explaining why these GWAScatalog traits often appear among the top associations. Nevertheless, the overlap with diffusion MRI–based GWAScatalog traits supports that our UDIP framework successfully identifies loci linked to subtle functional variations in brain structure.

In contrast, of the 24 newly identified loci, 10 are such that none of their top five reported traits in the GWAScatalog are related to brain structure or function (Supplementary Data 6, marked in red). The most commonly reported non-brain GWAScatalog traits include bone mineral density, height, body mass index (BMI), body fat distribution, and cardiovascular-related GWAScatalog traits. Although these loci are not previously associated with brain-related traits, many map to genes involved in fundamental developmental pathways, such as Wnt and TGF-β signaling. These pathways regulate cell proliferation, differentiation, and tissue patterning across multiple organ systems. As a result, associations with traits such as bone mineral density, height, and body composition may reflect shared biological mechanisms governing overall morphology, including brain structure. In short, it opens exciting opportunities for researchers to investigate how GWAScatalog traits such as bone mineral density and lung or colorectal dysfunction can be linked to the brain through the loci or genes described in the table below.

Multiple genes in Table 1 contribute to the intricate processes underlying brain development, neuronal differentiation, and neurovascular homeostasis, among which *FOXP1*, *TCF7L1*, *SLC39A8*, *CEP120*, and *ANGPT1* have been implicated in both structural and functional aspects of neural biology. These genes represent diverse but convergent mechanisms, transcriptional control (*FOXP1, TCF7L1*), ion transport (*SLC39A8*), centrosomal regulation (*CEP120*), and vascular signaling (*ANGPT1*), that coordinate brain formation and function (see Supplementary Note 3). Their associations with neurodevelopmental disorders highlight that perturbations in distinct cellular systems, from transcriptional to metabolic and structural, ultimately converge on shared pathways governing brain integrity and cognition.

Using LD score regression (LDSC), we found that ViT-UDIPs achieved a higher mean heritability (0.219; Supplementary Fig 8) than UK Biobank T1-based IDPs (0.176), with heritability estimates comparable to those of CNN-UDIPs.

### Visualizing endophenotype activation patterns by PerDI

To begin, we validate the model’s training effectiveness by examining the reconstructed images of two individuals who show clear anatomical differences. The reconstructions produced by the ViT-AE model successfully preserve these structural distinctions (Fig. 4a). We further evaluated the model on the test set and inspected the reconstruction loss to detect any out-of-distribution samples (see Supplementary Note 4).

**Fig. 4:**
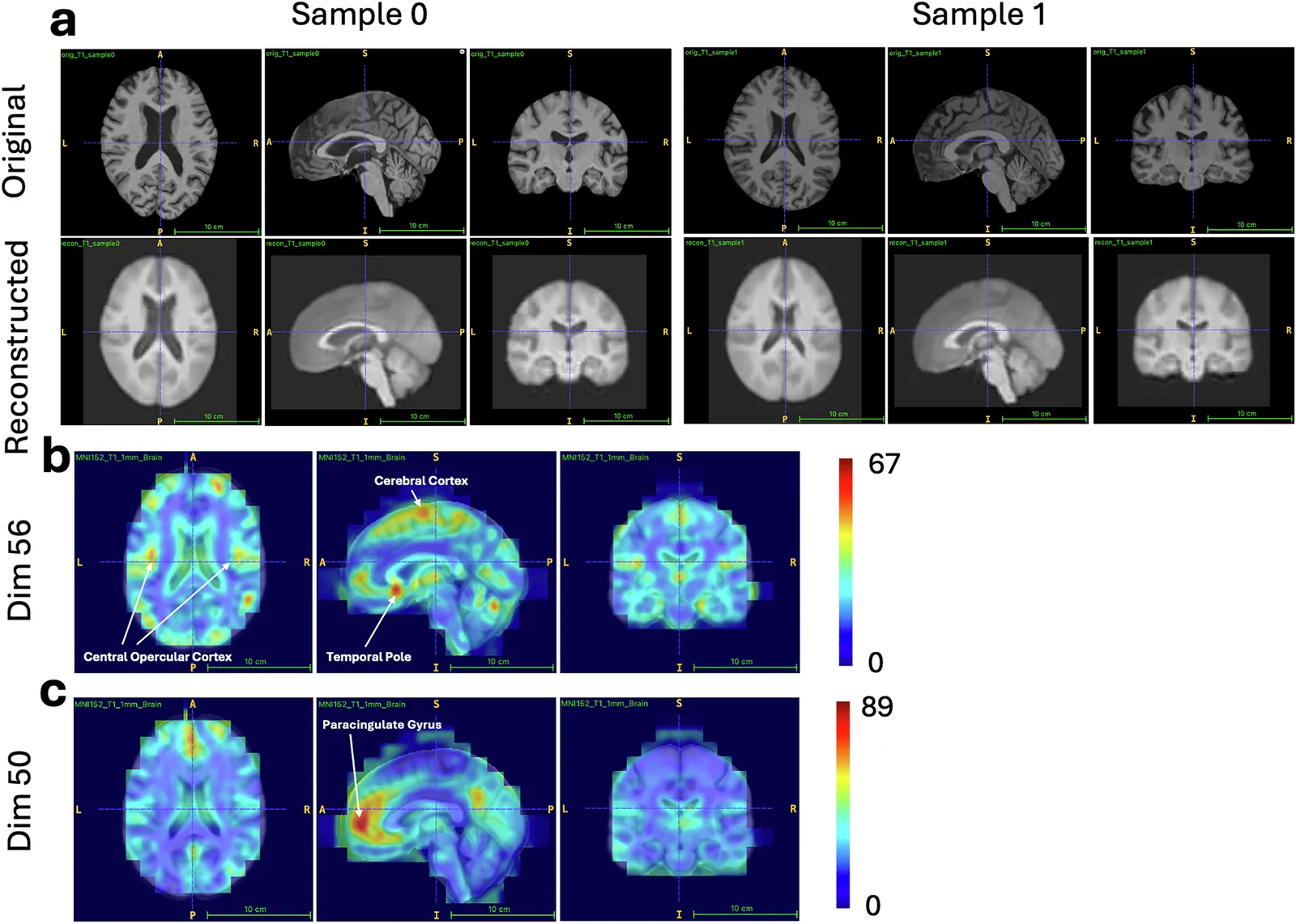
Interpretation of ViT-UDIP. **a** Validation of the model not collapsing. **b** Activation regions for dimensions 56 show the model’s ability to represent the brain in an integrated manner. The color bar represents the t-statistic values, ranging from blue to red, where red indicates higher t-statistics. **c** Activation regions for dimensions 50 show the model’s focus on a certain region of the brain. The color bar represents the t-statistic values, ranging from blue to red, where red indicates higher t-statistics. Images reproduced by kind permission of UK Biobank ©.

We then performed PerDI analyses across all dimensions. Briefly, for 500 test subjects, we introduced a perturbation by adding one standard deviation of noise to the 128 ViT-UDIPs. The decoder then reconstructed images from these perturbed ViT-UDIPs, and a voxel-wise paired t-test was performed between the original and perturbed reconstructed images. Using ITK-SNAP, we visualized PerDI *t*-maps for two representative dimensions (Fig. 4b, c). Dimension 56 was selected because it yielded the highest number of lead SNPs with the lowest p-values. Dimension 50 was chosen as it identified the greatest number of novel lead SNPs with low p-values that had not been previously reported by CNN. Activated regions were identified by overlaying these maps with the Harvard–Oxford Cortical and Subcortical Atlases, as well as the MNI Structural Atlas.

We found that, in certain dimensions, the PerDIs capture a whole-brain representation. Figure 4b illustrates the activation regions for dimension 56, which contribute to most loci. The Central Opercular Cortex is highlighted on both hemispheres, along with the Cerebral Cortex. Aside from the emphasis on the Temporal Pole, the overall activation aligns closely with cortical regions and grey matter. Across other dimensions, similar patterns were observed, consistently highlighting brain anatomical structures and demonstrating the model’s ability to represent the brain in a comprehensive and integrated manner.

Furthermore, certain dimensions exhibited a more distinct focus. In particular, dimension 50, which contributes most to previously undiscovered loci and ranks among the top three contributors overall, shows a notable focus on the Paracingulate Gyrus (Fig. 4c). The Paracingulate Gyrus, located in the medial frontal cortex just above the cingulate gyrus, is implicated in higher-level cognitive functions such as self-reflection and self-awareness, theory of mind (understanding others’ mental states), decision-making and conflict monitoring, and emotional regulation, often overlapping with anterior cingulate cortex functions^31^. Interestingly, the three lead SNPs associated with dimension 50 have all been previously linked to bone mineral density (BMD; see Supplementary Data 6). While no direct evidence connects BMD specifically to the Paracingulate Gyrus, a study in the UK Biobank indicates that low BMD is associated with decreased gray matter volume^32^, suggesting that BMD could indirectly influence regions like the Paracingulate Gyrus via gray matter changes. There is also a possibility of horizontal pleiotropy, in which a single genetic variant independently affects multiple distinct traits, in this case, affecting both BMD and gray matter volume.

The PerDI ViT-UDIP brain mapping demonstrated the ViT-AE model’s ability to capture activations across the brain, revealing structural patterns at varying levels of detail. These activations reflected the model’s capacity to identify symmetrical patterns across bilateral structures, consistent with the brain’s inherent anatomical symmetry, and to recognize fine-grained structural details.

### Sparse attention patterns facilitate novel genetic discovery

We investigated the attention scores of the trained model and found some interesting patterns. From the very first attention layer, the model started to focus on a subset of patches across different heads. The attention pattern of each head was extremely sparse (Fig. 5a), while different heads at different layers focused on different patches. This subset of patches consisted of less than one-fifth of the total patches, with all other patches attending them.

**Fig. 5:**
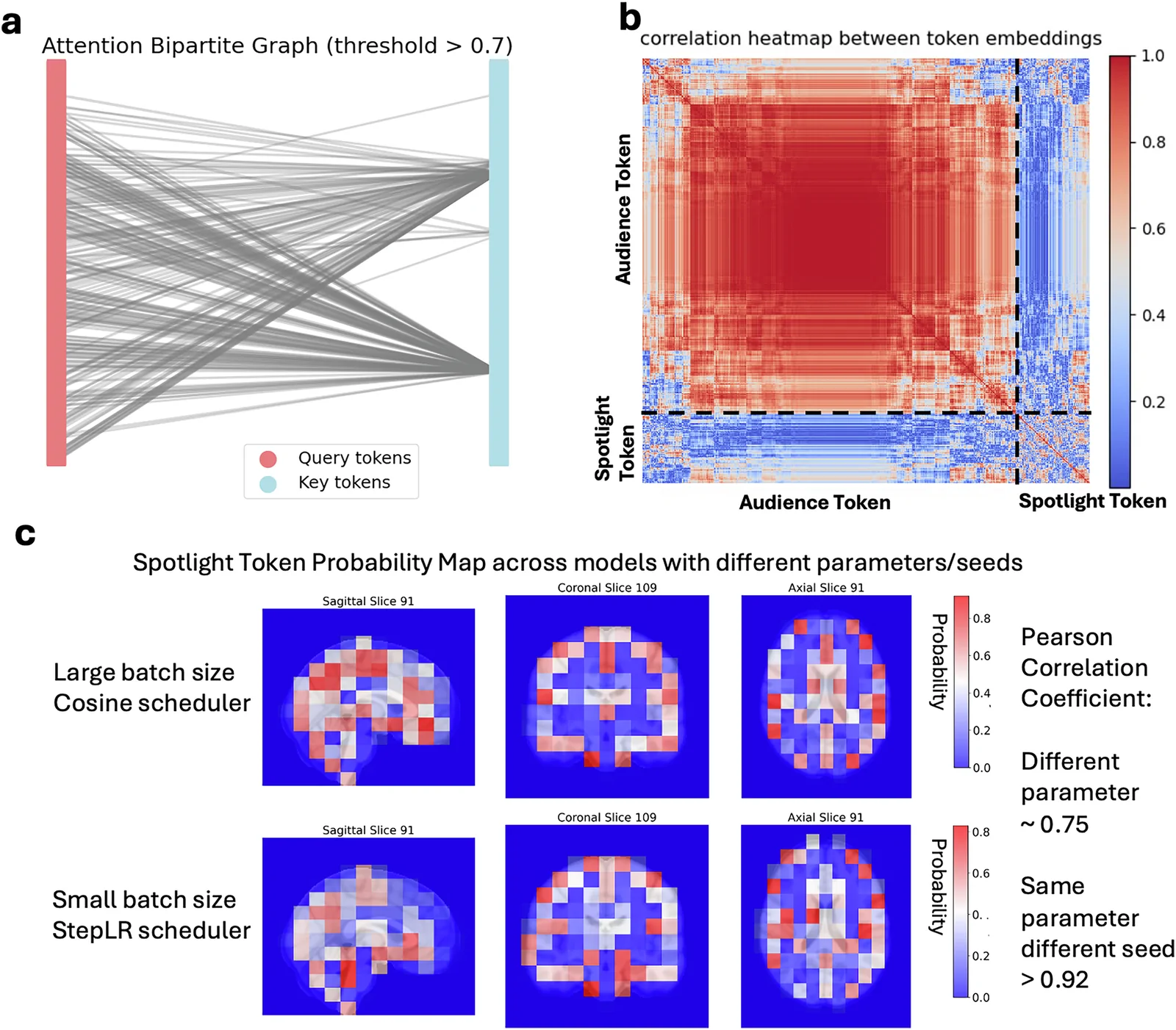
Investigation of unique patterns from ViT. **a** Softmax attention score over 0.7 is plotted between 957 tokens for attention layer 1 head 1, demonstrating that only a few tokens are strongly attended. **b** Correlation heatmap between 957 tokens input embeddings, we use hierarchical clustering with average linkage to reorder the dimensions of the heatmap. The color bar represents the pearson correlation coeffiecients, ranging from blue to red, where red indicates higher correlation. **c** Visualization of spotlight token probablity across models with different parameters and seeds, viewed in three slicers. Images reproduced by kind permission of UK Biobank ©.

We found that this behavior arises from the specific embeddings learned by the model (see Supplementary Note 5). The input embedding matrix projects the majority of patches into a similar direction, resulting in high correlations (0.81 ± 0.18), which we refer to as audience tokens. In contrast, a small subset of patches exhibits much weaker correlations both with the majority and among themselves (0.30 ± 0.20). We term these spotlight tokens (Fig. 5b).

We then visualize the spatial probability of spotlight tokens across brain slices for models trained with different parameter settings and random seeds. As shown in Fig. 5c, red regions highlight anatomical locations where the model consistently concentrates its attention across samples. The proportion of spotlight patches among all brain patches remains stable across experiments, with a sample-wise average of 18%. Spatial consistency is further supported by high Pearson correlation coefficients: correlations between models with different parameter settings are around 0.75, while models with identical parameters but different random seeds or training epochs exhibit correlations exceeding 0.92. These results indicate strong robustness and stability of the identified spotlight tokens.

We observe that these spotlight patches tend to cluster around deep sulci, align along the boundaries of the ventricles, and appear in structures such as the cerebellum and brainstem. Additionally, some spotlight patches appear in corresponding regions across hemispheres, suggesting a tendency to capture bilaterally relevant anatomical features. The distribution of attention is sparse and concentrated around major structural landmarks, indicating that the model prioritizes these regions for information aggregation.

Our observations revealed long-range dependencies and symmetrical attention across the brain regions. The model was able to attend to distant patches across different brain regions simultaneously, demonstrating its capacity to capture non-local relationships within the brain (also see Supplementary Fig 9A). Additionally, we observed symmetrical attention, with patches in one hemisphere of the brain attending to corresponding patches in the opposite hemisphere (see Supplementary Fig 9B). This symmetry suggests that the ViT-AE model is effectively capturing bilateral relationships, reflecting the natural symmetry of brain anatomy. These findings highlight the model’s ability to integrate both local and non-local features, providing a comprehensive understanding of brain architecture and uncovering complex interconnections between brain regions.

## Discussion

A central theme of our study is that a different architectural inductive bias can reveal new biological insights from the same data. We developed an approach that integrated unsupervised deep representation learning, specifically using a ViT-AE model, for brain imaging GWAS. Our ViT-AE pipeline successfully identified 24 genetic loci associated with brain structure that were missed by a previous CNN-AE-based approach, with 10 of these having no prior associations with brain morphology in the GWAS Catalog. We attribute this success to the Vision Transformer’s inherent ability to model flexible dependencies through its self-attention mechanism. Unlike CNNs, which build a hierarchy of local features, the ViT can directly capture long-range patterns, such as the left-right hemispheric symmetry and relationships between distant sulci. It is important to note that we are not claiming that ViT is superior to CNN based on these novel loci; rather, we suggest that the two approaches may be complementary in discovering genetic loci. This suggests that the genetic influence of these novel loci may manifest as subtle, distributed, or symmetrical patterns across the brain.

With regard to the reproducibility of the UDIPs, it is worth noting that individual UDIP dimensions will change when using different training datasets or even different random seeds. However, the overall multivariate distribution of UDIPs, as well as the loci discovered based on them, remains largely consistent. We showed that UDIPs are largely reproducible, as shown by the high CKA and explained variance across UDIPs derived from different training settings (seeds, data split, and an independent dataset (ADNI)). We have also done the generalized CCA^33^ to find the shared space of all UDIPs. If more strict reproducibility is needed, we can project the UDIPs onto the first 30 dimensions of the shared space, which shows an average correlation of 0.81 ( ± 0.02) between all models. This means if we keep the UDIPs in this subspace, then any two models’ latent can be related by a linear transform with small error.

The model’s ability to uncover these novel associations stems from its unique architectural properties. Our pipeline also incorporated a perturbation-based decoder interpretation approach to map these loci and their associated imaging phenotypes to specific brain regions. By comparing original and reconstructed images for two individuals and visualizing the absolute differences (see Supplementary Fig 10), we showed our model’s capacity to capture structural differences in brain morphology. Furthermore, our analysis of the model’s attention mechanism revealed the data-driven emergence of “spotlight tokens,” which receive a disproportionate amount of attention and correspond to anatomically informative regions with high structural variance, such as the boundaries between different tissue types (e.g., gray and white matter), the edges of ventricles, or the complex folding of deep sulci. The model appears to have learned to focus its attention on these information-rich patches. We also want to emphasize that attention patterns are learned, and are more than just the most prominent ones that we highlighted. There are more subtle changes - these are also captured by the model’s attention mechanisms - but are less interpretable, and thus not highlighted.

Despite these promising results, our study has limitations that present clear avenues for future work. A key topic in current machine learning research is the performance of ViT, particularly when faced with limited data. This is especially a concern in healthcare because of challenges in data availability^34^. To examine whether increasing the amount of training data would improve performance, we trained our ViT-AE model on a combined dataset consisting of both the original training and testing samples (n = 4,597 + 37,376 = 41,973). Under this setting, the model showed substantial improvement, achieving an average validation loss of 0.18 after 300 epochs, compared to 0.253 when trained on the training set alone (see Supplementary Fig 11A). In addition, the number of discovered loci increased, with 96 associated loci identified at 221 epochs (see Supplementary Fig 11B). However, these additional findings were not included in the main results. Although this decision may be conservative, we prioritized maintaining a clean and reliable evaluation of model performance.

Nonetheless, we present these findings to illustrate the ViT-AE model’s potential to perform better with a larger training dataset. To address the data-hungry nature of ViTs, self-supervised algorithms such as masked autoencoders (MAEs) have been shown to mitigate this issue^35^. While this approach was not incorporated into our current pipeline, we will consider it a promising future direction. In addition, we will consider data augmentation and synthetic data generation techniques. Data augmentation methods, such as random rotations, flips, and intensity variations, could be employed to create more diverse variations of the original brain MRI scans. Additionally, utilizing synthetic data generation techniques could allow for the creation of realistic, high-quality brain images, addressing challenges related to limited data availability.

The observation of “spotlight tokens” also has direct implications for the choice of patch size. While our chosen patch size (14×16×14) proved effective, a smaller patch size could potentially enable the model to resolve even finer anatomical details, allowing spotlight tokens to pinpoint more granular features. This might be crucial for phenotypes linked to subtle variations in cortical thickness or the integrity of smaller structures. However, smaller patches lead to a much longer sequence of tokens, making it harder for the model to train. Our preliminary result showed that without further optimization, the same structure with a smaller patch would lead to higher loss and a lower number of loci discovered. The optimal patch size should therefore find a balance between feature resolution and the ability to model global context; we leave this for future study.

Regarding the GWAS pipeline, we acknowledge that the min-P procedure is not an optimal multivariate association test. However, it provides a conservative lower bound on the number of detectable genetic associations, as it requires a locus to show strong evidence of association in at least one component after correction for multiple correlated tests. In the literature, min-P has been widely adopted as a baseline approach for multi-trait GWAS, and several more powerful methods have since been developed to improve locus discovery, including MOSTest^36^ and C-GWAS^37^. In this study, we employed min-P primarily to enable a fair comparison with our previous published CNN-based method, which used the same procedure. Investigation of alternative multivariate GWAS frameworks is an important direction for future work.

Another limitation of our study is the reliance on a single discovery cohort ( ~ 23,000 participants) for reporting GWAS results. Although this sample size provides substantial statistical power and makes spurious findings less likely, dependence on a single cohort may still limit generalizability. A key constraint is the limited availability of additional large-scale datasets that include both neuroimaging and genetic data. In practice, only a modest number of samples are currently available for replication (e.g., fewer than 10,000 remaining participants within the UK Biobank and approximately 2500 in the Alzheimer’s Disease Neuroimaging Initiative). Because GWAS performance and replicability are highly sensitive to sample size, a failure to replicate in these smaller datasets would be difficult to interpret, as it may reflect limited statistical power rather than a true lack of reproducibility.

Importantly, upcoming releases from the UK Biobank imaging cohort are expected to add approximately 20,800 additional participants with paired imaging and genetic data. These data will provide a valuable opportunity for replication in a larger, independent subset, which we plan to pursue as they become available. More broadly, an important next step will be to conduct replication analyses in cohort outside of UKB and to leverage cross-cohort harmonization efforts to further validate the identified loci.

In the GWAS results section, we discussed several genes within our identified loci that have been previously associated with brain structure and function. However, additional genes within these loci remain unexplored and may represent novel links to brain biology. Furthermore, we adopted the PerDI method, which connects specific anatomical regions in the brain to individual UDIP dimensions that, in turn, contribute to distinct genetic loci. For example, UDIP dimension 50 was found to link the paracingulate gyrus to bone mineral density. Further causal inference, such as Mendelian randomization, could help elucidate this relationship. There is also the possibility of horizontal pleiotropy, in which a single genetic variant independently affects multiple distinct traits, rather than influencing one trait through its effect on another. Analyses of this kind underscore the potential of our approach to reveal underlying biological mechanisms and guide future investigations.

## Methods

### Data selection

The MRI scans were obtained from UKB. Disjoint datasets were used for training, validation and GWAS to ensure robust model evaluation and prevent data leakage. 6,130 MRI scans were chosen for the model with mixed ethnicity and split into 75/25 training/validation sets. 4,597 scans were used for training, and 1,533 scans were used for validation.

For the GWAS analysis samples, we reused the discovery and replication cohort previously established in the lab^20^, following the framework of the BIG40 study^9^. From UKB participants with both T1-weighted MRI and genomic data available, we applied standard quality control filters for genotype and imaging, White British ancestry (UK Biobank field IDs 21000 and 22006), unrelatedness, concordant reported and genetic sex, and exclusion of withdrawn participants as of 08/21/2023. After filtering, all retained samples were of White British ancestry, forming a genetically homogeneous population. Analyses were restricted to the first visit, and the cohort was randomly split into ~2/3 discovery and 1/3 replication, matching the discovery cohort size used in BIG40. The final GWAS dataset included 37,376 samples (35,226 unique individuals; reflecting repeating visits), with 23,976 samples (22,867 individuals) in the discovery cohort and 13,400 samples (12,359 individuals) in the replication cohort.

The linearly registered T1 brain MRI scans were of dimensions 182 × 218 × 182. To allow division into equal-sized patches, these scans were padded to 182 × 224 × 182. The background voxels were already set to 0 by UK Biobank preprocessing. For the foreground voxels, we applied a z-transform to standardize the intensity values, ensuring consistency across scans and facilitating better model performance.

### ViT-AE model

The ViT-AE architecture consisted of a ViT encoder, an average pooling layer and a ViT decoder. The features from the average pooling layer, which served as a concise representation of the input, became a distinct token embedding that fed into the decoder layers along with empty token embeddings solely containing positional encoding. These combined token embeddings passed through multiple transformer layers and were decoded by a linear projection to reconstruct the input patches. MSE loss was computed between predicted patches and original patches within the batch mask. The reconstructed layer assembled and reshaped the predicted patches from the decoder into 3D images of size 182 × 224 × 182.

Our ViT-AE model was developed for representation learning from brain MRI scans and extraction of ViT-UDIPs. The initial implementation of the ViT-AE model consisted of an encoder embedding dimension of 384, and this was later changed to 128. The final pipeline included a CosineAnnealingWarmRestarts learning rate scheduler with T_0 = 10, T_mult=2, eta_min=1×10^-6^ and AdamW optimizer with an initial learning rate of 0.001.

The ViT-AE model was trained for 300 epochs on the training set and validated on a separate validation set on 4 Nvidia H100 GPUs. Additionally, we repeated the training with different seeds, different data splits (4-fold cross-validation), and different datasets (ADNI) to test the reproducibility of UDIPs with centered kernel alignment (CKA) and explained variance. We demean two UDIPs X and Y first, then perform SVD on X and Y: ${{\rm{X}}}={{{{\rm{U}}}}_{1}{{{\rm{S}}}}_{1}{{\rm{V}}}}_{1}^{{{\rm{T}}}}$, ${{\rm{Y}}}={{{{\rm{U}}}}_{2}{{{\rm{S}}}}_{2}{{\rm{V}}}}_{2}^{{{\rm{T}}}}$, explained variance of X by Y is $\frac{|\left({S}_{1}U\right)\odot S{|}_{F}^{2}}{|{S}_{1}{|}_{F}^{2}}$, and explained variance of Y by X is $\frac{|\left({S}_{2}U\right)\odot S{|}_{F}^{2}}{|{S}_{2}{|}_{F}^{2}}$, where ⊙ denotes elementwise multiplication and *F* denotes the Frobenius norm. GWAS was later performed on the held-out test set using the ViT-UDIPs extracted by the trained model.

### GWAS

Related individuals were filtered out since our GWAS was conducted using a linear model. Additionally, multiple visits were filtered where only scans from the first visits were retained. A total of 22,867 subjects were used for performing GWAS on the extracted ViT-UDIPs. GWAS was performed between the SNPs and the 128 ViT-UDIPs, with age (field ID 21003), age^2^, sex (field ID 31), sex × age, sex × age^2^, 10 genetic PC (field ID 22009), head size (field ID 25000), inverted contrast-to-noise ratio (field ID 25735), head position in scanner (field ID 25756-25758), scanner table position (field ID 25759), location of the assessment center (field ID 54) and date of attending assessment center (field ID 53) as covariates. The GWAS result without head size covariate can be found in the Supplementary Fig 12. FastGWA^38^, implemented in GCTA (Genome-wide Complex Trait Analysis, Version 1.94.1), was used to perform linear mixed model association analyses. The Bonferroni-corrected significance threshold was set to 5×10^-8^/128. We created a summary statistics file with only the most significant p-value (minP) for each SNP. This file was uploaded and run on FUMA SNP2GENE^39^ and the generated GenomeRiskLoci.txt file was downloaded to get the clumped loci.

### Querying GWAScatalog traits from FUMA

We used FUMA to query candidate SNPs in each locus in GWAS to identify previously reported association traits from the downloaded gwascatalog.txt. We identified brain-related traits from the GWAS catalog results using careful manual inspection.

### LDSC-estimated SNP heritability

We used the LDSC v 1.0.1(https://github.com/bulik/ldsc) to calculate the SNP heritability of UDIPs. The LD scores were calculated from 1000 Genomes. All these values were computed from the discovery cohort, with the sample sizes of 22,867.

### Loci overlapping

For identifying the number of loci corresponding to the IDP loci panel, we referenced the BIG40 database—a comprehensive GWAS study conducted on approximately 40,000 UK Biobank participants^9^. The study analyzed 3,929 IDPs (https://open.win.ox.ac.uk/ukbiobank/big40/). From these, we selected the first 1,437 IDP summary statistics derived from T1 and T2 modalities. We then performed a minP analysis across these IDPs for all SNPs, followed by the FUMA procedure described above, using a Bonferroni-corrected significance threshold of 5×10^-8^/1437 to obtain a set of genomic loci. Each locus was then extended by 125 kb on both sides, and an interval tree was constructed for each chromosome. The loci identified from our analysis were subsequently queried against these interval trees to determine overlaps.

### Define novel loci

Using the loci-overlap procedure described above, we compare loci identified from ViT-UDIPs with those from CNN-UDIPs, as both are derived from deep learning features. Loci detected by ViT-UDIPs that do not overlap with CNN-UDIPs are defined as novel loci. We further perform a three-way overlap analysis and present the loci unique to ViT-UDIPs relative to both CNN-UDIPs and IDPs (Supplementary Fig 13).

### PerDI

To visualize the brain regions associated with the extracted ViT-UDIPs, we utilized a perturbation-based approach^20^. For 500 subjects from the test set, we added 1 standard deviation of noise to the 128 ViT-UDIPs. The decoder was then used to reconstruct the images based on the perturbed ViT-UDIPs, followed by a voxel-based paired t-test.

A mask based on a brain template linearly registered to the T1 MNI152 space was applied to the t-test results, followed by a Gaussian filter (sigma=3) for smoothing. The resulting images from perturbed ViT-UDIPs, were saved as NIFTI files for visualization using ITK-SNAP. These NIFTI files were also used to find the regions of intersection with the Harvard-Oxford Cortical and Subcortical Structural Atlases (RRID:SCR_001476)^40–43^ and MNI Structural Atlas^44,45^.

### Sparse attention patterns analysis and visualization

To qualitatively analyze the sparse attention patterns learned by the ViT, we use the final trained checkpoint and extract the self-attention score matrices (957 × 957 tokens) from each layer and head. These attention scores are normalized using a softmax operation within each layer and head. We then threshold the normalized attention scores at 0.7 and visualize the resulting connections as a bipartite graph, focusing on layer 1, head 1.

To further investigate relationships among tokens, we take the 957 input token embeddings and apply hierarchical clustering with average linkage. This clustering is used to reorder the heatmap dimensions, enabling clearer identification of “spotlight tokens”.

We then evaluate the stability of spotlight tokens across 500 randomly sampled inputs. We first examine the relative frequency within brain tokens, i.e., for each sample, the proportion of spotlight tokens among all brain tokens, to ensure that the observed patterns are not driven by variations in token composition across samples. Second, we examine positional consistency by constructing a probability heatmap that quantifies how frequently each token index is identified as a spotlight token across samples. We then assess robustness by computing the Pearson correlation between probability heatmaps obtained from multiple training runs, including runs with different random seeds and different hyperparameter settings.

### Statistics and Reproducibility

In general, GWAS analyses use a t-test to determine whether SNP effects are statistically significant. In this study, we used n=22,867 samples to perform the t-tests with the conventional genome-wide significance threshold of $p < 5\times {10}^{-8}$. To account for the multivariate analysis across 128 phenotypes, we further applied a Bonferroni-corrected threshold of $5\times {10}^{-8}/128$. For the PerDI t-maps, paired t-tests were conducted on 500 samples by comparing the permuted reconstructions with the original reconstructions. Pearson correlation coefficients (r) were used in multiple analyses to assess correlations.

We evaluated reproducibility only under the final optimized training setting by varying the training inputs across 4 runs and the random initialization across 4 runs. The UDIPs demonstrated strong reproducibility across experiments (Supplementary Fig 3-6).

### Ethics oversight

Our analysis was approved by the UTHealth Houston committee for the protection of human subjects under No. HSC-SBMI-20-1323. UKB has secured informed consent from the participants in the use of their data for approved research projects. UKB data was accessed via approved project 24247.

### Reporting summary

Further information on research design is available in the Nature Portfolio Reporting Summary linked to this article.

## Supplementary information

Supplementary Information

Supplementary Data 1-6

Reporting Summary

Transparent Peer Review file

## Data Availability

The MRI imaging and genomic data could be accessed by requesting UKB. The deep learning model checkpoints and GWAS analysis summary statistics, along with source data underlying figures, could be accessed on https://zenodo.org/records/20225687^46^ and with GCP id GCP001685 on GWAS catalog https://www.ebi.ac.uk/gwas/.

## Code availability

The code is available at the github repo: https://github.com/ZhiGroup/UDIP-ViT and Zenodo: https://zenodo.org/records/20225687^46^.
