# Supplementary material for: Vision transformer autoencoders captures local and non-local features in brain imaging to reveal novel genetic associations
Source: Commun Biol. 2026 Jun 9;9:1282. doi: 10.1038/s42003-026-10430-6 (PMC13635212; doi:10.1038/s42003-026-10430-6)
Supplement: Supplementary file 1 [file 42003_2026_10430_MOESM1_ESM.pdf]

**Supplemental Materials for Vision Transformer Autoencoders Captures Local and Non-Local Features in Brain Imaging to Reveal Novel Genetic Associations**

Samia Islam, Tian Xia, Wei He, Ziqian Xie, Degui Zhi

1. Supplementary Notes
2. Supplementary Figures

## Supplementary Notes

### Supplementary Note 1: ViT dimensions

Our initial ViT-AE model had 384 encoder embedding dimensions, resulting in 384 ViT-UDIPs. We performed statistical analysis on these extracted features to find their cross correlation. Strong cross-correlation of features (correlation coefficient > 0.8) indicated embeddings collapsing, revealing that the model's latent space was not well-separated, likely causing poor generalization to unseen data. Reducing the dimensionality of the model by adjusting the model's architecture to 128 embeddings allowed better feature separation, where the model was able to distinguish between different aspects of the data more effectively, thus minimizing overfitting (see Supplementary Figure 14).

### Supplementary Note 2: UDIP reproducibility

To demonstrate the reproducibility of these UDIPs, we trained the network under three sources of variability: different training seeds, different data splits, and distinct datasets (ADNI) with varying patient backgrounds. The results show strong alignment in UDIP distributions (as measured by CKA and CCA) and a high degree of overlap in the identified loci. Specifically, CKA (range 0 to 1) achieves an average of 0.83 across different training seeds, 0.80 across different data splits, and 0.60 across distinct datasets. CCA between UDIPs shows strong alignment, with correlations exceeding 0.8 for the first 30 canonical components, indicating that these components can be linearly transformed to closely match one another and are therefore effectively interchangeable. Moreover, the explained variance of one UDIP by another averages around 0.7, where explained variance is defined as the fraction of the total variance in X that is shared with Y. The overlap analysis shows an average of 37 loci for different training seeds, 35 for different data splits, and 17 for distinct datasets. Additional figures are provided in Supplementary Figures 3–6.

### Supplementary Note 3: Intepretation on genes highlighted

FOXP1 encodes a transcription factor highly expressed in the neocortex, where it regulates cortical neuron migration, dendritic morphogenesis, and subtype differentiation, thereby shaping early neuronal circuitry [1,2]. Pathogenic variants in FOXP1 cause a monogenic neurodevelopmental disorder known as FOXP1 syndrome, characterized by intellectual disability, speech and language delay, autism spectrum features, and global developmental impairment, underscoring its essential role in cortical and cognitive development [3].
TCF7L1, a transcription factor acting downstream of Wnt/β-catenin signaling, modulates neural progenitor proliferation and differentiation, pathways that are vital for forebrain patterning and synapse formation [4]. Dysregulation of Wnt pathway components, including TCF7L1, has been associated with autism spectrum disorder and septo-optic dysplasia, indicating its indirect yet

significant contribution to neurodevelopmental integrity. Similarly, SLC39A8 encodes a divalent metal ion transporter responsible for manganese and zinc homeostasis, essential cofactors in enzymatic and neurotransmission processes within the brain [5]. Loss-of-function mutations in SLC39A8 cause congenital disorders of glycosylation (SLC39A8-CDG), featuring intellectual disability, developmental delay, and cerebellar hypoplasia, reflecting its indispensable role in neurochemical and metabolic regulation [6].

Beyond neuronal and metabolic mechanisms, CEP120 and ANGPT1 illustrate structural and vascular dimensions of brain development. CEP120 encodes a centrosomal protein highly expressed in neural progenitor cells. Knockdown of CEP120 disrupts interkinetic nuclear migration (INM) and causes premature neural progenitor differentiation, thereby reducing the progenitor pool during cortical development [7]. Mutations in CEP120 are associated with ciliopathies and microcephaly, demonstrating that centrosomal dysfunction disrupts neuronal progenitor proliferation and migration [8]. Meanwhile, ANGPT1 (angiopoietin-1), though primarily known for vascular maturation, exhibits expression in glial and neuronal progenitors, contributing to neurovascular unit formation and blood-brain barrier stability [9]. The interplay between vascular development and neuronal differentiation mediated by ANGPT1 emphasizes the integrated nature of neurodevelopmental and angiogenic signaling.

**Supplementary Note 4: Statistical analysis of reconstruction loss**

Our ViT-AE model was trained on 4,597 T1-weighted brain MRI scans, while the validation set was of length 1,533. Inference and subsequent GWAS were performed on 22,867 out of 37,376 scans, following Elliott et al's UKBB study. We performed basic statistical analysis of reconstruction loss and outliers in the dataset to gain insights into the model's performance. Overall reconstruction loss was found to be distributed normally, suggesting the model performed consistently across most scans in the dataset (see Supplementary Figure 15A). Boxplot and scatter plots indicated outliers that might have contributed to higher average reconstruction loss for some scans (see Supplementary Figure 15B, 15C). For the sake of efficiency, we considered only extreme outliers, which we defined to be outside of lower quartile (Q1) and upper quartile (Q3) by three times the inter-quartile range (IQR) (see Supplementary Figure 15D) and visualized those with the highest reconstruction losses to determine if there were significant anomalies in the brain structures or artefacts introduced by motion during scanning (see Supplementary Figure 16). Analysis of ViT-UDIPs obtained from inferencing demonstrated a normalized distribution for each ViT-UDIP (see Supplementary Figure 17). Lines in violin plot for the ViT-UDIPs represented median, Q1 and Q3 values and each violin width represented kernel density estimate (KDE) of the data. The overall shapes indicated overall normality of the ViT-UDIPs.

**Supplementary Note 5: Explanation of the sparse attention pattern**

The direct cause of the sparse attention pattern is the clustering of the audience token embeddings. At each attention head, the (unnormalized) attention score between two tokens $X_1$ and $X_2$ can be written as $X_1^T W_K^T W_Q X_2$, this can be interpreted as first rotating them differently (based on the rotation matrices formed by the left and right singular vectors of $W_K^T W_Q$), followed by projecting the rotated $X_1$ and $X_2$ on to the dimensions with nonzero singular values and finally computing a weighted inner product. Specifically, singular value decomposition of $W_K^T W_Q = U\Sigma V^T$, $U$, $V$ are rotation matrices and $\Sigma$ is a diagonal scaling matrix. The interaction between $X_1$ and $X_2$ can be written as $(PU^T X_1)^T \Sigma_r (PV^T X_2)$, where $P$ is a wide identity matrix that selects the first $d_h$

coordinates of the input, the dimension of the head. $\Sigma_r$ is the reduced singular value matrix which only consists of nonzero entries of $\Sigma$ of shape $d_h \times d_h$. As a result, at each attention head, audience tokens tend to focus on one or a few nearby spotlight tokens that happen to align with them through rotations, leading to sparse attention patterns and accumulation of information from these spotlight patches.

**Supplementary Figures**

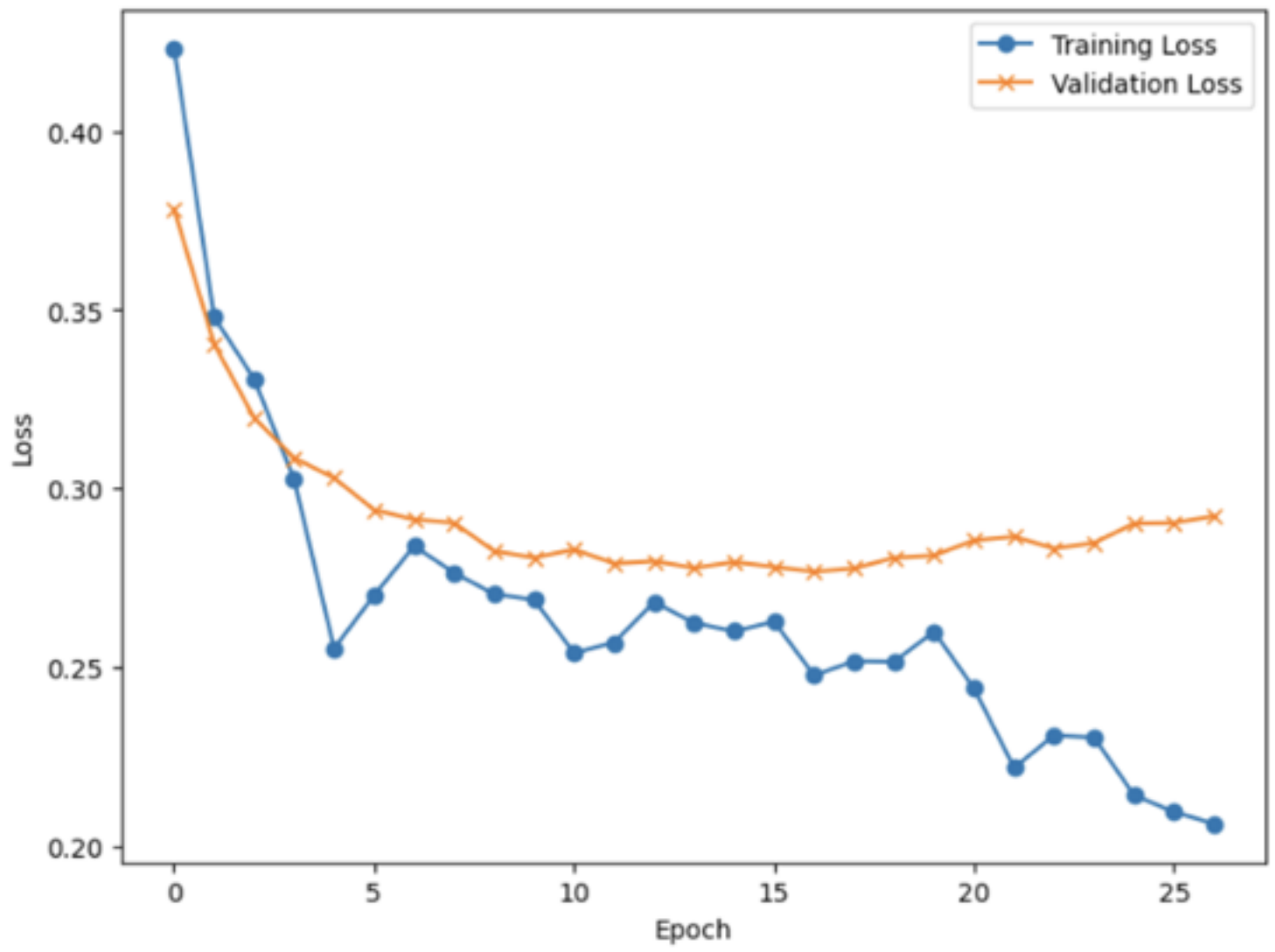


**Supplementary Figure 1 | Training and validation loss of CNN model for UDIP-based GWAS (train n=4,597, validation n=1,533).** Average validation loss was 0.28 after approximately 15 epochs, before exhibiting overfitting.

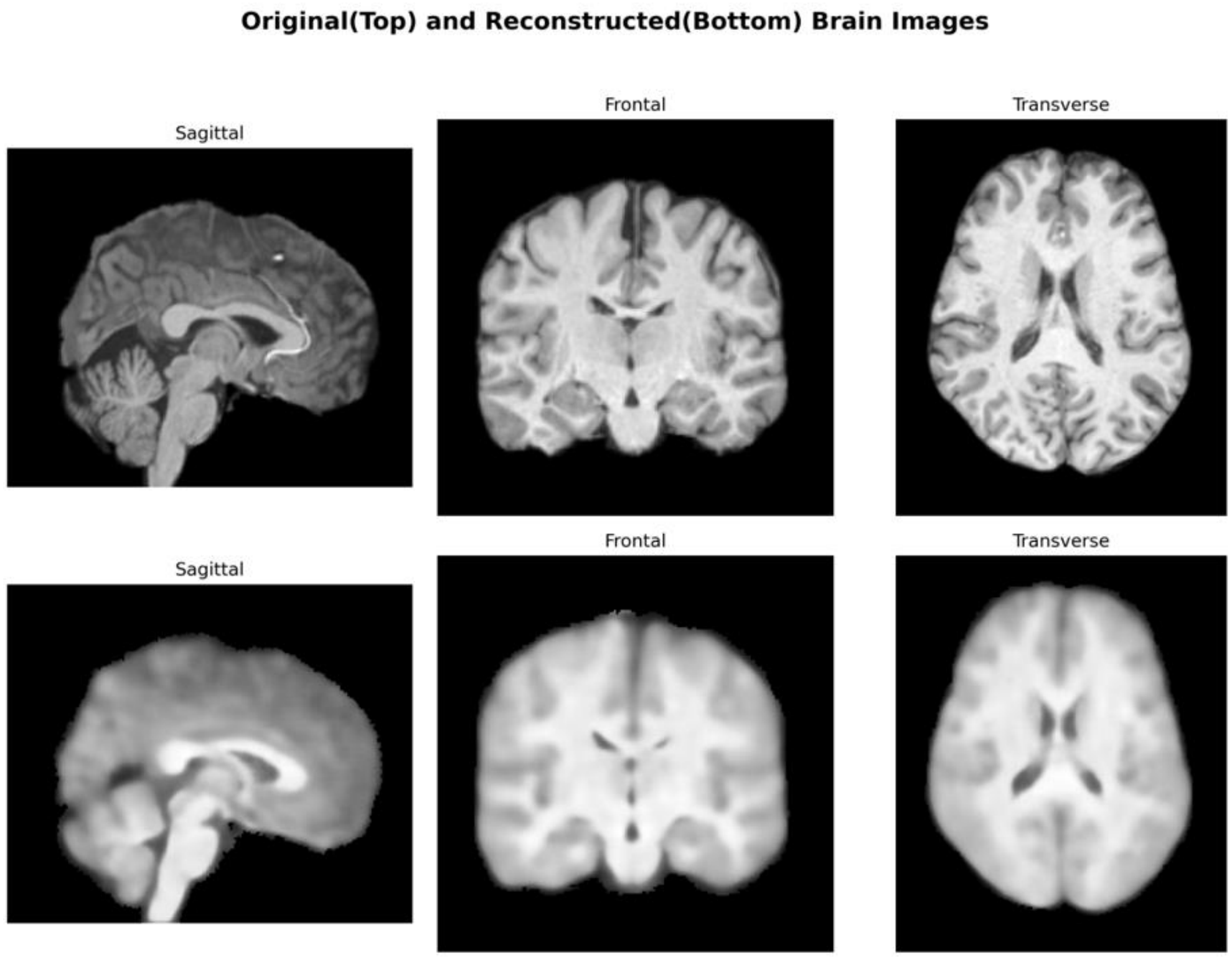


**Supplementary Figure 2 | Original and reconstructed brain images from CNN model for UDIP GWAS.** Visual observation of reconstructed images show similarity to ViT-AE model.

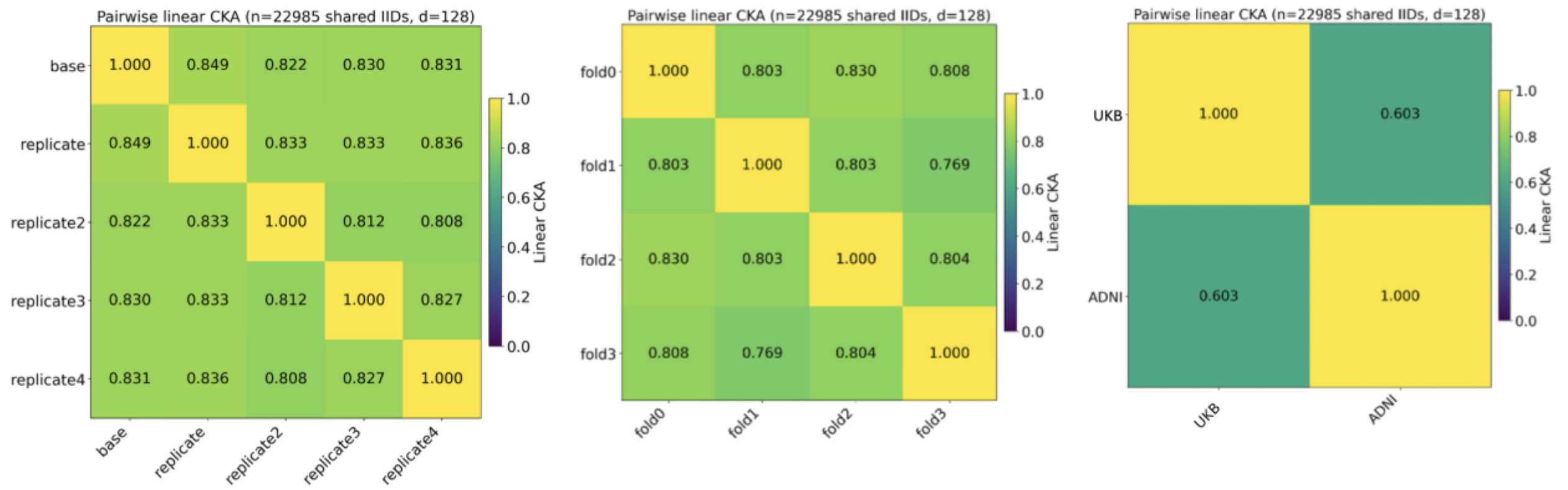


**Supplementary Figure 3 | CKA between UDIPs (from n=22,867 samples) derived from models trained with different random seeds, data splits, and distinct datasets. CKA is a similarity metric that quantifies the alignment between representations.**

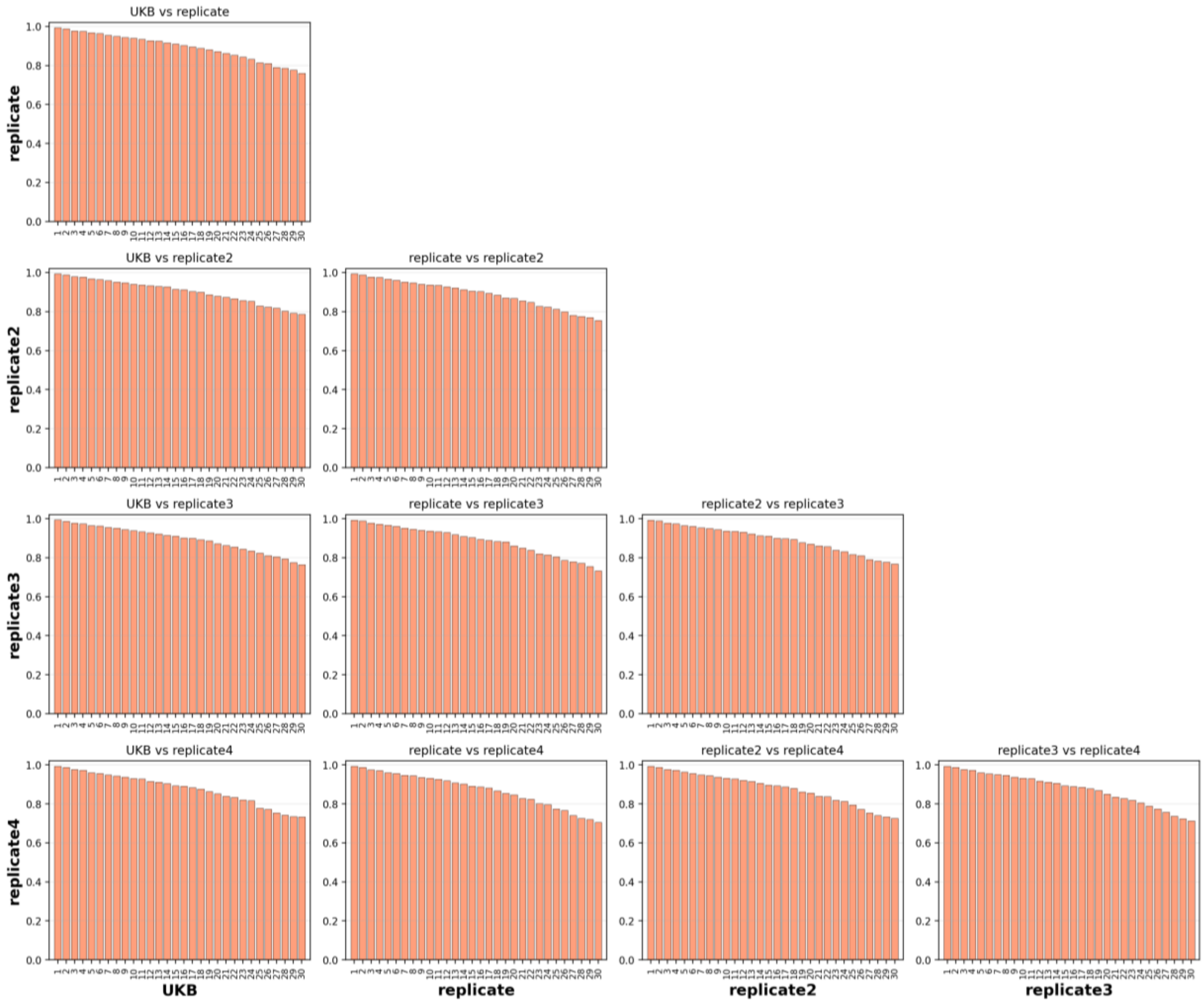


**Supplementary Figure 4 | CCA between UDIPs (from n=22,867 samples) derived from models trained with 4 more different random seeds.** The first 30 CCA correlation coefficients are plotted. The large area under bar shows great replicability between UDIPs under different training conditions.

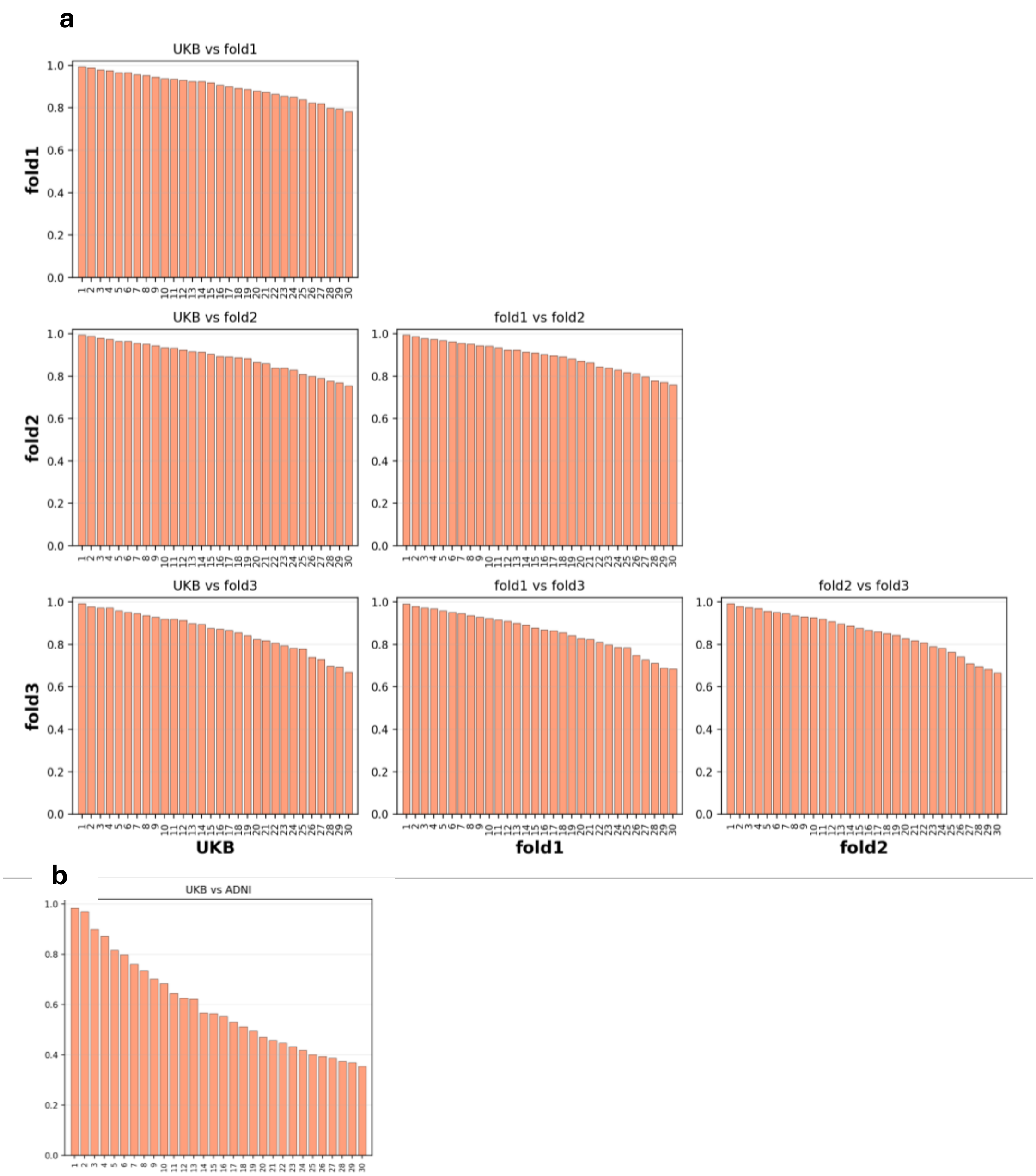


**Supplementary Figure 5 | CCA between UDIPs (from n=22,867 samples) derived from models trained with 3 more data splits (a), and 1 more distinct datasets (b).** The first 30 CCA correlation coefficients are plotted. The large area under bar shows great replicability between UDIPs under different training conditions.

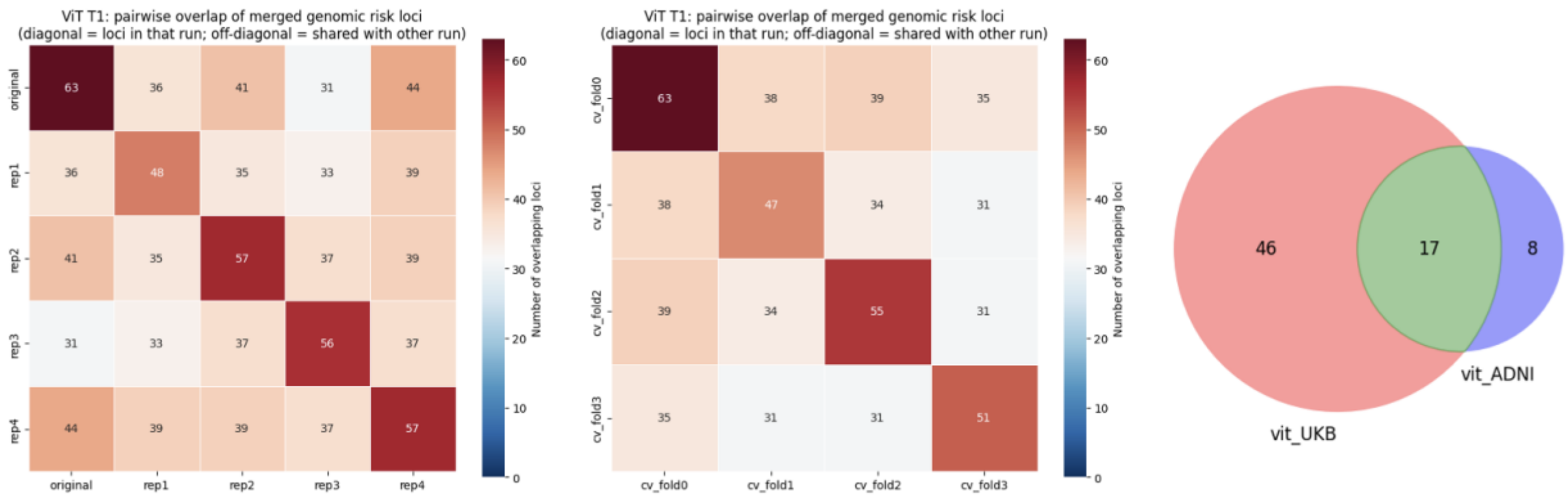


**Supplementary Figure 6 | Loci overlap between UDIPs (from n=22,867 samples) derived from models trained with different random seeds, data splits, and distinct datasets.**

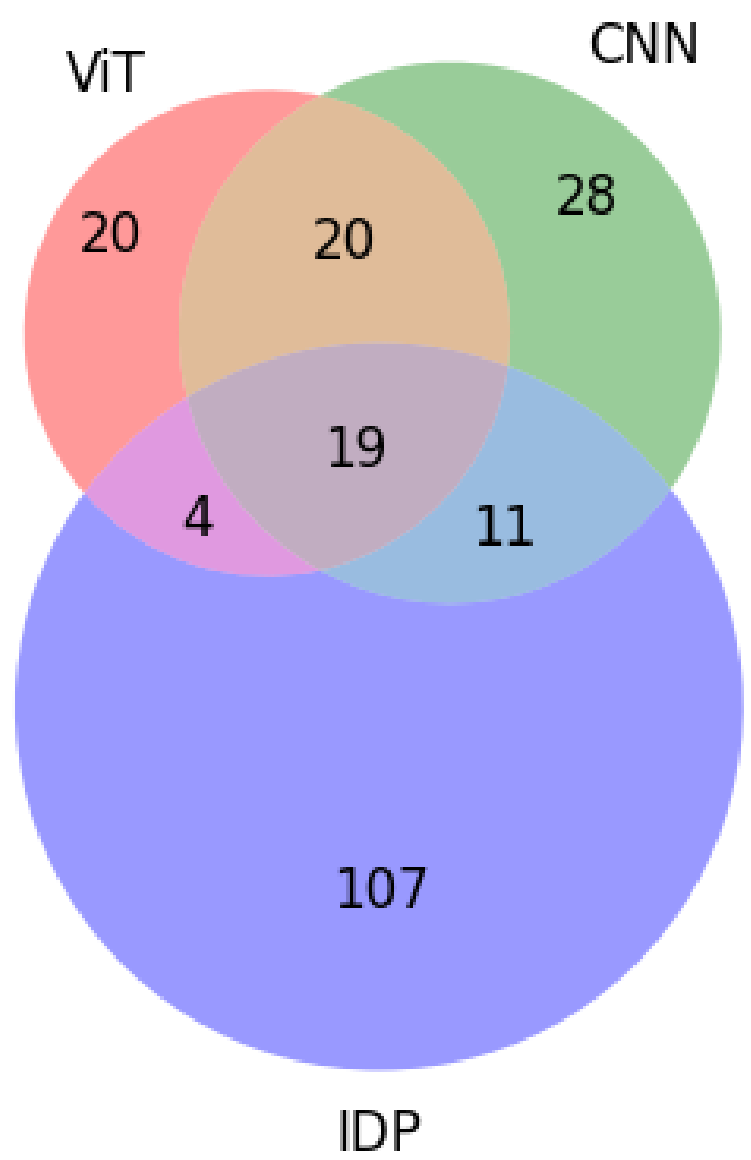


**Supplementary Figure 7 | 20 novel loci discovered by ViT-UDIPs relative to both CNN-UDIPs and IDPs**

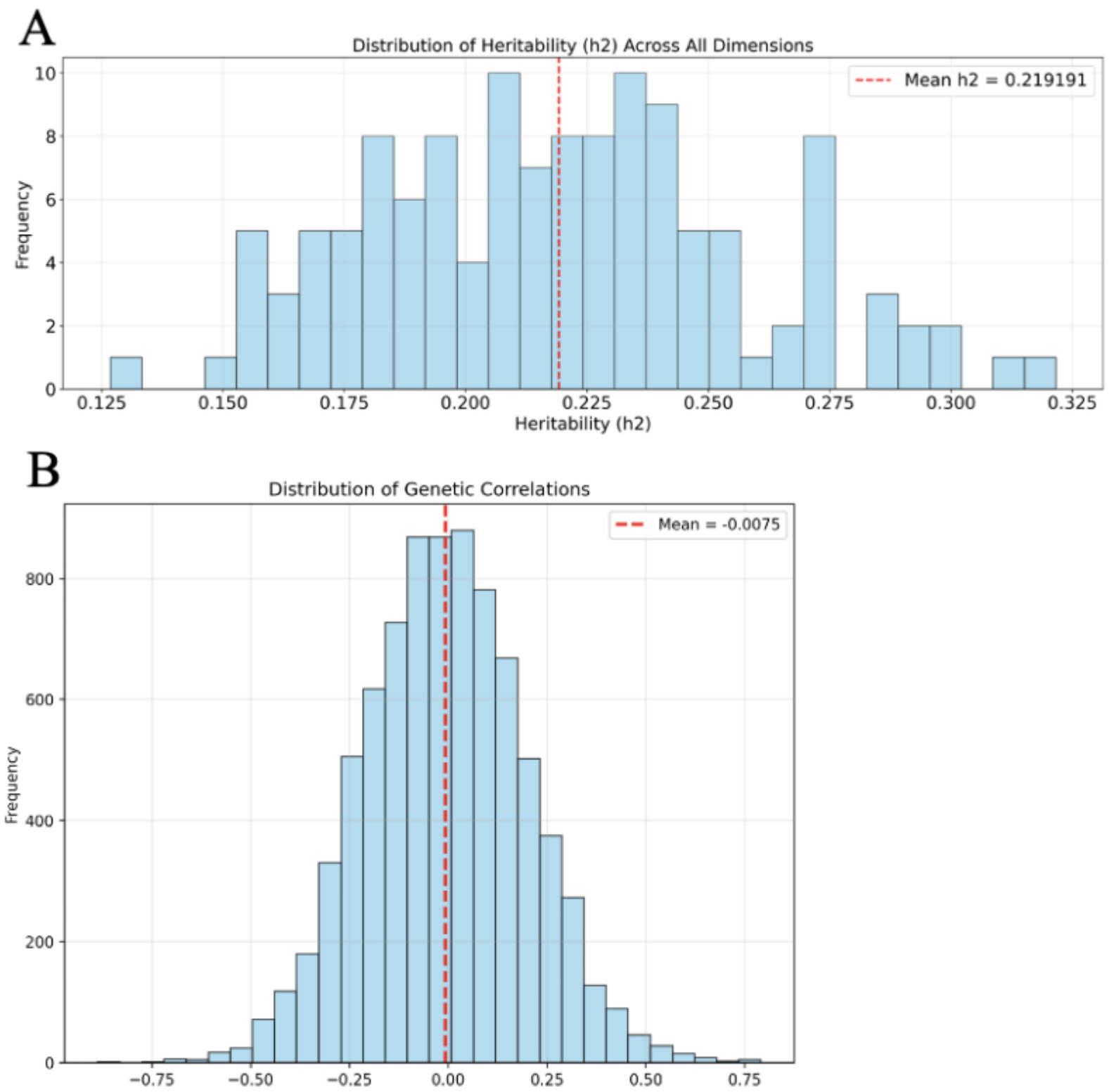


**Supplementary Figure 8 | ViT-AE model GWAS analysis summary statistics (n=22,867).** A) LDSC-estimated SNP heritability distribution across all 128 dimensions. B) Distribution of genetic correlations between every two dimensions (total pairs = 128x128/2). These two analyses were conducted using LDSC v1.0.1.

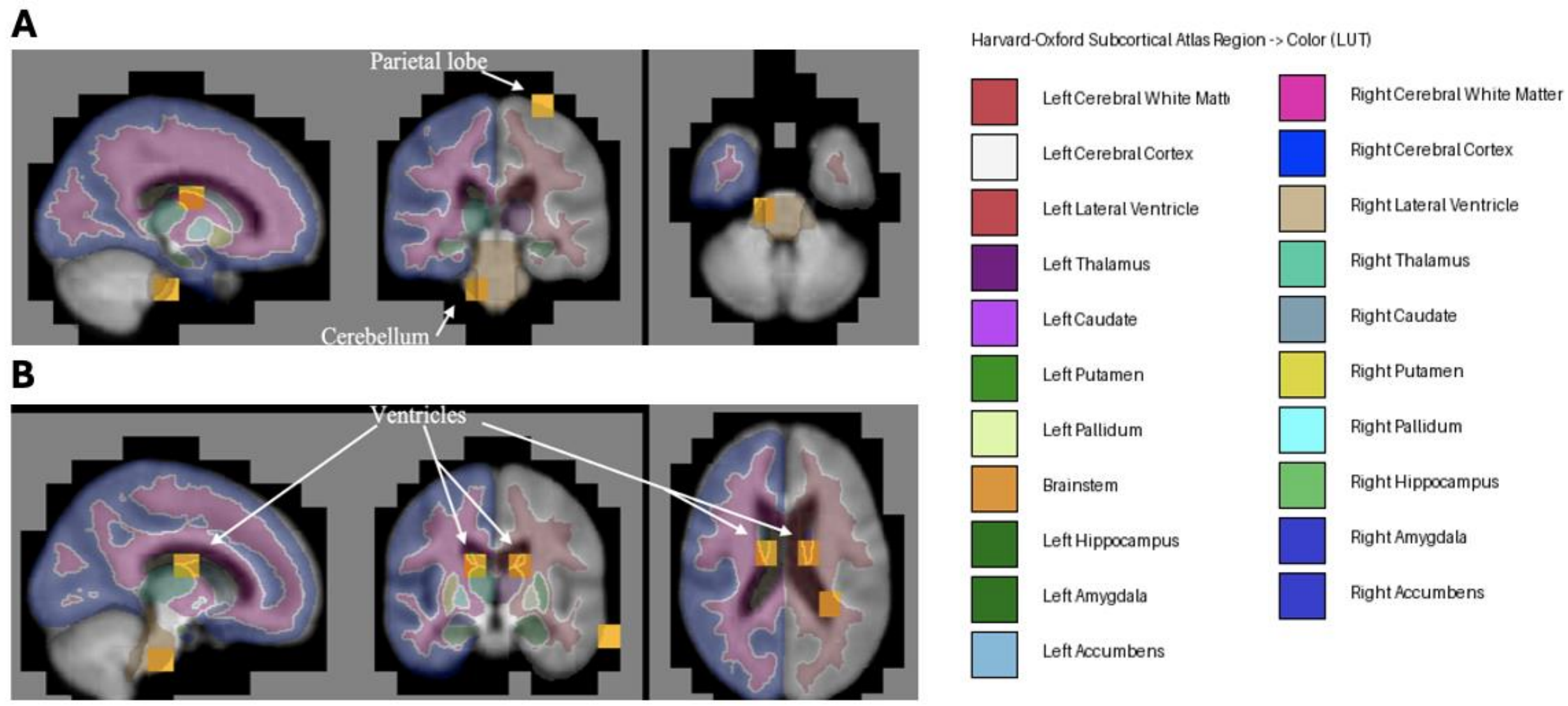


**Supplementary Figure 9 | Attention head analysis. A)** Attention heads revealed long-range regions from across the brain like structural regions of cerebellum and parietal lobe. **B)** Symmetrical regions were captured by ViT attention heads, such as left and right ventricles at the subcortical level.

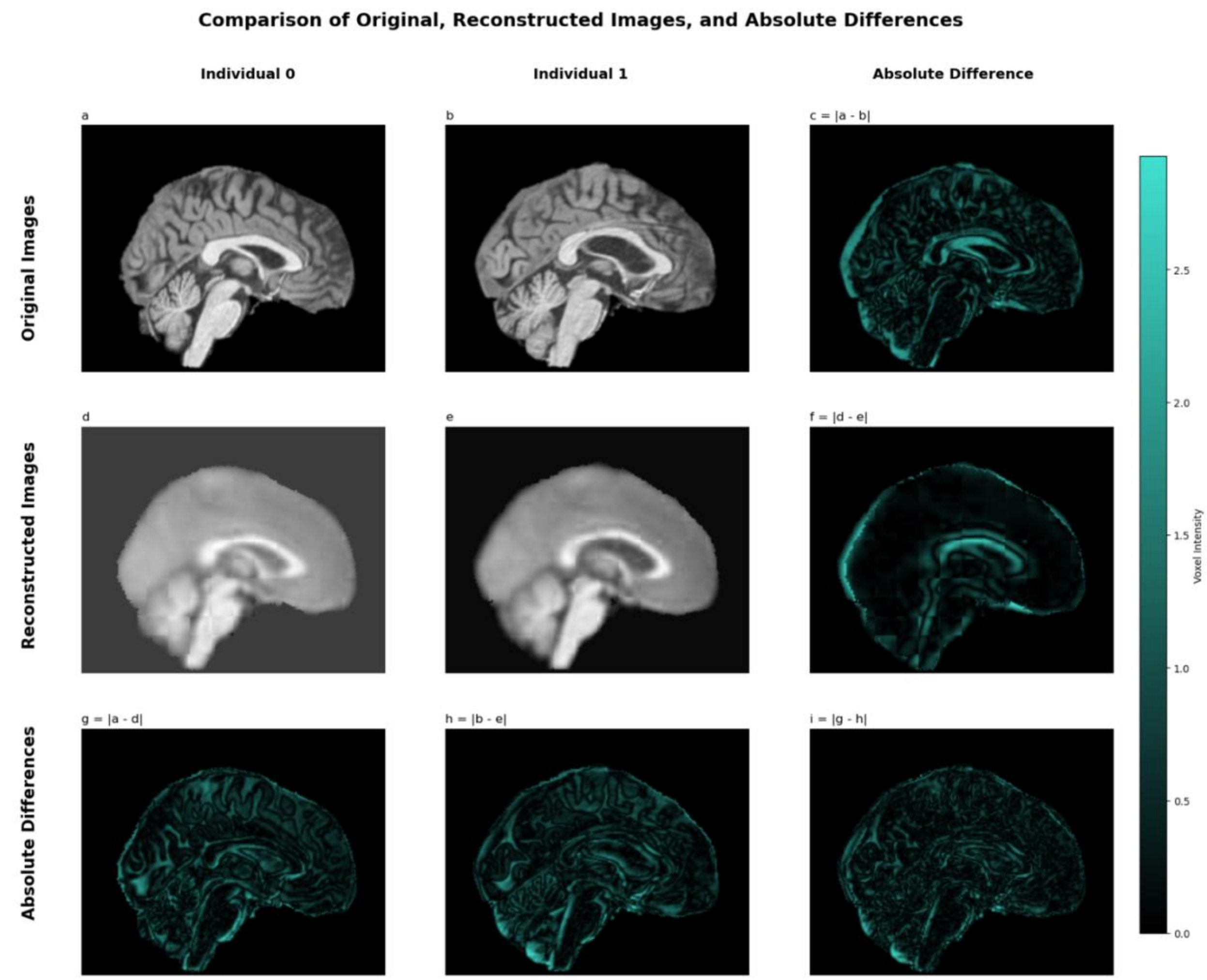


**Supplementary Figure 10 | Original, reconstructed and absolute differences between two individuals.** This figure shows reconstruction difference between Individual 0 and Individual 1. Additionally, differences between reconstructed and original images for Individual 0 and Individual 1 are compared.

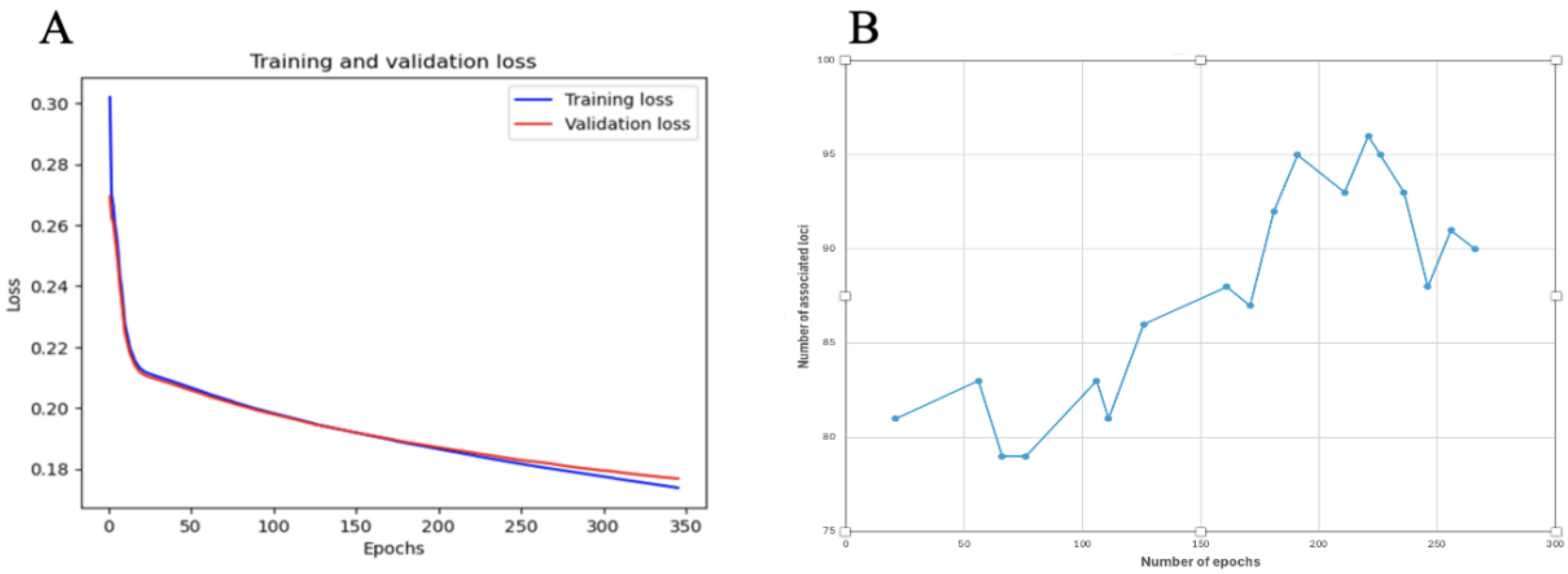


**Supplementary Figure 11 | Reconstruction performance and loci discovery of base ViT-AE model with batch size 2 and stepLR optimizer, trained on combined training and testing datasets (n=4,597+37,376=41,973).** A) Validation set remained disjoint from the combined datasets the model was trained on. However, average validation loss showed significant improvement compared to the model trained solely on the testing data. This demonstrated that a larger dataset would allow the ViT-AE to leverage its capacity more effectively, resulting in improved performance. B) Additionally, more associated loci were discovered by this trained model. However, these results were not included in our final pipeline because the GWAS was performed on the testing dataset, which was also used to train the model. This overlap could have led to data leakage, potentially inflating the model's performance and undermining the validity of the findings.

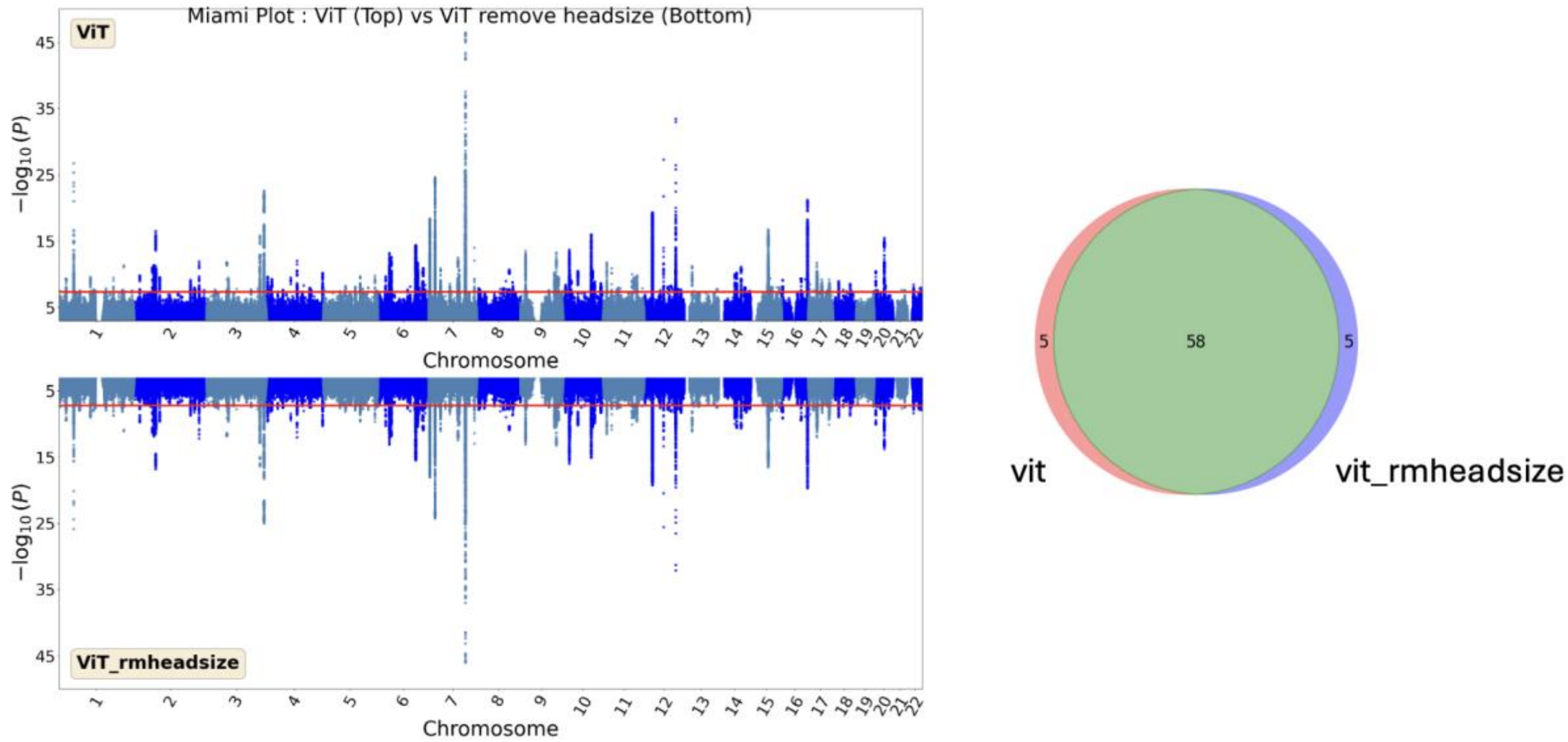


**Supplementary Figure 12 | Impact of removing head size covariate on ViT-UDIP GWAS results (n=22,867).** The left panel shows a Miami plot comparing the ViT-UDIP fastGWA summary statistics with head size included as a covariate (top) versus excluded (bottom). The right panel presents the overlap of identified loci between the two GWAS analyses, highlighting the consistency of genetic findings with and without head size covariate.

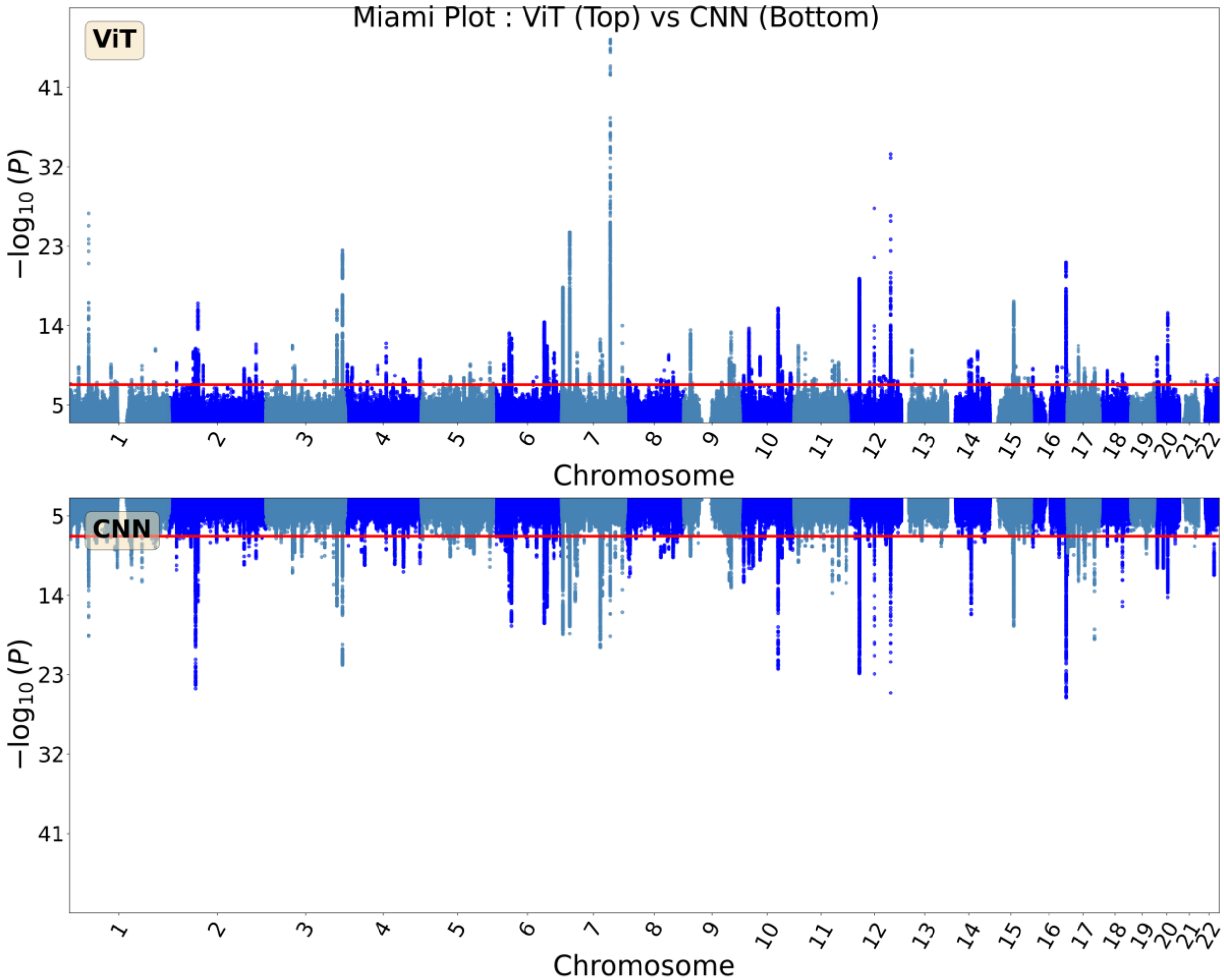


**Supplementary Figure 13 | ViT and CNN model GWAS analysis result (n=22,867) shown in the Miami plot. Red line represents the p-value significance level of 5e-8.**

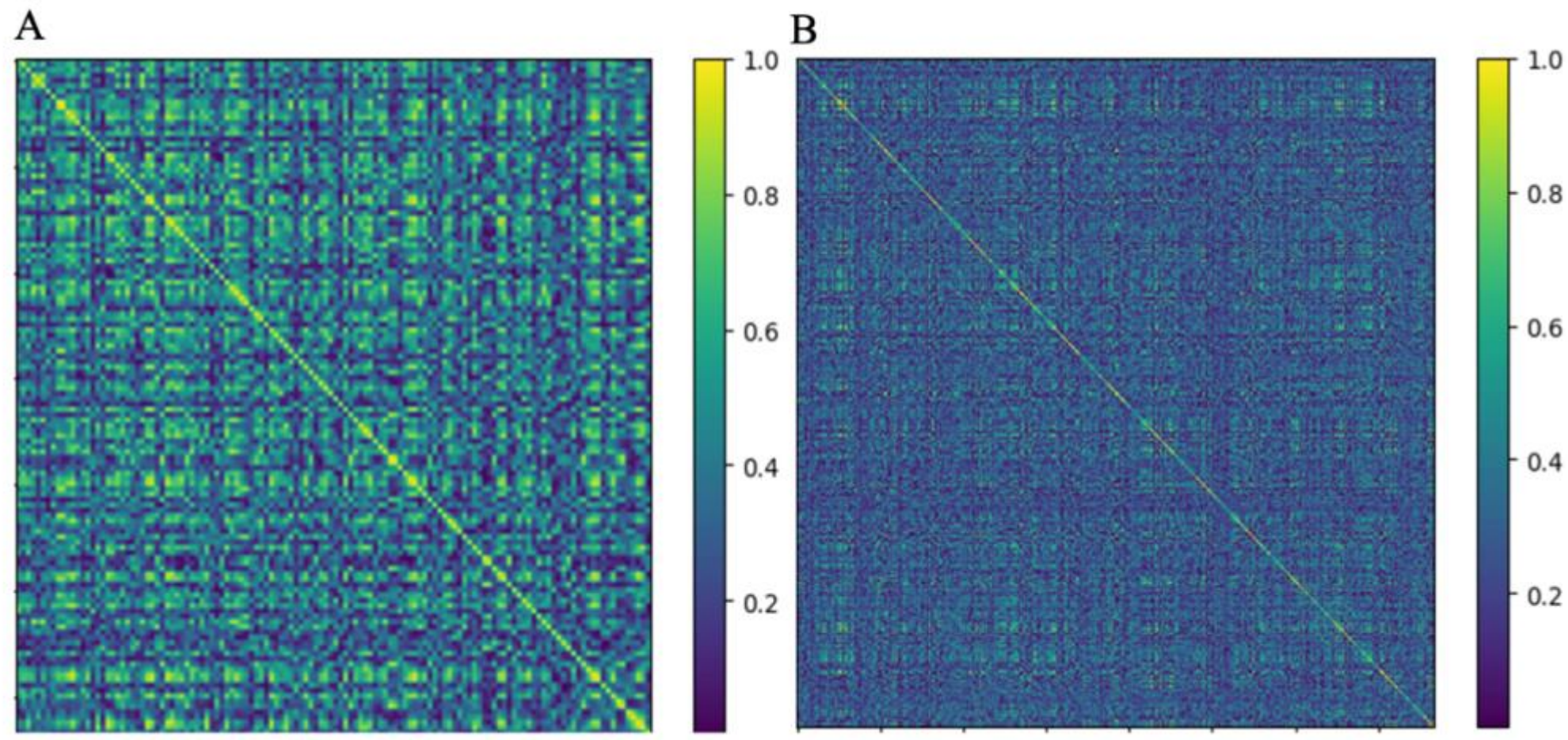


**Supplementary Figure 14 | Correlation coefficients of ViT-AE embeddings (n=22,867) showing feature collapse in higher-dimensional ViT-AE. A.** The 384-dimension ViT-AE had strong correlation among embeddings that were possibly leading to over generalization of representations, thus resulting in poor model performance. **B.** The 128-dimension ViT-AE demonstrated less correlation resulted in better feature separation, allowing the model to distinguish between different aspects of the data more effectively, thus reducing the risk of overfitting. The color bar represents the pearson correlation coefficients, ranging from blue to yellow, where yellow indicates higher correlation.

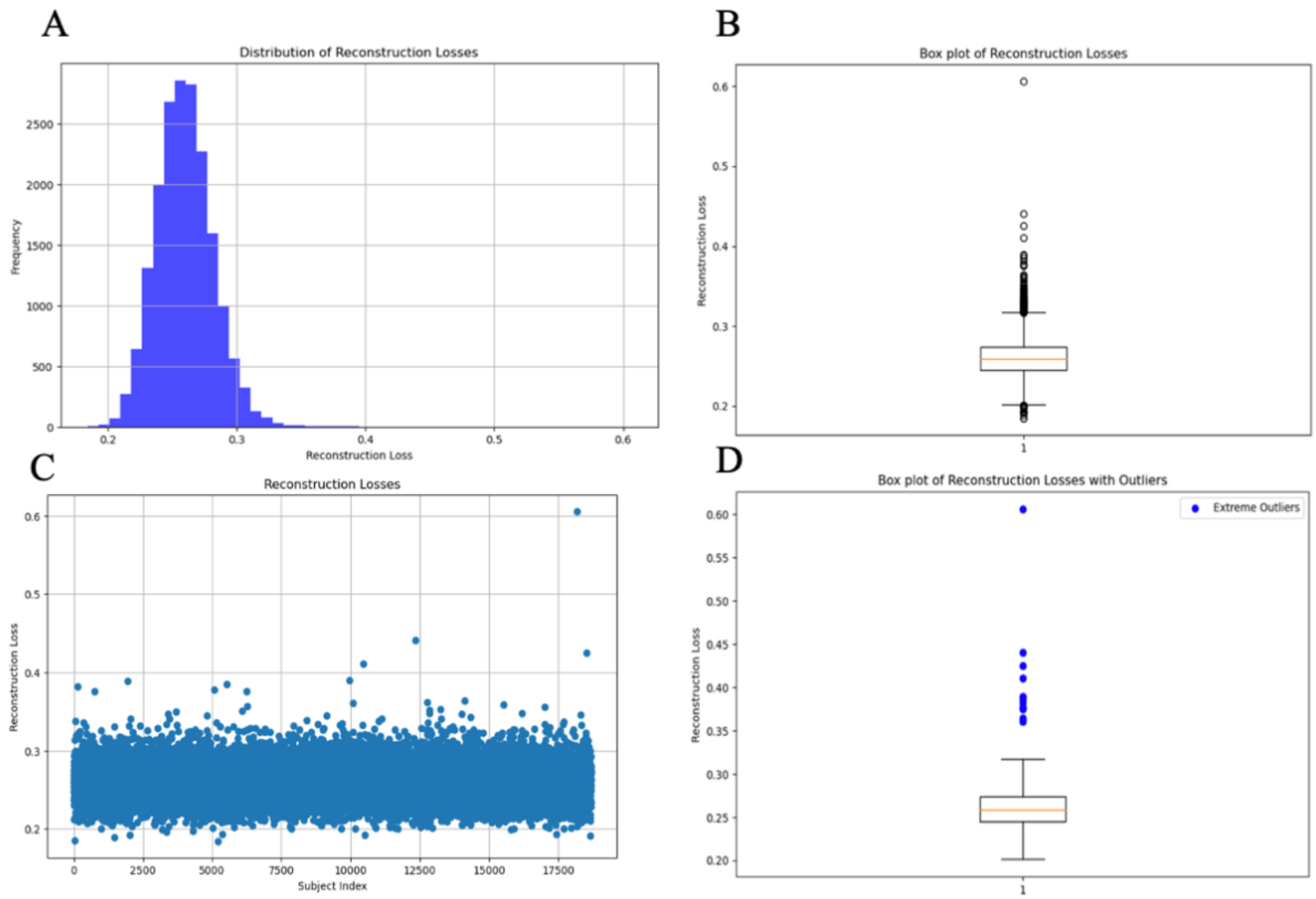


**Supplementary Figure 15 | Statistical analysis of reconstruction loss (n=22,867). A.** Overall distribution shows consistency in reconstruction loss for most images. **B.** Box plot shows outliers for reconstruction loss, which are outside of Q1 and Q3 by 1.5 times of IQR(Q3-Q1). The error bar showing is [Q1-1.5*IQR, Q3+1.5*IQR]. **C.** Scatter plot shows batch-wise reconstruction loss variation among the test dataset. **D.** Box plot shows extreme outliers for reconstruction loss, which are outside of Q1 and Q3 by 3 times of IQR. The error bar showing is [Q1-1.5*IQR, Q3+1.5*IQR].

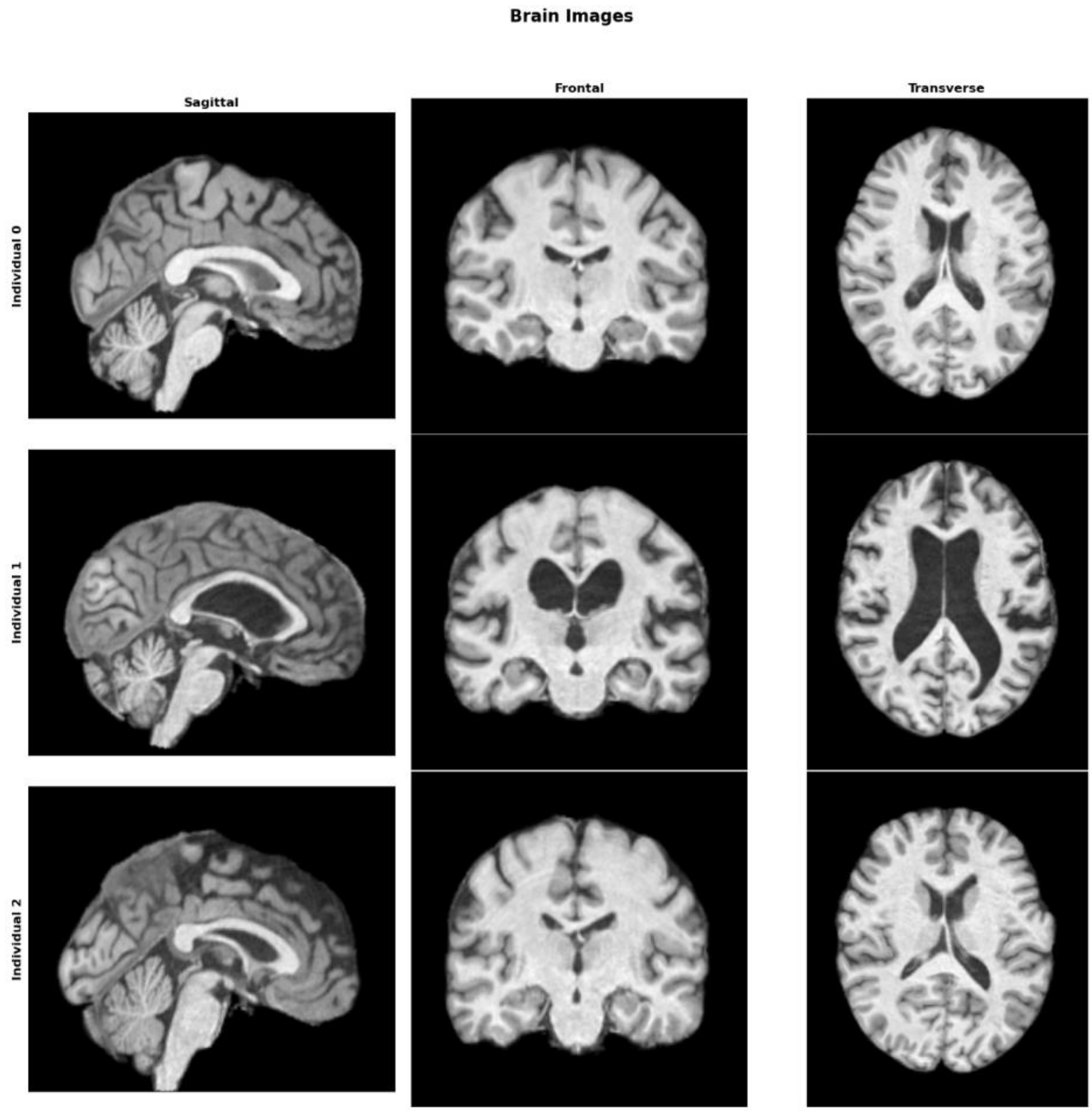


**Supplementary Figure 16 | Extreme outliers caused higher than average reconstruction loss.** Above brain scans show severe anomalies from three individuals potentially due to scan quality, including motion-induced scan disturbance (Individuals 0 and 2), abnormality in brain structure (Individual 1). Notably, sagittal view of scan from Individual 2 shows missing parts in lower section of the image.

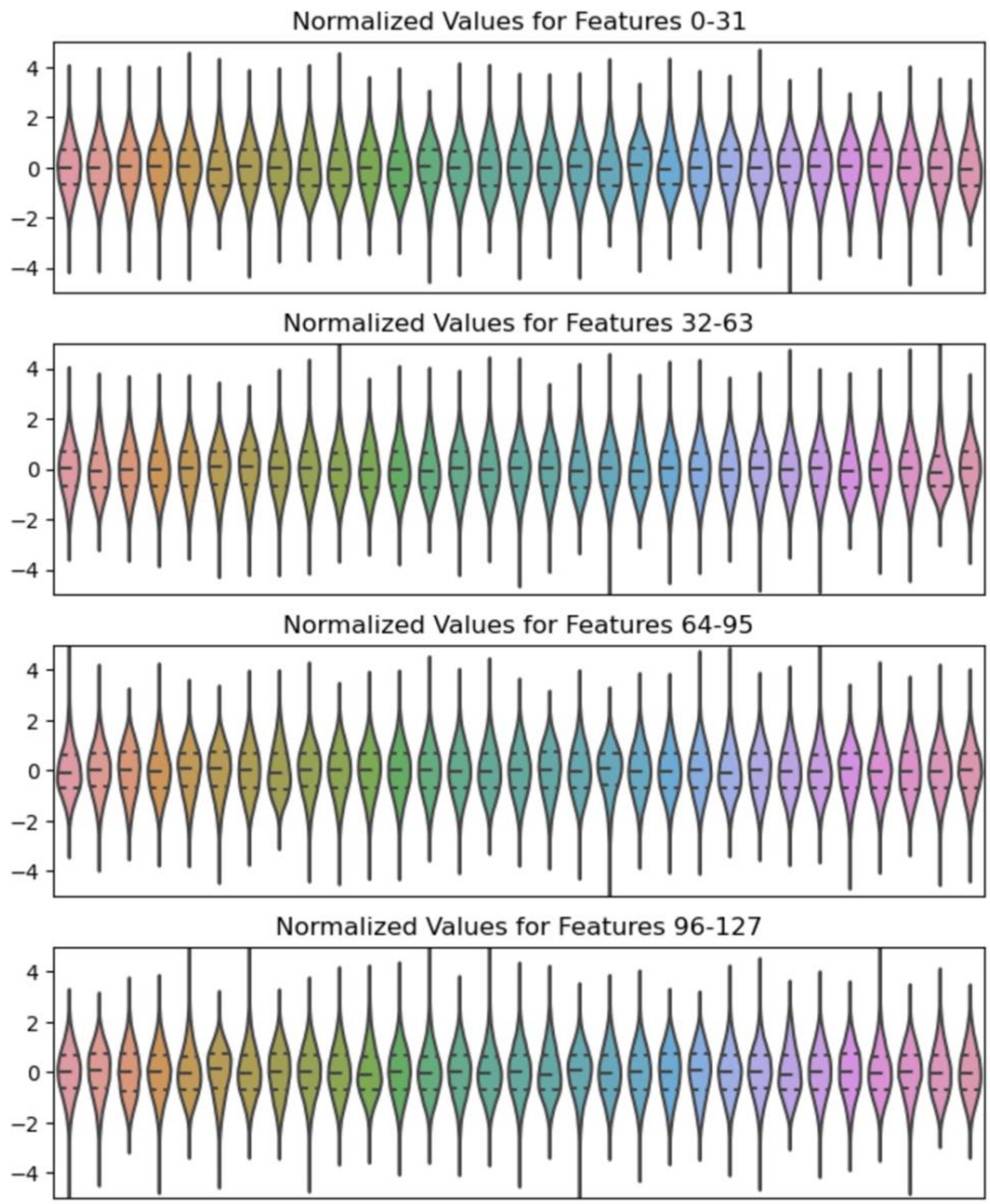


**Supplementary Figure 17 | Visualization of UDIPs showing normal distribution for each of the 128 dimensions upon inferencing (n=22,867).** Violin plots for UDIPs are shown for the normalized distribution of each UDIP for the entire testing dataset. Each plot has three lines: top line for upper quartile (Q3), middle line for median, lower line for lower quartile (Q1). Violin width represents the kernel density estimate (KDE) of the data. The distributions demonstrate overall normality of the UDIPs.

1. Araujo, D. J. *et al.* FoxP1 orchestration of ASD-relevant signaling pathways in the striatum. *Nat. Neurosci.* https://doi.org/10.1101/gad.267989 (2015) doi:10.1101/gad.267989.
2. Bacon, C. *et al.* Brain-specific Foxp1 deletion impairs neuronal development and causes autistic-like behaviour. *Mol. Psychiatry* https://doi.org/10.1038/mp.2014.116 (2015) doi:10.1038/mp.2014.116.
3. Meerschaut, ilse *et al.* FOXP1-related intellectual disability syndrome: a recognisable entity. *J. Med. Genet.* **54**, 613–623 (2017).
4. Grigoryan, T., Wend, P., Klaus, A. & Birchmeier, W. Deciphering the function of canonical Wnt signals in development and disease: conditional loss-and gain-of-function mutations of β-catenin in mice. *Genes Dev.* https://doi.org/10.1101/gad.1686208 (2008) doi:10.1101/gad.1686208.
5. Park, J. H. *et al.* SLC39A8 deficiency: a disorder of manganese transport and glycosylation. *Am. J. Hum. Genet.* https://doi.org/10.1016/j.ajhg.2015.11.003 (2015) doi:10.1016/j.ajhg.2015.11.003.
6. Boycott, K. M. *et al.* Autosomal-recessive intellectual disability with cerebellar atrophy syndrome caused by mutation of the manganese and zinc transporter gene SLC39A8. *Am. J. Hum. Genet.* https://doi.org/10.1016/j.ajhg.2015.11.002 (2015) doi:10.1016/j.ajhg.2015.11.002.
7. Xie, Z. *et al.* Cep120 and TACCs control interkinetic nuclear migration and the neural progenitor pool. *Neuron* **56**, 79–93 (2007).
8. Kodani, A. *et al.* Centriolar satellites assemble centrosomal microcephaly proteins to recruit CDK2 and promote centriole duplication. *Elife* **4**, e07519 (2015).
9. Thurston, G. *et al.* Angiopoietin-1 protects the adult vasculature against plasma leakage. *Nat. Med.* **6**, 460–463 (2000).
