# Supplementary material for: Vision transformer autoencoders captures local and non-local features in brain imaging to reveal novel genetic associations
Source: Commun Biol. 2026 Jun 9;9:1282. doi: 10.1038/s42003-026-10430-6 (PMC13635212; doi:10.1038/s42003-026-10430-6)
Supplement: Supplementary file 2 [file 42003_2026_10430_MOESM3_ESM.pdf]

nature portfolio

Corresponding author(s): Degui Zhi

Last updated by author(s): 2026-2-18

Reporting Summary

Nature Portfolio wishes to improve the reproducibility of the work that we publish. This form provides structure for consistency and transparency in reporting. For further information on Nature Portfolio policies, see our Editorial Policies and the Editorial Policy Checklist.

Statistics

For all statistical analyses, confirm that the following items are present in the figure legend, table legend, main text, or Methods section.

n/a	Confirmed	
☐	☒	The exact sample size (*n*) for each experimental group/condition, given as a discrete number and unit of measurement
☐	☒	A statement on whether measurements were taken from distinct samples or whether the same sample was measured repeatedly
☐	☒	The statistical test(s) used AND whether they are one- or two-sided<br>*Only common tests should be described solely by name; describe more complex techniques in the Methods section.*
☐	☒	A description of all covariates tested
☐	☒	A description of any assumptions or corrections, such as tests of normality and adjustment for multiple comparisons
☐	☒	A full description of the statistical parameters including central tendency (e.g. means) or other basic estimates (e.g. regression coefficient) AND variation (e.g. standard deviation) or associated estimates of uncertainty (e.g. confidence intervals)
☐	☒	For null hypothesis testing, the test statistic (e.g. *F*, *t*, *r*) with confidence intervals, effect sizes, degrees of freedom and *P* value noted<br>*Give P values as exact values whenever suitable.*
☒	☐	For Bayesian analysis, information on the choice of priors and Markov chain Monte Carlo settings
☒	☐	For hierarchical and complex designs, identification of the appropriate level for tests and full reporting of outcomes
☐	☒	Estimates of effect sizes (e.g. Cohen's *d*, Pearson's *r*), indicating how they were calculated

Our web collection on statistics for biologists contains articles on many of the points above.

Software and code

Policy information about availability of computer code

Data collection	Data collections of all using datasets were not performed by us.
Data analysis	• Model: https://github.com/ZhiGroup/UDIP-ViT<br>• FUMA (v.1.5.0): https://fuma.ctglab.nl/, gene mapping, genomic locus annotation<br>• GCTA (v.1.94.1): https://yanglab.westlake.edu.cn/software/gcta/#Overview, fastGWA<br>• LDSC v 1.0.1(https://github.com/bulik/ldsc) to calculate the SNP heritability of UDIPs

For manuscripts utilizing custom algorithms or software that are central to the research but not yet described in published literature, software must be made available to editors and reviewers. We strongly encourage code deposition in a community repository (e.g. GitHub). See the Nature Portfolio guidelines for submitting code & software for further information.

Data

Policy information about availability of data

All manuscripts must include a data availability statement. This statement should provide the following information, where applicable:

- Accession codes, unique identifiers, or web links for publicly available datasets
- A description of any restrictions on data availability
- For clinical datasets or third party data, please ensure that the statement adheres to our policy

The model codes and pre-trained model checkpoints are publicly accessible at Github at https://github.com/ZhiGroup/UDIP-ViT. The UDIPs and GWAS summary statistics are available upon reasonable request after removing sensitive UKbiobank patient information. Genomic loci annotation used data from FUMA (https://fuma.ctglab.nl/). IDP related analysis used traditional IDP list from the Big40 IDP (https://open.oxcin.ox.ac.uk/ukbiobank/big40/). Individual data from UKBB can be requested with proper registration at https://www.ukbiobank.ac.uk/. All unrestricted data supporting the findings are also available from the corresponding author upon request.

Research involving human participants, their data, or biological material

Policy information about studies with human participants or human data. See also policy information about sex, gender (identity/presentation), and sexual orientation and race, ethnicity and racism.

Reporting on sex and gender	The term sex (biological attribute, i.e., female or male) was used in this study.
Reporting on race, ethnicity, or other socially relevant groupings	n/a
Population characteristics	Our analysis used genomes, brain MR image as major analysis target, and age, body weight, height, waist circumference, BMI as covariates
Recruitment	Data collections of all using datasets were not performed by us. Please refer to the UKbiobank website https://www.ukbiobank.ac.uk/
Ethics oversight	Our analysis was approved by the UTHealth Houston committee for the protection of human subjects under No. HSC-SBMI-20-1323. UKBB has secured informed consent from the participants in the use of their data for approved research projects. UKBB data was accessed via approved project 24247.

Note that full information on the approval of the study protocol must also be provided in the manuscript.

Field-specific reporting

Please select the one below that is the best fit for your research. If you are not sure, read the appropriate sections before making your selection.

☒ Life sciences ☐ Behavioural & social sciences ☐ Ecological, evolutionary & environmental sciences

For a reference copy of the document with all sections, see nature.com/documents/nr-reporting-summary-flat.pdf

Life sciences study design

All studies must disclose on these points even when the disclosure is negative.

Sample size	In total, 28,997 samples were included in this study. Model development used 4,597 samples for training and 1,533 for validation. Learned representations were subsequently extracted from the remaining 22,867 samples, corresponding to the same “discovery cohort” used in prior brain imaging GWAS analyses.
Data exclusions	UKBB participants that do not have T1 weighted and T2 weighted FLAIR MR image data were excluded.
Replication	No replication was conducted given the huge sample size of the discovery cohort for the GWAS analysis. Additionally, there are only a limited number of samples remaining for the replication study, while GWAS analyses require large sample sizes to achieve robust and statistically significant results. In the future, we could perform a replication analysis using additional data from upcoming UK Biobank releases.
Randomization	The training and validation study split is random with a specific seed. The 22,867 sample used for representation extraction and GWAS analysis is using the same cohort as previous GWAS analyses.
Blinding	The selection of 28,997 samples is blind to us.

Reporting for specific materials, systems and methods

We require information from authors about some types of materials, experimental systems and methods used in many studies. Here, indicate whether each material, system or method listed is relevant to your study. If you are not sure if a list item applies to your research, read the appropriate section before selecting a response.

Materials & experimental systems

n/a	Involved in the study
☒	☐ Antibodies
☒	☐ Eukaryotic cell lines
☒	☐ Palaeontology and archaeology
☒	☐ Animals and other organisms
☒	☐ Clinical data
☒	☐ Dual use research of concern
☒	☐ Plants

Methods

n/a	Involved in the study
☒	☐ ChIP-seq
☒	☐ Flow cytometry
☐	☒ MRI-based neuroimaging

Plants

Seed stocks: n/a

Novel plant genotypes: n/a

Authentication: n/a

Magnetic resonance imaging

Experimental design

Design type: n/a

Design specifications: n/a

Behavioral performance measures: n/a

Acquisition

Imaging type(s): T1 weighted and T2 weighted FLAIR images from UKbiobank

Field strength: 3 Tesla

Sequence & imaging parameters: MRI images were acquired using standard Siemens Skyra 3T running VD13A SP4 (as of October 2015), with a standard Siemens 32-channel RF receive head coil. Resolution of T1 is 1×1×1 mm, and resolution of T2 is 1.05×1×1mm (https://biobank.ctsu.ox.ac.uk/crystal/crystal/docs/brain_mri.pdf).

Area of acquisition: whole brain

Diffusion MRI: ☐ Used ☒ Not used

Preprocessing

Preprocessing software: To promote generalizability and minimize manual feature engineering, we adopted the standard preprocessing pipeline developed by the UK Biobank MRI team. Brain MRI preprocessing was performed primarily using FSL (https://www.fmrib.ox.ac.uk/ukbiobank/), and included defacing, brain extraction with BET, linear and non-linear registration to standard space using FLIRT and FNIRT, and bias-field correction using FAST. Bias-field-corrected, brain-extracted T1-weighted and T2-weighted FLAIR images were retained for analysis. All images were subsequently linearly registered to MNI152 space using affine transformation with 12 degrees of freedom via UK Biobank-provided precomputed matrices in FLIRT. Linear registration was chosen to normalize head size and achieve cross-subject alignment while preserving subject-specific structural deformations, in contrast to non-linear registration. The resulting linearly registered, defaced, bias-field-corrected images were used in all downstream analyses. Image intensities were normalized on a per-scan basis using Z-score normalization. Following affine registration, T1- and T2-weighted volumes had dimensions of 182 × 218 × 182 voxels and were zero-padded to 182 × 224 × 182 to enable partitioning into equal-sized patches.

Normalization	linear registration to MNI space.
Normalization template	MNI space
Noise and artifact removal	n/a
Volume censoring	n/a

Statistical modeling & inference

Model type and settings	n/a
Effect(s) tested	n/a

Specify type of analysis: ☒ Whole brain ☐ ROI-based ☐ Both

Statistic type for inference (See Eklund et al. 2016)	n/a
Correction	n/a

Models & analysis

n/a	Involved in the study
☒	☐ Functional and/or effective connectivity
☒	☐ Graph analysis
☐	☒ Multivariate modeling or predictive analysis

Multivariate modeling and predictive analysis	We adopted a deep learning model for extracting the representation of T1 weighted and T2 weighted FLAIR images. Details show in Methods.
