# Supplementary material for: Vision transformer autoencoders captures local and non-local features in brain imaging to reveal novel genetic associations
Source: Commun Biol. 2026 Jun 9;9:1282. doi: 10.1038/s42003-026-10430-6 (PMC13635212; doi:10.1038/s42003-026-10430-6)
Supplement: Supplementary file 3 [file 42003_2026_10430_MOESM4_ESM.pdf]

nature portfolio

Peer Review File

Vision Transformer Autoencoders Captures Local and Non-Local Features in Brain Imaging to Reveal Novel Genetic Associations

Corresponding Author: Professor Degui Zhi

This file contains all reviewer reports in order by version, followed by all author rebuttals in order by version.

Version 0:

Reviewer comments:

Reviewer #1

(Remarks to the Author)
The authors explored the potential of using unsupervised learning derived image phenotypes for discovering genetic associations, in UK Biobank structural brain image and genetic data. This is a timely endeavor and an interesting approach, which has the potential to overcome limitations of traditional approaches based on e.g. atlas-defined brain regions.

I do not have expertise in unsupervised learning applied to neuroimage data, but I can comment from the perspective of genetic analysis applied to brain imaging.

From the introduction: 'Previous works on brain imaging GWAS have primarily focused on image-derived phenotypes (IDPs) via image processing pipelines (Elliott et al., 2018; Smith et al., 2021). However, IDPs can miss subtle or complex patterns due to their reliance on predefined features and model assumptions ...'. I think this description does not capture the extent to which data-driven IDPs have been used in genome-wide association studied (GWAS), such as derived from independent component analysis of resting functional data. If the authors could explicitly rephrase this part around brain structure GWAS, rather than brain imaging GWAS more generally, then it would be more accurate.

In the author's GWAS, head size was used as a confound covariate. Head size is a heritable measure, and using heritable measures as covariates in GWAS can cause bias (Aschard et al. 2015 https://www.sciencedirect.com/science/article/pii/S0002929714005278?via=ihub), including spurious associations driven by the covariate adjustment. It would be advisable to run GWAS also without head size adjustment and compare the results.

I found some aspects of the sample splitting to be unclear. From the Methods: '6,130 MRI scans were chosen for the model and split into 75/25 training/validation sets. 4,597 scans were used for training, and 1,533 scans were used for validation. The dataset used for GWAS consisted of 37,376 scans, of which we selected 22,867 as discovery cohorts for later GWAS analysis.' Then in the Discussion 'As an experimental approach, we trained our ViT-AE model using the combined original training and testing datasets and validated it on a disjoint validation set ... Although the trained model identified additional associated loci, these findings were excluded from the main results because the GWAS analysis was conducted on the original testing dataset, which was included in the training process ... Nonetheless, we present these findings to illustrate the ViT-AE model's potential to perform better with a larger training dataset.' These aspects are difficult for the reader to follow, so please clarify with exact details on dividing the sample for which purposes and explaining the rationale in the Methods section, so that the reader can understand which results may have been affected by exaggerated model performance. A schematic figure might help.

For overlap analysis, the authors cross-referenced their GWAS hits that had Bonferroni-corrected significance $p< 5\times10^{-8}/128$ against previously reported GWAS hits from Smith et al. 2021 that had Bonferroni-corrected significance $p< 5\times10^{-8}/1437$. The Bonferroni correction was more severe for Smith's findings, because Smith et al., ran GWAS with a greater number of brain structural measures. However, a consequence is that the GWAS hits from Smith et al. would need to be roughly one order of magnitude more significant than those in the present study, before Bonferroni adjustment. Then, the Results text (and e.g. the Fig 3 legend, the Table 1 contents, and the abstract) focuses on comparing GWAS results from the present study with an earlier study by Patel et al. (2024) who used a convolutional neural network (CNN) approach for

deriving brain structural measures for GWAS. The reported numbers of novel genetic loci in the present study are made with reference to Patel et al., rather than Smith et al.. This leaves the question of how many of the genetic loci were truly novel, and this appears to be answered at the start of the discussion, with reference to the GWAS catalog. I understand that the overlap with Smith et al. was used as a metric to asses model performance, but this creates a confusing aspect to the paper where the description of the Results does not follow clearly from the overlap analysis in the Methods. This leaves readers wondering how many of the genetic loci were truly novel in the present study. Please clarify throughout the paper what is meant by 'new' or 'novel' loci, with respect to Smith et al., Patel et al. and GWAS catalog.

From the Results: 'Out of the 24 loci newly identified by our method, 14 loci show at least one of their top five associated traits related to brain structure or function.' Does this refer to the GWAS catalog? Please clarify.

From the Results: 'In contrast, 10 loci have not been previously reported to associate with any of the top five traits related to brain structure or function.' Does this mean that none of the top 5 traits in the GWAS catalog for any of these 10 loci was related to brain structure or function? Please clarify.

In the Results and Discussion, the authors claim that bone mineral density could indirectly influence gray matter changes in the brain. Please also acknowledge the possibility of horizontal pleiotropy, when a single genetic variant independently influences distinct traits, rather than affecting one trait through another.

In the Discussion, the authors report that their model gives a disproportionate amount of attention to 'anatomically informative regions with high structural variance, such as the boundaries between different tissue types (e.g., gray and white matter), the edges of ventricles, or the complex folding of deep sulci.' This seems to be both a potential strength and also weakness of the approach. A strength because it confirms that the model is sensitive to the most overt features of the image data, so is working broadly as expected. A weakness because more subtle aspects of brain structure are overlooked. The Discussion could be more nuanced on this point.

In Table 1 and supplementary Table 4 please include information on minor allele frequency and association effect size.

I find a section heading from the Results difficult to follow: 'Novel loci lead SNPs gene discovered associated with brain structure'.

Reviewer #2

(Remarks to the Author)
In this paper, Islam et al. described a framework that applies a Vision Transformer (ViT)-based autoencoder to derive 128-dimensional representations from brain MRI scans of UK Biobank participants. These vectors are named unsupervised learning derived image phenotypes from ViT (ViT-UDIP). Then Islam et al. conducted GWAS on these UDIP using UK Biobank data and identified 63 loci. Their findings demonstrate the value of transformer-based architectures in discovering novel and robust imaging phenotypes for genetic discovery.

The UDIP idea is very interesting and an attractive alternative to IDP. Overall, the paper is well written and is of high quality.

But an unsettling issue for me is whether these UDIPs are reproducible. I mean, if one repeats the same procedure on a different training dataset, whether the exact same set of UDIP will be derived and in the same order? I doubt this since the ViT or NN are complex models and dynamic. Therefore, how to match these UDIPs could be a challenging problem.

In the introduction section, some of the literature reviews are inaccurate. For example, the authors wrote that "Yu et al. demonstrated… The primary challenge with this approach is that labor-intensive labeled data for supervised deep learning carry the same biases from the human labeler." This interpretation is incorrect. Labels used in Yu et al. are SNP genotypes, which are readily available after genotyping. No labeler is involved so there is no bias.

Heritability. Please add some details in the Methods section on how the heritability is calculated.

I found it interesting that for the 10 loci have not been previously reported to associate with any of the top five traits related to brain structure or function. The most commonly reported non-brain-related traits include bone mineral density, height, body mass index (BMI), body fat distribution, and cardiovascular-related traits such as carpal tunnel syndrome, as well as some lung function and
colorectal disease measures. The authors reasoned that these traits are more or less age-related making them reasonable. But I think potential rationale is that these traits are related to body or tissue morphology and development, which may also affect the shape and size of the brain.

For the GWAS results, the authors claims that 24 genetic loci out of 63 found to be associated with brain structure by the ViT approach were missed by a previous CNN-AE-based approach" and considered this a success. While I agree ViT is perhaps a more powerful method than CNN, but this claim is a bit misleading. I wonder, conversely, whether there is any locus that is identified by CNN and is missed by ViT. The two methods share some common findings as well as some unique findings, this alone doesn't make one method superior to the other. Unless the new method identified all the loci found before and additionally, it found 24 new ones and all the new loci are biologically meaningful. Some clarification is warranted on this point in my opinion.

Reviewer #3

(Remarks to the Author)
The paper proposes using Visual Transformers to generate a new class of deep learning image-derived phenotypes (IDP), which the authors have termed 'ViT-UIDP'. These phenotypes are then studied using Genome-Wide Association Studies (GWAS) in the UK Biobank population cohort. Interesting original loci of association are derived and annotated. The ViT-UIDP phenotype class is then compared to another DL-derived IDP class that the authors of the paper previously developed (CNN-UIDP).

This contribution is well organised and follows a classic structure. The paper reports on the genetic association of the embeddings learnt by an unsupervised model equipped with attention modules on local/non-local features which is of clear interest for the brain mapping community.
The papers is proposed with companion code. I did not test the repository but I parsed it and checked that it follows the state of the art (requirements.txt, example entry, lean source entry - with classical organization using torch only as deep-learning module dependency).

Yet, I have a set of major issues to be addressed in the paper.

1. Introduction
The progression of ideas in this paragraph is unclear. It should be reorganized and expanded. I suggest organizing the ideas more clearly. 1) Starting from the existing text, the authors could begin with a general statement about brain structure and genetics (justify why you selected these citations – there are many more!), 2) The use hand-crafted phenotypes that were historically developed in small cohorts using image processing (cite classical features dedicated to cortex, grey matter, white matter and structural and functional connectivity), 3) The move to data-driven phenotype extraction using supervised and unsupervised methods. More references should be given beyond (Patel 2024), such as DOIs listed at thereafter. Introduce ViT as an alternative to CNN. 5) Introduce PerDI (citing Patel) and also the concept of spotlighting. Finally, introduce the main results annotation: yielding loci with annotations and yielding dimension with PerDI annotations.
I suggest to introduce the work on the specificity of the ViT architecture investigated thanks to SpotLight token which would justify the local and non-local words of the title.
I think it is important to introduce better all the points of the paper with really more citations for all the points of the introduction.
Proposed citations for embedding and genetics
https://doi.org/10.1371/journal.pgen.1011332 https://direct.mit.edu/imag/article/doi/10.1162/IMAG.a.987/133600

2. Methods
2.1 Data selection: I do not share the authors' opinion about the potential for information leakage if the same data were used to: i) train the ViT model, and ii) conduct the GWA study on the embeddings. Instead, I think the same subjects could have been considered, bearing in mind that genetics is never used in training the ViT model. It is a pity, since, as the authors note, a larger training set would improve the quality of the training and association findings (see the remarks in the Results section). I would like another argument to be given for this choice of split, as I believe it is misleading.

2.2 Why so many covariables were chosen: are there specific reasons related to the choice of the architecture and training? Justification could again be given based on citations. Alternatively, the effect size and significance could be given for variables such as “date of attending”, “scanner table position”. On the contrary why only 10 genetic PC are kept and not the 40 ones available?

2.3 minP is used to perform a conservative multivariate analysis. Could the procedure be detailed a little bit in the main manuscript: do you use permutation or simply take the min across all the features summary-statistics?

2.4 The sparse attention pattern. It I unclear to me if the sparse attention pattern is a consequence of the choice by the authors of the \sigma_{r} version of the matrix \sigma (see the explanation given in Supplementary Note 3. If this is true, I think some information should be given in the main text in the Method part.

3. Results
3.1 Figure 3: A Miami plot could be given to relate/contrast the ViT and CNN UIDPs

3.2 In the paragraph starting with: “Out of the 24 loci…” and “In contrast, 10 loci….”, The authors mention “brain structure or function” annotation. Could you give more details about the generation of these annotations and their source? Is it an output from FUMA? As far as I know this is not direct output and human processing must be performed: could it be mentioned in the main text.

3.3 Regarding the 3 next paragraphs “Mutiple genes in …”, “TCF7LI, a transcription factor…” and “Beyond neuronal and

metabolic…”. I think they are unduly detailed. They reflect the FUMA output which could be reported in the Supplemental data and referred. Finally, the conclusions of these (probably LLM generated) text is not supported by any data/results in the paper (they may be not false but they are not supported by reported elements). Could this part be truncated and left with only the very succinct report of what those gene are known to be linked with (experimentally verified). Also the sentence “Find table below” looks strange?

3.4 Spotlight investigation. Figure 5 is difficult for non-specialists to understand, especially the legend for part 5C. The bipartite graph should also be clarified with regard to the start and end points of the arrows. More generally, this section would be clearer if a counterpart paragraph were included in the 'Methods' section.
The paragraph “We found that this behavior arises from the specific embeddings learned by the model (see Supplementary Note 3). The ….” should be clarified: the notion of “similar direction and weak correlation” should be really reworked.
The same with the paragraph “We then visualize the spatial probability of spotlight tokens across brain slices for models trained with different parameter settings and random seeds. As shown in Figure 5c, red regions highlight anatomical locations where the model consistently concentrates its attention across […] These results indicate strong robustness and stability of the identified spotlight tokens.” The concepts of 'masked brain' patches and the discussion of 'high and low correlations' refer to contextual information that should be explained here. The experimental setup used to produce these numbers (18% 0.75 0.92..) should also be described in detail.

3.5 Suppl figure 3. The legend for the colors appearing in the figure should be given.

Version 2:

Reviewer comments:

Reviewer #1

(Remarks to the Author)
Thank you for addressing my earlier points with additional information and clarification.

I am still somewhat confused by the dataset splitting.
From the ‘Methods’: ‘The dataset used for GWAS consisted of 37,376 scans, of which we selected 22,867 as discovery cohorts for later GWAS analysis.’ Why is ‘cohorts’ here plural? Why not use the whole 37,376 for GWAS? Were the remaining 14,509 (i.e. 37,376-22,867) used for anything in this study? Or did the 14,509 not meet quality control in the genetic pipeline? On what basis were the 22,867 ‘selected’? Figure 1 mentions 37,376 as the testing set.

I think that these two revised sentences in the introduction need to be corrected:
‘Early genome-wide association studies (GWAS) have focused on direct phenotype measurements (Brouwer et al., 2022; Garcia-Marin et al., 2024; Hibar et al., 2015, 2017; Stein et al., 2012) or statistical and mathematical representations of anatomical modeling and population genetics (Brun et al., 2009; Hulshoff Pol et al., 2006). Recent works on brain structure GWAS have shifted to image-derived phenotypes (IDPs), driven by the availability of large-scale imaging datasets and processing pipelines. (Elliott et al., 2018; Smith et al., 2021; Zhao et al., 2019).’
- In the first of these sentences, referring to studies from 2022 (Brouwer et al.) and 2024 (Garcia-Marin et al.) as ‘early’ genome-wide association studies does not make much sense, as they were only published 2-4 years ago.
- In the second of these sentences, referring to how recent studies have ‘shifted’ to IDPs does not make much sense either. The ‘recent’ studies cited here are older than two of the ‘early’ studies in the sentence before. More importantly, many of the ‘early’ studies cited in the first sentence used IDPs as well. Maybe they did not call their brain measures IDPs, but they were typically from automated processing pipelines in large datasets. Therefore, the distinction made by the authors between ‘direct phenotype measurements’ in older studies and ‘IDPs’ in newer studies is not really valid. I think it should be possible for the authors to describe the novelty of their approach while still representing the previous literature correctly.

Reviewer #2

(Remarks to the Author)
The authors have addressed most of my concerns. I noticed that the authors removed the mention of Yu et al. I am a bit disappointed that the authors seem unwilling to follow my review advice. I think this paper should be cited and discussed since the topic of that paper is closely related to the present study.

Reviewer #3

(Remarks to the Author)
The authors answered my questions adequately and rephrased their text when requested. In my opinion, they did so correctly with regard to the Editor's and other reviewers'.

I have two remarks, for which I apologise as I did not raise them in my first review. I am confident that the authors will respond without my intervention.
-The selection of 22,867 out of 37,776 subjects is neither justified nor explained anywhere. Could this be explained?

-Regular practice when performing a GWAS is to consider a sample from a homogeneous population (even if population structure is controlled with the ten PCs in the model). Did the authors only retain subjects with British ancestry (field 21000)? If so, this could be mentioned.

Open Access This Peer Review File is licensed under a Creative Commons Attribution 4.0 International License, which permits use, sharing, adaptation, distribution and reproduction in any medium or format, as long as you give appropriate credit to the original author(s) and the source, provide a link to the Creative Commons license, and indicate if changes were made.
In cases where reviewers are anonymous, credit should be given to 'Anonymous Referee' and the source.
The images or other third party material in this Peer Review File are included in the article's Creative Commons license, unless indicated otherwise in a credit line to the material. If material is not included in the article's Creative Commons license and your intended use is not permitted by statutory regulation or exceeds the permitted use, you will need to obtain permission directly from the copyright holder.
To view a copy of this license, visit https://creativecommons.org/licenses/by/4.0/

“Vision Transformer Autoencoders for Unsupervised Representation Learning: Capturing Local and Non-Local Features in Brain Imaging to Reveal Genetic Associations” Response Letter

Editor:

We hope you will find the referees' comments useful as you decide how to proceed. While we would generally ask that you address all discussion points, methodological clarifications, and analytical concerns raised by the reviewers, it would be particularly important to:

(1) Evaluate the reproducibility of these UDIPs (per Reviewer #2)

We thank the reviewer for bringing up this comment. Individual UDIP dimensions will change when using different training datasets or even different random seeds. However, the overall multivariate distribution of UDIPs, as well as the loci discovered based on them, remains largely consistent. To demonstrate the reproducibility of these UDIPs, we trained the network under three sources of variability: different training seeds, different data splits, and distinct datasets (ADNI) with varying patient backgrounds. The results show strong alignment in UDIP distributions (as measured by CKA and CCA) and a high degree of overlap in the identified loci. Specifically, CKA (range 0 to 1) achieves an average of 0.83 across different training seeds, 0.80 across different data splits, and 0.60 across distinct datasets. CCA between UDIPs shows strong alignment, with correlations exceeding 0.8 for the first 30 canonical components, indicating that these components can be linearly transformed to closely match one another and are therefore effectively interchangeable. Moreover, the explained variance of one UDIP by another averages around 0.7, where explained variance is defined as the fraction of the total variance in X that is shared with Y. The overlap analysis shows an average of 37 loci for different training seeds, 35 for different data splits, and 17 for distinct datasets. These findings have been added to Methods (Line112-117), Results (Line 236-244), Discussion (Line 416-425), Supplementary Note 5, and Supplementary Figure 13-15.

(2) Justify the broad selection of covariates (per Reviewers #1 and #3), including Reviewer #1’s concern about the reliance on head size

We thank the reviewer for this comment. We follow the same list of covariates from Patel et al 2024. To be more specific, this list includes GWAS covariates following the prior Elliott et al. 2018 UK Biobank imaging GWAS study and Smith et al. 2021 UK Biobank imaging genetics study, taking the union of covariates used in both studies. As for the head size covariate, many existing well-known papers include head size as the confounding covariate for the GWAS analysis, including Elliott et al., 2018, The MULTI Consortium, Boquet-Pujadas et al, 2025. To further answer the reviewer’s question, we conduct the GWAS analysis without the head size covariate. The resulting loci and leadSNP remain unchanged (data not shown).

(3) Clarify how novel variants were determined (Reviewer #1) and whether any of these are more broadly relevant to body or tissue morphology (Reviewer #2)

We thank the reviewer for this valuable suggestion. We have updated the Methods section to include a session of “define novel loci”. Among the identified novel loci, 10 out of 24 appear to be broadly related to body or tissue morphology. Although these loci are not previously associated with brain-related traits, many map to genes involved in fundamental developmental pathways, such as Wnt and TGF-β signaling. These pathways regulate cell proliferation, differentiation, and tissue patterning across multiple organ systems. As a result, associations with traits such as bone mineral density, height, and body composition may reflect shared biological mechanisms governing overall morphology, including brain structure. For example: Locus 11 (lead SNP: rs34644194, chr2:50Mb, mapped gene: TCF7L1) is associated with heel bone mineral density and blood pressure. TCF7L1 regulates Wnt signaling, a pathway critical for early brain development and cortical expansion.

Locus 56 (lead SNP: rs4444235, chr14:20Mb, mapped gene: BMP4) has been associated with heel bone mineral density, lung function (FEV1/FVC), and colorectal disease. BMP4 is a key morphogen in both skeletal and neural development, influencing cortical patterning and neural progenitor

differentiation. These examples illustrate that variants influencing "non-brain" traits may also impact brain morphology, likely through shared developmental and tissue growth programs rather than brain-specific processes. Details of other loci could be found in the Supplementary Table 5. We have included the entire rationale in the updated manuscript (Line290-295).

(4) Justify the data selection (Reviewer #3; point 2.1). While we recognize that paired neuroimaging and genetic data are sparse, we would strongly encourage you to include additional validation in other cohorts where possible, or at the very least better acknowledge the limitations of this approach and the reliance on solely UKBB data.

We thank the reviewer for raising this point. We acknowledge the limitation of this approach for reliance on the UKBB data and for Reviewer #3; point 2.1. We also admit our approach might be over-conservative for strictly separate GWAS and training cohorts, but we prioritized maintaining a clean and reliable evaluation of model performance. We include this in the Discussion (Line449-451).

Reviewer#1:

The authors explored the potential of using unsupervised learning derived image phenotypes for discovering genetic associations, in UK Biobank structural brain image and genetic data. This is a timely endeavor and an interesting approach, which has the potential to overcome limitations of traditional approaches based on e.g. atlas-defined brain regions.

I do not have expertise in unsupervised learning applied to neuroimage data, but I can comment from the perspective of genetic analysis applied to brain imaging.

From the introduction: 'Previous works on brain imaging GWAS have primarily focused on image-derived phenotypes (IDPs) via image processing pipelines (Elliott et al., 2018; Smith et al., 2021). However, IDPs can miss subtle or complex patterns due to their reliance on predefined features and model assumptions ...'. I think this description does not capture the extent to which data-driven IDPs have been used in genome-wide association studied (GWAS), such as derived from independent component analysis of resting functional data. If the authors could explicitly rephrase this part around brain structure GWAS, rather than brain imaging GWAS more generally, then it would be more accurate.

We thank the reviewer for bringing this to our attention. We have rephrased accordingly.

In the author's GWAS, head size was used as a confound covariate. Head size is a heritable measure, and using heritable measures as covariates in GWAS can cause bias (Aschard et al. 2015 https://www.sciencedirect.com/science/article/pii/S0002929714005278?via=ihub), including spurious associations driven by the covariate adjustment. It would be advisable to run GWAS also without head size adjustment and compare the results.

We thank the reviewer for this comment. We follow the same list of covariates from Patel et al 2024. To be more specific, this list includes GWAS covariates following the prior Elliott et al. 2018 UK Biobank imaging GWAS study and Smith et al. 2021 UK Biobank imaging genetics study, taking the union of covariates used in both studies. As for the head size covariate, many existing well-known papers include head size as the confounding covariate for the GWAS analysis, including Elliott et al., 2018, The MULTI Consortium, Boquet-Pujadas et al, 2025. To further answer the reviewer's question, we conduct the GWAS analysis without the head size covariate. The resulting loci and leadSNP remain unchanged.

I found some aspects of the sample splitting to be unclear. From the Methods: '6,130 MRI scans were chosen for the model and split into 75/25 training/validation sets. 4,597 scans were used for training, and 1,533 scans were used for validation. The dataset used for GWAS consisted of 37,376 scans, of which we selected 22,867 as discovery cohorts for later GWAS analysis.' Then in the Discussion 'As an experimental approach, we trained our ViT-AE model using the combined original

training and testing datasets and validated it on a disjoint validation set ... Although the trained model identified additional associated loci, these findings were excluded from the main results because the GWAS analysis was conducted on the original testing dataset, which was included in the training process ... Nonetheless, we present these findings to illustrate the ViT-AE model's potential to perform better with a larger training dataset.' These aspects are difficult for the reader to follow, so please clarify with exact details on dividing the sample for which purposes and explaining the rationale in the Methods section, so that the reader can understand which results may have been affected by exaggerated model performance. A schematic figure might help.

We thank the reviewer for bringing this up. The original data split was designed to match the "discovery cohort" setup used in Elliott et al. 2018 UK Biobank imaging GWAS study and Smith et al. 2021 UK Biobank imaging genetics study, where a large portion of the UK Biobank samples is reserved for GWAS, leaving only a few thousand samples for model training. However, ViTs, as transformer-based architectures, are known to be data-hungry. We therefore expect that increasing the training set size would improve performance. Motivated by this, we conducted an additional experiment in which the "test data" (replication cohort), originally reserved solely for GWAS, is also incorporated into the training set.

We have modified the discussion to make it clearer (Line443-451).

For overlap analysis, the authors cross-referenced their GWAS hits that had Bonferroni-corrected significance p< 5×10-8/128 against previously reported GWAS hits from Smith et al. 2021 that had Bonferroni-corrected significance p< 5×10-8/1437. The Bonferroni correction was more severe for Smith's findings, because Smith et al., ran GWAS with a greater number of brain structural measures. However, a consequence is that the GWAS hits from Smith et al. would need to be roughly one order of magnitude more significant than those in the present study, before Bonferroni adjustment. Then, the Results text (and e.g. the Fig 3 legend, the Table 1 contents, and the abstract) focuses on comparing GWAS results from the present study with an earlier study by Patel et al. (2024) who used a convolutional neural network (CNN) approach for deriving brain structural measures for GWAS. The reported numbers of novel genetic loci in the present study are made with reference to Patel et al., rather than Smith et al.. This leaves the question of how many of the genetic loci were truly novel, and this appears to be answered at the start of the discussion, with reference to the GWAS catalog. I understand that the overlap with Smith et al. was used as a metric to asses model performance, but this creates a confusing aspect to the paper where the description of the Results does not follow clearly from the overlap analysis in the Methods. This leaves readers wondering how many of the genetic loci were truly novel in the present study. Please clarify throughout the paper what is meant by 'new' or 'novel' loci, with respect to Smith et al., Patel et al. and GWAS catalog.

We thank the reviewer for this valuable suggestion. We report loci overlap of ViT-UDIPs with both Smith et al. 2021 UK Biobank imaging genetics study and Patel et al. imaging genetics study in the newly added Supplementary Figure 12 and Supplementary Table 6. Twenty loci from ViT-UDIPs remain novel after loci overlapping analysis with both CNN-UDIPs and IDPs. We have updated the Methods section to include a session of "define novel loci", as well as clarified the meaning of 'new' or 'novel' loci within the Results section, such that the reader should not get confused (Line 254-256).

For the GWAS Catalog comparison, which aggregates loci reported across a large body of literature, we provide a qualitative assessment by highlighting the studies with the five most significant (lowest p-value) associations (Line 276-296)

From the Results: 'Out of the 24 loci newly identified by our method, 14 loci show at least one of their top five associated traits related to brain structure or function.' Does this refer to the GWAS catalog? Please clarify.

We are sorry for not making the statement clear. Yes, this refers to the GWAS catalog. We have modified the related Results session to directly use the word 'GWAS catalog' (Line272-296).

From the Results: 'In contrast, 10 loci have not been previously reported to associate with any of the top five traits related to brain structure or function.' Does this mean that none of the top 5 traits in the GWAS catalog for any of these 10 loci was related to brain structure or function? Please clarify.

Yes, the reviewer understands it correctly. We have clarified it in the Line 286-287.

In the Results and Discussion, the authors claim that bone mineral density could indirectly influence gray matter changes in the brain. Please also acknowledge the possibility of horizontal pleiotropy, when a single genetic variant independently influences distinct traits, rather than affecting one trait through another.

We thank the reviewer for bringing this up. We have updated both parts to include this possibility (Line 350-351, Line 489-491).

In the Discussion, the authors report that their model gives a disproportionate amount of attention to 'anatomically informative regions with high structural variance, such as the boundaries between different tissue types (e.g., gray and white matter), the edges of ventricles, or the complex folding of deep sulci.' This seems to be both a potential strength and also weakness of the approach. A strength because it confirms that the model is sensitive to the most overt features of the image data, so is working broadly as expected. A weakness because more subtle aspects of brain structure are overlooked. The Discussion could be more nuanced on this point.

We thank the reviewer for raising this point. The subtle aspects refer to the other regions of the brain that do not receive prominent attention weights. We want to emphasize that attention patterns are learned, and are more than just the most prominent ones that we highlighted. There are more subtle changes - these are also captured by the model's attention mechanisms - but are less interpretable, and thus not highlighted. We have included a nuanced interpretation in the discussion section (Line 436-439).

In Table 1 and supplementary Table 4 please include information on minor allele frequency and association effect size.

We have included the minor allele frequency (MAF) and association effect size (BETA) for the leadSNP within loci in the Table 1 and Supplementary Table 4.

I find a section heading from the Results difficult to follow: 'Novel loci lead SNPs gene discovered associated with brain structure'.

We have changed the section heading to be 'Novel loci discovered associated with brain structure'.

Reviewer#2:

In this paper, Islam et al. described a framework that applies a Vision Transformer (ViT)-based autoencoder to derive 128-dimensional representations from brain MRI scans of UK Biobank participants. These vectors are named unsupervised learning derived image phenotypes from ViT (ViT-UDIP). Then Islam et al. conducted GWAS on these UDIP using UK Biobank data and identified 63 loci. Their findings demonstrate the value of transformer-based architectures in discovering novel and robust imaging phenotypes for genetic discovery.

The UDIP idea is very interesting and an attractive alternative to IDP. Overall, the paper is well written and is of high quality.

But an unsettling issue for me is whether these UDIPs are reproducible. I mean, if one repeats the same procedure on a different training dataset, whether the exact same set of UDIP will be derived and in the same order? I doubt this since the ViT or NN are complex models and dynamic. Therefore, how to match these UDIPs could be a challenging problem.

We thank the reviewer for pointing this out. Individual UDIP dimensions will change when using different training datasets or even different random seeds. However, the overall multivariate

distribution of UDIPs, as well as the loci discovered based on them, remains largely consistent. To support this, we performed CKA, CCA analyses, and loci-overlap experiments across UDIPs derived from different training seeds and training datasets (See Supplementary Note 5 and Supplementary Figure 13-15).

In the introduction section, some of the literature reviews are inaccurate. For example, the authors wrote that "Yu et al. demonstrated… The primary challenge with this approach is that labor-intensive labeled data for supervised deep learning carry the same biases from the human labeler." This interpretation is incorrect. Labels used in Yu et al. are SNP genotypes, which are readily available after genotyping. No labeler is involved so there is no bias.

We thank the reviewer for carefully examining the literature review section. Yes, the reviewer interpretation is absolutely correct. We have updated this section of the introduction.

Heritability. Please add some details in the Methods section on how the heritability is calculated.

We have added the heritability derived process with LDSC in the methods section. We now call it "LDSC-estimated SNP heritability".

I found it interesting that for the 10 loci have not been previously reported to associate with any of the top five traits related to brain structure or function. The most commonly reported non-brain-related traits include bone mineral density, height, body mass index (BMI), body fat distribution, and cardiovascular-related traits such as carpal tunnel syndrome, as well as some lung function and colorectal disease measures. The authors reasoned that these traits are more or less age-related making them reasonable. But I think potential rationale is that these traits are related to body or tissue morphology and development, which may also affect the shape and size of the brain.

We thank the reviewer for pointing out this possibility. Among the identified novel loci, 10 out of 24 appear to be broadly related to body or tissue morphology. Although these loci are not previously associated with brain-related traits, many map to genes involved in fundamental developmental pathways, such as Wnt and TGF-β signaling. These pathways regulate cell proliferation, differentiation, and tissue patterning across multiple organ systems. As a result, associations with traits such as bone mineral density, height, and body composition may reflect shared biological mechanisms governing overall morphology, including brain structure. For example: Locus 11 (lead SNP: rs34644194, chr2:50Mb, mapped gene: TCF7L1) is associated with heel bone mineral density and blood pressure. TCF7L1 regulates Wnt signaling, a pathway critical for early brain development and cortical expansion. Locus 56 (lead SNP: rs4444235, chr14:20Mb, mapped gene: BMP4) has been associated with heel bone mineral density, lung function (FEV1/FVC), and colorectal disease. BMP4 is a key morphogen in both skeletal and neural development, influencing cortical patterning and neural progenitor differentiation. These examples illustrate that variants influencing "non-brain" traits may also impact brain morphology, likely through shared developmental and tissue growth programs rather than brain-specific processes. Details of other loci can be found in the Supplementary Table 5. We have included the entire rationale in the updated manuscript (Line290-295).

For the GWAS results, the authors claims that 24 genetic loci out of 63 found to be associated with brain structure by the ViT approach were missed by a previous CNN-AE-based approach" and considered this a success. While I agree ViT is perhaps a more powerful method than CNN, but this claim is a bit misleading. I wonder, conversely, whether there is any locus that is identified by CNN and is missed by ViT. The two methods share some common findings as well as some unique findings, this alone doesn't make one method superior to the other. Unless the new method identified all the loci found before and additionally, it found 24 new ones and all the new loci are biologically meaningful. Some clarification is warranted on this point in my opinion.

We thank the reviewer for pointing this out. We observe that 39 of the 78 loci identified by the CNN are not recovered by the ViT. However, the primary goal of this work is not a direct numerical

comparison of loci counts, but rather to investigate what ViT can capture that CNN may miss, particularly in terms of its ability to model both local and non-local features in brain imaging. We do not claim that ViT is inherently superior to CNN; instead, our results suggest that the two architectures provide complementary information for loci discovery. We have added a paragraph in the discussion section to clarify this point (line411-413).

Reviewer#3:

The paper proposes using Visual Transformers to generate a new class of deep learning image-derived phenotypes (IDP), which the authors have termed 'ViT-UIDP'. These phenotypes are then studied using Genome-Wide Association Studies (GWAS) in the UK Biobank population cohort. Interesting original loci of association are derived and annotated. The ViT-UIDP phenotype class is then compared to another DL-derived IDP class that the authors of the paper previously developed (CNN-UIDP).

This contribution is well organised and follows a classic structure. The paper reports on the genetic association of the embeddings learnt by an unsupervised model equipped with attention modules on local/non-local features which is of clear interest for the brain mapping community.

The papers is proposed with companion code. I did not test the repository but I parsed it and checked that it follows the state of the art (requirements.txt, example entry, lean source entry - with classical organization using torch only as deep-learning module dependency).

Yet, I have a set of major issues to be addressed in the paper.

1. Introduction

The progression of ideas in this paragraph is unclear. It should be reorganized and expanded. I suggest organizing the ideas more clearly. 1) Starting from the existing text, the authors could begin with a general statement about brain structure and genetics (justify why you selected these citations – there are many more!), 2) The use hand-crafted phenotypes that were historically developed in small cohorts using image processing (cite classical features dedicated to cortex, grey matter, white matter and structural and functional connectivity), 3) The move to data-driven phenotype extraction using supervised and unsupervised methods. More references should be given beyond (Patel 2024), such as DOIs listed at thereafter. Introduce ViT as an alternative to CNN. 5) Introduce PerDI (citing Patel) and also the concept of spotlighting. Finally, introduce the main results annotation: yielding loci with annotations and yielding dimension with PerDI annotations.

I suggest to introduce the work on the specificity of the ViT architecture investigated thanks to SpotLight token which would justify the local and non-local words of the title.

I think it is important to introduce better all the points of the paper with really more citations for all the points of the introduction.

Proposed citations for embedding and genetics

https://doi.org/10.1371/journal.pgen.1011332
https://direct.mit.edu/imag/article/doi/10.1162/IMAG.a.987/133600

We thank the reviewer for the suggestion on the introduction paragraph. Yes, that is definitely a better flow for introducing our work, that is beginning by situating our work within the established link between brain structure and genetics, highlighting prior studies that rely on hand-crafted imaging phenotypes derived from small cohorts and classical image-processing approaches, followed by more recent shifts toward data-driven phenotype extraction using supervised and unsupervised learning methods, including CNNs and emerging alternatives such as Vision Transformers (ViTs). We have included the recommended citation from the reviewer and more to support our claim. And we have reworked the introduction as suggested (Line 34-63).

2. Methods

2.1 Data selection: I do not share the authors' opinion about the potential for information leakage if the same data were used to: i) train the ViT model, and ii) conduct the GWA study on the embeddings. Instead, I think the same subjects could have been considered, bearing in mind that genetics is never used in training the ViT model. It is a pity, since, as the authors note, a larger training set would improve the quality of the training and association findings (see the remarks in the Results section). I would like another argument to be given for this choice of split, as I believe it is misleading.

We thank the reviewer for raising this point. Indeed, if one can confidently claim that genetics information is never used in the training of the phenotyping model, it might be acceptable to reuse the training set for genetic association studies. We admit that our experiment setting is too conservative, but we prioritized maintaining a clean evaluation of model performance. We leave the solution to the data hungry issue of ViT to future model architectures like MAE. (Line 450-460)

2.2 Why so many covariables were chosen: are there specific reasons related to the choice of the architecture and training? Justification could again be given based on citations. Alternatively, the effect size and significance could be given for variables such as "date of attending", "scanner table position". On the contrary why only 10 genetic PC are kept and not the 40 ones available?

We selected GWAS covariates following prior Elliott et al. 2018 UK Biobank imaging GWAS study and Smith et al. 2021 UK Biobank imaging genetics study, taking the union of covariates used in both studies. In particular, these include "date of attending" and "scanner table position."

For genetic PCs, the UK Biobank recommends using a limited number of leading PCs to account for population structure, noting that later PCs tend to capture complex LD structure rather than ancestry-related variation (https://www.ukbiobank.ac.uk/publications/efficient-toolkit-implementing-best-practices-for-principal-component-analysis-of-population-genetic-data/). Based on this guidance, we include the top 10 PCs in our analysis.

2.3 minP is used to perform a conservative multivariate analysis. Could the procedure be detailed a little bit in the main manuscript: do you use permutation or simply take the min across all the features summary-statistics?

We take the minimum across all feature summary statistics and note that this provides a conservative lower bound compared to more advanced multivariate GWAS approaches (see Discussion).

2.4 The sparse attention pattern. It I unclear to me if the sparse attention pattern is a consequence of the choice by the authors of the \sigma_{r} version of the matrix \sigma (see the explanation given in Supplementary Note 3. If this is true, I think some information should be given in the main text in the Method part.

We thank the reviewer for the comment. To clarify, \Sigma_{r} contains the nonzero singular values of \Sigma. Since W_K^TW_Q of each attention head is rank-deficient, \Sigma has at least d – d_h zero singular values, where d is the embedding dimension (usually 8 * d_h or 16 * d_h). We use \Sigma_{r} instead of \Sigma because we want to emphasize that the alignment of embeddings happens on a lower-dimensional subspace defined by the dimension of the attention head. So the \Sigma_{r} is not manually chosen by us.

3. Results

3.1 Figure 3: A Miami plot could be given to relate/contrast the ViT and CNN UIDPs

We thank the author for the suggestion. We have included the Miami plot between ViT and CNN UDIPs in the Supplementary Figure 11.

3.2 In the paragraph starting with: "Out of the 24 loci…" and "In contrast, 10 loci….", The authors mention "brain structure or function" annotation. Could you give more details about the generation of

these annotations and their source? Is it an output from FUMA? As far as I know this is not direct output and human processing must be performed: could it be mentioned in the main text.

Yes, that is the output of the gwascatalog.txt directly downloaded from FUMA website. We describe this process in the Methods section "Querying GWAS catalog" (Line135-138), and we have updated the result session to directly use the word 'GWAS catalog' (Line273-296).

3.3 Regarding the 3 next paragraphs "Mutiple genes in …", "TCF7LI, a transcription factor…" and "Beyond neuronal and metabolic…". I think they are unduly detailed. They reflect the FUMA output which could be reported in the Supplemental data and referred. Finally, the conclusions of these (probably LLM generated) text is not supported by any data/results in the paper (they may be not false but they are not supported by reported elements). Could this part be truncated and left with only the very succinct report of what those gene are known to be linked with (experimentally verified). Also the sentence "Find table below" looks strange?

We thank the author for the suggestion. We have cut this paragraph and moved part of the results into the Supplementary Notes 4.

3.4 Spotlight investigation. Figure 5 is difficult for non-specialists to understand, especially the legend for part 5C. The bipartite graph should also be clarified with regard to the start and end points of the arrows. More generally, this section would be clearer if a counterpart paragraph were included in the 'Methods' section.

The paragraph "We found that this behavior arises from the specific embeddings learned by the model (see Supplementary Note 3). The …." should be clarified: the notion of "similar direction and weak correlation" should be really reworked.

The same with the paragraph "We then visualize the spatial probability of spotlight tokens across brain slices for models trained with different parameter settings and random seeds. As shown in Figure 5c, red regions highlight anatomical locations where the model consistently concentrates its attention across […] These results indicate strong robustness and stability of the identified spotlight tokens." The concepts of 'masked brain' patches and the discussion of 'high and low correlations' refer to contextual information that should be explained here. The experimental setup used to produce these numbers (18% 0.75 0.92..) should also be described in detail.

We thank the reviewer for bringing this up. We admit this part is hard to understand and are sorry for the confusion. We have reworked the Results section and Figure 5B to include a legend and explain it more clearly (Line372-375). We have also expanded the Methods section ("Sparse Attention Patterns Analysis and Visualization") to provide additional details on the experimental procedures. Hope that would solve the confusion on the figure5c result description.

3.5 Suppl figure 3. The legend for the colors appearing in the figure should be given.

We thank the reviewer for bringing this up. We have included the color map for Supplementary Figure 3.

“Vision Transformer Autoencoders for Unsupervised Representation Learning: Capturing Local and Non-Local Features in Brain Imaging to Reveal Genetic Associations” Response Letter

Editor:

We hope you will find the referees' comments useful as you decide how to proceed. While we would generally ask that you address all discussion points, methodological clarifications, and analytical concerns raised by the reviewers, it would be particularly important to:

(1) Evaluate the reproducibility of these UDIPs (per Reviewer #2)

We thank the reviewer for bringing up this comment. Individual UDIP dimensions will change when using different training datasets or even different random seeds. However, the overall multivariate distribution of UDIPs, as well as the loci discovered based on them, remains largely consistent. To demonstrate the reproducibility of these UDIPs, we trained the network under three sources of variability: different training seeds, different data splits, and distinct datasets (ADNI) with varying patient backgrounds. The results show strong alignment in UDIP distributions (as measured by CKA and CCA) and a high degree of overlap in the identified loci. Specifically, CKA (range 0 to 1) achieves an average of 0.83 across different training seeds, 0.80 across different data splits, and 0.60 across distinct datasets. CCA between UDIPs shows strong alignment, with correlations exceeding 0.8 for the first 30 canonical components, indicating that these components can be linearly transformed to closely match one another and are therefore effectively interchangeable. Moreover, the explained variance of one UDIP by another averages around 0.7, where explained variance is defined as the fraction of the total variance in X that is shared with Y. The overlap analysis shows an average of 37 loci for different training seeds, 35 for different data splits, and 17 for distinct datasets. These findings have been added to Methods (Line112-117), Results (Line 237-245), Discussion (Line 417-426), Supplementary Note 5, and Supplementary Figure 13-15.

(2) Justify the broad selection of covariates (per Reviewers #1 and #3), including Reviewer #1’s concern about the reliance on head size

We thank the reviewer for this comment. We follow the same list of covariates from Patel et al 2024. To be more specific, this list includes GWAS covariates following the prior Elliott et al. 2018 UK Biobank imaging GWAS study and Smith et al. 2021 UK Biobank imaging genetics study, taking the union of covariates used in both studies. As for the head size covariate, many existing well-known papers include head size as the confounding covariate for the GWAS analysis, including Elliott et al., 2018, The MULTI Consortium, Boquet-Pujadas et al, 2025. To further answer the reviewer’s question, we conduct the GWAS analysis without the head size covariate. The resulting loci and leadSNP remain largely unchanged (Supplementary Figure 16).

(3) Clarify how novel variants were determined (Reviewer #1) and whether any of these are more broadly relevant to body or tissue morphology (Reviewer #2)

We thank the reviewer for this valuable suggestion. We have updated the Methods section to include a session of “define novel loci”. Among the identified novel loci, 10 out of 24 appear to be broadly related to body or tissue morphology. Although these loci are not previously associated with brain-related traits, many map to genes involved in fundamental developmental pathways, such as Wnt and TGF-β signaling. These pathways regulate cell proliferation, differentiation, and tissue patterning across multiple organ systems. As a result, associations with traits such as bone mineral density, height, and body composition may reflect shared biological mechanisms governing overall morphology, including brain structure. For example: Locus 11 (lead SNP: rs34644194, chr2:50Mb, mapped gene: TCF7L1) is associated with heel bone mineral density and blood pressure. TCF7L1 regulates Wnt signaling, a pathway critical for early brain development and cortical expansion.

Locus 56 (lead SNP: rs4444235, chr14:20Mb, mapped gene: BMP4) has been associated with heel bone mineral density, lung function (FEV1/FVC), and colorectal disease. BMP4 is a key morphogen in both skeletal and neural development, influencing cortical patterning and neural progenitor

differentiation. These examples illustrate that variants influencing “non-brain” traits may also impact brain morphology, likely through shared developmental and tissue growth programs rather than brain-specific processes. Details of other loci could be found in the Supplementary Table 5. We have included the entire rationale in the updated manuscript (Line291-296).

(4) Justify the data selection (Reviewer #3; point 2.1). While we recognize that paired neuroimaging and genetic data are sparse, we would strongly encourage you to include additional validation in other cohorts where possible, or at the very least better acknowledge the limitations of this approach and the reliance on solely UKBB data.

We thank the reviewer for raising this point. We acknowledge the limitation of this approach for reliance on the UKBB data and for Reviewer #3; point 2.1. We have included more balanced discussion of limitations of the cohort design and the need for further validation (Line481-496). We also admit our approach might be over-conservative for strictly separate GWAS and training cohorts, but we prioritized maintaining a clean and reliable evaluation of model performance. We include this in the Discussion (Line450-452).

Reviewer#1:

The authors explored the potential of using unsupervised learning derived image phenotypes for discovering genetic associations, in UK Biobank structural brain image and genetic data. This is a timely endeavor and an interesting approach, which has the potential to overcome limitations of traditional approaches based on e.g. atlas-defined brain regions.

I do not have expertise in unsupervised learning applied to neuroimage data, but I can comment from the perspective of genetic analysis applied to brain imaging.

From the introduction: ‘Previous works on brain imaging GWAS have primarily focused on image-derived phenotypes (IDPs) via image processing pipelines (Elliott et al., 2018; Smith et al., 2021). However, IDPs can miss subtle or complex patterns due to their reliance on predefined features and model assumptions ...’. I think this description does not capture the extent to which data-driven IDPs have been used in genome-wide association studied (GWAS), such as derived from independent component analysis of resting functional data. If the authors could explicitly rephrase this part around brain structure GWAS, rather than brain imaging GWAS more generally, then it would be more accurate.

We thank the reviewer for bringing this to our attention. We have rephrased accordingly.

In the author’s GWAS, head size was used as a confound covariate. Head size is a heritable measure, and using heritable measures as covariates in GWAS can cause bias (Aschard et al. 2015 https://www.sciencedirect.com/science/article/pii/S0002929714005278?via=ihub), including spurious associations driven by the covariate adjustment. It would be advisable to run GWAS also without head size adjustment and compare the results.

We thank the reviewer for this comment. We follow the same list of covariates from Patel et al 2024. To be more specific, this list includes GWAS covariates following the prior Elliott et al. 2018 UK Biobank imaging GWAS study and Smith et al. 2021 UK Biobank imaging genetics study, taking the union of covariates used in both studies. As for the head size covariate, many existing well-known papers include head size as the confounding covariate for the GWAS analysis, including Elliott et al., 2018, The MULTI Consortium, Boquet-Pujadas et al, 2025. To further answer the reviewer’s question, we conduct the GWAS analysis without the head size covariate. The resulting loci and leadSNP remain largely unchanged (Supplementary Figure 16).

I found some aspects of the sample splitting to be unclear. From the Methods: ‘6,130 MRI scans were chosen for the model and split into 75/25 training/validation sets. 4,597 scans were used for training, and 1,533 scans were used for validation. The dataset used for GWAS consisted of 37,376 scans, of which we selected 22,867 as discovery cohorts for later GWAS analysis.’ Then in the

Discussion 'As an experimental approach, we trained our ViT-AE model using the combined original training and testing datasets and validated it on a disjoint validation set ... Although the trained model identified additional associated loci, these findings were excluded from the main results because the GWAS analysis was conducted on the original testing dataset, which was included in the training process ... Nonetheless, we present these findings to illustrate the ViT-AE model's potential to perform better with a larger training dataset.' These aspects are difficult for the reader to follow, so please clarify with exact details on dividing the sample for which purposes and explaining the rationale in the Methods section, so that the reader can understand which results may have been affected by exaggerated model performance. A schematic figure might help.

We thank the reviewer for bringing this up. The original data split was designed to match the "discovery cohort" setup used in Elliott et al. 2018 UK Biobank imaging GWAS study and Smith et al. 2021 UK Biobank imaging genetics study, where a large portion of the UK Biobank samples is reserved for GWAS, leaving only a few thousand samples for model training. However, ViTs, as transformer-based architectures, are known to be data-hungry. We therefore expect that increasing the training set size would improve performance. Motivated by this, we conducted an additional experiment in which the "test data" (replication cohort), originally reserved solely for GWAS, is also incorporated into the training set.

We have modified the discussion to make it clearer (Line444-452).

For overlap analysis, the authors cross-referenced their GWAS hits that had Bonferroni-corrected significance p< 5×10-8/128 against previously reported GWAS hits from Smith et al. 2021 that had Bonferroni-corrected significance p< 5×10-8/1437. The Bonferroni correction was more severe for Smith's findings, because Smith et al., ran GWAS with a greater number of brain structural measures. However, a consequence is that the GWAS hits from Smith et al. would need to be roughly one order of magnitude more significant than those in the present study, before Bonferroni adjustment. Then, the Results text (and e.g. the Fig 3 legend, the Table 1 contents, and the abstract) focuses on comparing GWAS results from the present study with an earlier study by Patel et al. (2024) who used a convolutional neural network (CNN) approach for deriving brain structural measures for GWAS. The reported numbers of novel genetic loci in the present study are made with reference to Patel et al., rather than Smith et al.. This leaves the question of how many of the genetic loci were truly novel, and this appears to be answered at the start of the discussion, with reference to the GWAS catalog. I understand that the overlap with Smith et al. was used as a metric to asses model performance, but this creates a confusing aspect to the paper where the description of the Results does not follow clearly from the overlap analysis in the Methods. This leaves readers wondering how many of the genetic loci were truly novel in the present study. Please clarify throughout the paper what is meant by 'new' or 'novel' loci, with respect to Smith et al., Patel et al. and GWAS catalog.

We thank the reviewer for this valuable suggestion. We report loci overlap of ViT-UDIPs with both Smith et al. 2021 UK Biobank imaging genetics study and Patel et al. imaging genetics study in the newly added Supplementary Figure 12 and Supplementary Table 6. Twenty loci from ViT-UDIPs remain novel after loci overlapping analysis with both CNN-UDIPs and IDPs. We have updated the Methods section to include a session of "define novel loci", as well as clarified the meaning of 'new' or 'novel' loci within the Results section, such that the reader should not get confused (Line 255-257).

For the GWAS Catalog comparison, which aggregates loci reported across a large body of literature, we provide a qualitative assessment by highlighting the studies with the five most significant (lowest p-value) associations (Line 277-297)

From the Results: 'Out of the 24 loci newly identified by our method, 14 loci show at least one of their top five associated traits related to brain structure or function.' Does this refer to the GWAS catalog? Please clarify.

We are sorry for not making the statement clear. Yes, this refers to the GWAS catalog. We have modified the related Results session to directly use the word 'GWAS catalog' (Line273-297).

From the Results: 'In contrast, 10 loci have not been previously reported to associate with any of the top five traits related to brain structure or function.' Does this mean that none of the top 5 traits in the GWAS catalog for any of these 10 loci was related to brain structure or function? Please clarify.

Yes, the reviewer understands it correctly. We have clarified it in the Line 287-288.

In the Results and Discussion, the authors claim that bone mineral density could indirectly influence gray matter changes in the brain. Please also acknowledge the possibility of horizontal pleiotropy, when a single genetic variant independently influences distinct traits, rather than affecting one trait through another.

We thank the reviewer for bringing this up. We have updated both parts to include this possibility (Line 351-352, Line 503-505).

In the Discussion, the authors report that their model gives a disproportionate amount of attention to 'anatomically informative regions with high structural variance, such as the boundaries between different tissue types (e.g., gray and white matter), the edges of ventricles, or the complex folding of deep sulci.' This seems to be both a potential strength and also weakness of the approach. A strength because it confirms that the model is sensitive to the most overt features of the image data, so is working broadly as expected. A weakness because more subtle aspects of brain structure are overlooked. The Discussion could be more nuanced on this point.

We thank the reviewer for raising this point. The subtle aspects refer to the other regions of the brain that do not receive prominent attention weights. We want to emphasize that attention patterns are learned, and are more than just the most prominent ones that we highlighted. There are more subtle changes - these are also captured by the model's attention mechanisms - but are less interpretable, and thus not highlighted. We have included a nuanced interpretation in the discussion section (Line 437-440).

In Table 1 and supplementary Table 4 please include information on minor allele frequency and association effect size.

We have included the minor allele frequency (MAF) and association effect size (BETA) for the leadSNP within loci in the Table 1 and Supplementary Table 4.

I find a section heading from the Results difficult to follow: 'Novel loci lead SNPs gene discovered associated with brain structure'.

We have changed the section heading to be 'Novel loci discovered associated with brain structure'.

Reviewer#2:

In this paper, Islam et al. described a framework that applies a Vision Transformer (ViT)-based autoencoder to derive 128-dimensional representations from brain MRI scans of UK Biobank participants. These vectors are named unsupervised learning derived image phenotypes from ViT (ViT-UDIP). Then Islam et al. conducted GWAS on these UDIP using UK Biobank data and identified 63 loci. Their findings demonstrate the value of transformer-based architectures in discovering novel and robust imaging phenotypes for genetic discovery.

The UDIP idea is very interesting and an attractive alternative to IDP. Overall, the paper is well written and is of high quality.

But an unsettling issue for me is whether these UDIPs are reproducible. I mean, if one repeats the same procedure on a different training dataset, whether the exact same set of UDIP will be derived and in the same order? I doubt this since the ViT or NN are complex models and dynamic. Therefore, how to match these UDIPs could be a challenging problem.

We thank the reviewer for pointing this out. Individual UDIP dimensions will change when using different training datasets or even different random seeds. However, the overall multivariate distribution of UDIPs, as well as the loci discovered based on them, remains largely consistent. To support this, we performed CKA, CCA analyses, and loci-overlap experiments across UDIPs derived from different training seeds and training datasets (See Supplementary Note 5 and Supplementary Figure 13-15).

In the introduction section, some of the literature reviews are inaccurate. For example, the authors wrote that "Yu et al. demonstrated… The primary challenge with this approach is that labor-intensive labeled data for supervised deep learning carry the same biases from the human labeler." This interpretation is incorrect. Labels used in Yu et al. are SNP genotypes, which are readily available after genotyping. No labeler is involved so there is no bias.

We thank the reviewer for carefully examining the literature review section. Yes, the reviewer interpretation is absolutely correct. We have updated this section of the introduction.

Heritability. Please add some details in the Methods section on how the heritability is calculated.

We have added the heritability derived process with LDSC in the methods section. We now call it "LDSC-estimated SNP heritability".

I found it interesting that for the 10 loci have not been previously reported to associate with any of the top five traits related to brain structure or function. The most commonly reported non-brain-related traits include bone mineral density, height, body mass index (BMI), body fat distribution, and cardiovascular-related traits such as carpal tunnel syndrome, as well as some lung function and colorectal disease measures. The authors reasoned that these traits are more or less age-related making them reasonable. But I think potential rationale is that these traits are related to body or tissue morphology and development, which may also affect the shape and size of the brain.

We thank the reviewer for pointing out this possibility. Among the identified novel loci, 10 out of 24 appear to be broadly related to body or tissue morphology. Although these loci are not previously associated with brain-related traits, many map to genes involved in fundamental developmental pathways, such as Wnt and TGF-β signaling. These pathways regulate cell proliferation, differentiation, and tissue patterning across multiple organ systems. As a result, associations with traits such as bone mineral density, height, and body composition may reflect shared biological mechanisms governing overall morphology, including brain structure. For example: Locus 11 (lead SNP: rs34644194, chr2:50Mb, mapped gene: TCF7L1) is associated with heel bone mineral density and blood pressure. TCF7L1 regulates Wnt signaling, a pathway critical for early brain development and cortical expansion. Locus 56 (lead SNP: rs4444235, chr14:20Mb, mapped gene: BMP4) has been associated with heel bone mineral density, lung function (FEV1/FVC), and colorectal disease. BMP4 is a key morphogen in both skeletal and neural development, influencing cortical patterning and neural progenitor differentiation. These examples illustrate that variants influencing "non-brain" traits may also impact brain morphology, likely through shared developmental and tissue growth programs rather than brain-specific processes. Details of other loci can be found in the Supplementary Table 5. We have included the entire rationale in the updated manuscript (Line291-296).

For the GWAS results, the authors claims that 24 genetic loci out of 63 found to be associated with brain structure by the ViT approach were missed by a previous CNN-AE-based approach" and considered this a success. While I agree ViT is perhaps a more powerful method than CNN, but this claim is a bit misleading. I wonder, conversely, whether there is any locus that is identified by CNN and is missed by ViT. The two methods share some common findings as well as some unique findings, this alone doesn't make one method superior to the other. Unless the new method identified all the loci found before and additionally, it found 24 new ones and all the new loci are biologically meaningful. Some clarification is warranted on this point in my opinion.

We thank the reviewer for pointing this out. We observe that 39 of the 78 loci identified by the CNN are not recovered by the ViT. However, the primary goal of this work is not a direct numerical comparison of loci counts, but rather to investigate what ViT can capture that CNN may miss, particularly in terms of its ability to model both local and non-local features in brain imaging. We do not claim that ViT is inherently superior to CNN; instead, our results suggest that the two architectures provide complementary information for loci discovery. We have added a paragraph in the discussion section to clarify this point (line412-414).

Reviewer#3:

The paper proposes using Visual Transformers to generate a new class of deep learning image-derived phenotypes (IDP), which the authors have termed 'ViT-UIDP'. These phenotypes are then studied using Genome-Wide Association Studies (GWAS) in the UK Biobank population cohort. Interesting original loci of association are derived and annotated. The ViT-UIDP phenotype class is then compared to another DL-derived IDP class that the authors of the paper previously developed (CNN-UIDP).

This contribution is well organised and follows a classic structure. The paper reports on the genetic association of the embeddings learnt by an unsupervised model equipped with attention modules on local/non-local features which is of clear interest for the brain mapping community.

The papers is proposed with companion code. I did not test the repository but I parsed it and checked that it follows the state of the art (requirements.txt, example entry, lean source entry - with classical organization using torch only as deep-learning module dependency).

Yet, I have a set of major issues to be addressed in the paper.

1. Introduction

The progression of ideas in this paragraph is unclear. It should be reorganized and expanded. I suggest organizing the ideas more clearly. 1) Starting from the existing text, the authors could begin with a general statement about brain structure and genetics (justify why you selected these citations – there are many more!), 2) The use hand-crafted phenotypes that were historically developed in small cohorts using image processing (cite classical features dedicated to cortex, grey matter, white matter and structural and functional connectivity), 3) The move to data-driven phenotype extraction using supervised and unsupervised methods. More references should be given beyond (Patel 2024), such as DOIs listed at thereafter. Introduce ViT as an alternative to CNN. 5) Introduce PerDI (citing Patel) and also the concept of spotlighting. Finally, introduce the main results annotation: yielding loci with annotations and yielding dimension with PerDI annotations.

I suggest to introduce the work on the specificity of the ViT architecture investigated thanks to SpotLight token which would justify the local and non-local words of the title.

I think it is important to introduce better all the points of the paper with really more citations for all the points of the introduction.

Proposed citations for embedding and genetics

https://doi.org/10.1371/journal.pgen.1011332
https://direct.mit.edu/imag/article/doi/10.1162/IMAG.a.987/133600

We thank the reviewer for the suggestion on the introduction paragraph. Yes, that is definitely a better flow for introducing our work, that is beginning by situating our work within the established link between brain structure and genetics, highlighting prior studies that rely on hand-crafted imaging phenotypes derived from small cohorts and classical image-processing approaches, followed by more recent shifts toward data-driven phenotype extraction using supervised and unsupervised learning methods, including CNNs and emerging alternatives such as Vision Transformers (ViTs).

We have included the recommended citation from the reviewer and more to support our claim. And we have reworked the introduction as suggested (Line 34-63).

2. Methods

2.1 Data selection: I do not share the authors' opinion about the potential for information leakage if the same data were used to: i) train the ViT model, and ii) conduct the GWA study on the embeddings. Instead, I think the same subjects could have been considered, bearing in mind that genetics is never used in training the ViT model. It is a pity, since, as the authors note, a larger training set would improve the quality of the training and association findings (see the remarks in the Results section). I would like another argument to be given for this choice of split, as I believe it is misleading.

We thank the reviewer for raising this point. Indeed, if one can confidently claim that genetics information is never used in the training of the phenotyping model, it might be acceptable to reuse the training set for genetic association studies. We admit that our experiment setting is too conservative, but we prioritized maintaining a clean evaluation of model performance. We leave the solution to the data hungry issue of ViT to future model architectures like MAE. (Line 451-461)

2.2 Why so many covariables were chosen: are there specific reasons related to the choice of the architecture and training? Justification could again be given based on citations. Alternatively, the effect size and significance could be given for variables such as “date of attending”, “scanner table position”. On the contrary why only 10 genetic PC are kept and not the 40 ones available?

We selected GWAS covariates following prior Elliott et al. 2018 UK Biobank imaging GWAS study and Smith et al. 2021 UK Biobank imaging genetics study, taking the union of covariates used in both studies. In particular, these include “date of attending” and “scanner table position.”

For genetic PCs, the UK Biobank recommends using a limited number of leading PCs to account for population structure, noting that later PCs tend to capture complex LD structure rather than ancestry-related variation (https://www.ukbiobank.ac.uk/publications/efficient-toolkit-implementing-best-practices-for-principal-component-analysis-of-population-genetic-data/). Based on this guidance, we include the top 10 PCs in our analysis.

2.3 minP is used to perform a conservative multivariate analysis. Could the procedure be detailed a little bit in the main manuscript: do you use permutation or simply take the min across all the features summary-statistics?

We take the minimum across all feature summary statistics and note that this provides a conservative lower bound compared to more advanced multivariate GWAS approaches (see Discussion).

2.4 The sparse attention pattern. It I unclear to me if the sparse attention pattern is a consequence of the choice by the authors of the \sigma_{r} version of the matrix \sigma (see the explanation given in Supplementary Note 3. If this is true, I think some information should be given in the main text in the Method part.

We thank the reviewer for the comment. To clarify, \Sigma_{r} contains the nonzero singular values of \Sigma. Since W_K^TW_Q of each attention head is rank-deficient, \Sigma has at least d – d_h zero singular values, where d is the embedding dimension (usually 8 * d_h or 16 * d_h). We use \Sigma_{r} instead of \Sigma because we want to emphasize that the alignment of embeddings happens on a lower-dimensional subspace defined by the dimension of the attention head. So the \Sigma_{r} is not manually chosen by us.

3. Results

3.1 Figure 3: A Miami plot could be given to relate/contrast the ViT and CNN UIDPs

We thank the author for the suggestion. We have included the Miami plot between ViT and CNN UDIPs in the Supplementary Figure 11.

3.2 In the paragraph starting with: “Out of the 24 loci…” and “In contrast, 10 loci….”, The authors mention “brain structure or function” annotation. Could you give more details about the generation of these annotations and their source? Is it an output from FUMA? As far as I know this is not direct output and human processing must be performed: could it be mentioned in the main text.

Yes, that is the output of the gwascatalog.txt directly downloaded from FUMA website. We describe this process in the Methods section “Querying GWAS catalog” (136-139), and we have updated the result session to directly use the word ‘GWAS catalog’ (Line274-297).

3.3 Regarding the 3 next paragraphs “Mutiple genes in …”, “TCF7LI, a transcription factor…” and “Beyond neuronal and metabolic…”. I think they are unduly detailed. They reflect the FUMA output which could be reported in the Supplemental data and referred. Finally, the conclusions of these (probably LLM generated) text is not supported by any data/results in the paper (they may be not false but they are not supported by reported elements). Could this part be truncated and left with only the very succinct report of what those gene are known to be linked with (experimentally verified). Also the sentence “Find table below” looks strange?

We thank the author for the suggestion. We have cut this paragraph and moved part of the results into the Supplementary Notes 4.

3.4 Spotlight investigation. Figure 5 is difficult for non-specialists to understand, especially the legend for part 5C. The bipartite graph should also be clarified with regard to the start and end points of the arrows. More generally, this section would be clearer if a counterpart paragraph were included in the 'Methods' section.

The paragraph “We found that this behavior arises from the specific embeddings learned by the model (see Supplementary Note 3). The ….” should be clarified: the notion of “similar direction and weak correlation” should be really reworked.

The same with the paragraph “We then visualize the spatial probability of spotlight tokens across brain slices for models trained with different parameter settings and random seeds. As shown in Figure 5c, red regions highlight anatomical locations where the model consistently concentrates its attention across […] These results indicate strong robustness and stability of the identified spotlight tokens.” The concepts of 'masked brain' patches and the discussion of 'high and low correlations' refer to contextual information that should be explained here. The experimental setup used to produce these numbers (18% 0.75 0.92..) should also be described in detail.

We thank the reviewer for bringing this up. We admit this part is hard to understand and are sorry for the confusion. We have reworked the Results section and Figure 5B to include a legend and explain it more clearly (Line373-376). We have also expanded the Methods section (“Sparse Attention Patterns Analysis and Visualization”) to provide additional details on the experimental procedures. Hope that would solve the confusion on the figure5c result description.

3.5 Suppl figure 3. The legend for the colors appearing in the figure should be given.

We thank the reviewer for bringing this up. We have included the color map for Supplementary Figure 3.

“Vision Transformer Autoencoders for Unsupervised Representation Learning: Capturing Local and Non-Local Features in Brain Imaging to Reveal Genetic Associations” Response Letter

Reviewer #1 (Remarks to the Author):

Thank you for addressing my earlier points with additional information and clarification.

I am still somewhat confused by the dataset splitting.
From the ‘Methods’: ‘The dataset used for GWAS consisted of 37,376 scans, of which we selected 22,867 as discovery cohorts for later GWAS analysis.’ Why is ‘cohorts’ here plural? Why not use the whole 37,376 for GWAS? Were the remaining 14,509 (i.e. 37,376-22,867) used for anything in this study? Or did the 14,509 not meet quality control in the genetic pipeline? On what basis were the 22,867 ‘selected’? Figure 1 mentions 37,376 as the testing set.
We thank the reviewer for raising this point and for the careful attention to detail. We reused the same discovery and replication cohorts that were previously established and published by our lab (Patel et al., 2024). The concept of separating the dataset into discovery and replication cohorts follows the framework introduced in the BIG40 paper from Smith et al, 2021 Nature Neuroscience (Smith et al., 2021). In that work, the authors used the following criteria to derive the **discovery and replication cohort**:

- “We consider the 488,377 samples included in the Spring 2018 release of UKB and proceed with a pre-processing and discovery/reproduction paradigm similar to that described in ref. 1. Of the samples, 39,944 are included among the 41,016 samples in UKB for which IDPs are available, after the genotyping quality control procedures for sample removal specified in ref. 53. We removed samples without recent UK ancestry as determined by the in.white.British.ancestry.subset variable in the file ukb_sqc_v2.txt provided in the meta-data for UKB. This variable selects samples based on self-reported ancestry and genetic principal component thresholds. We also removed individuals based on relatedness, forming a maximally unrelated subset using the procedures recommended in ref. 53. This resulted in a maximally unrelated subset of 34,298 samples with recent UK ancestry and accepted genotyping and imaging quality control. We divided this set into a discovery cohort and a reproduction cohort according to a random 2/3 and 1/3 (respective) proportion, resulting in a discovery cohort of 22,865 samples and a reproduction cohort of 11,433 samples.”

We applied the same standard sample filtering criteria, including genotype and imaging quality control, self-reported White British ancestry (UK Biobank field IDs 21000 and 22006), unrelatedness, and an additional requirement of concordance between reported and genetically inferred sex, along with exclusion of individuals on the withdrawal list as of 08/21/2023. We further restricted analyses to each participant’s first available visit and randomly partitioned the cohort into approximately 2/3 discovery and 1/3 replication sets, matching the discovery cohort size used in BIG40. The final GWAS dataset comprised 37,376 samples corresponding to 35,226 unique individuals (reflecting repeated visits). The discovery cohort included 23,976 samples (22,867 unique individuals), and the replication cohort included 13,400 samples (12,359 unique individuals).

For the remaining replication cohort samples we intend to save until more samples are available with the upcoming UKB data release, to match the same discovery cohort size, as the GWAS performance highly depends on the sample number.

As a modification, we have corrected the typo of cohorts to make it singular as well as added GWAS data selection and split criteria to derive discovery and replication cohort in the Methods section as follows: For the GWAS analysis samples, we reused the discovery and replication cohort previously established in the lab (Patel et al., 2024), following the framework of the BIG40 study (Smith et al., 2021). From UKB participants with both T1-weighted MRI and genomic data available, we applied standard quality control filters for genotype and imaging, White British ancestry (UK Biobank field

IDs 21000 and 22006), unrelatedness, concordant reported and genetic sex, and exclusion of withdrawn participants as of 08/21/2023. Analyses were restricted to the first visit, and the cohort was randomly split into ~2/3 discovery and 1/3 replication, matching the discovery cohort size used in BIG40. The final GWAS dataset included 37,376 samples (35,226 unique individuals; reflecting repeating visits), with 23,976 samples (22,867 individuals) in the discovery cohort and 13,400 samples (12,359 individuals) in the replication cohort.

I think that these two revised sentences in the introduction need to be corrected:
'Early genome-wide association studies (GWAS) have focused on direct phenotype measurements (Brouwer et al., 2022; Garcia-Marin et al., 2024; Hibar et al., 2015, 2017; Stein et al., 2012) or statistical and mathematical representations of anatomical modeling and population genetics (Brun et al., 2009; Hulshoff Pol et al., 2006). Recent works on brain structure GWAS have shifted to image-derived phenotypes (IDPs), driven by the availability of large-scale imaging datasets and processing pipelines. (Elliott et al., 2018; Smith et al., 2021; Zhao et al., 2019).'
- In the first of these sentences, referring to studies from 2022 (Brouwer et al.) and 2024 (Garcia-Marin et al.) as 'early' genome-wide association studies does not make much sense, as they were only published 2-4 years ago.
- In the second of these sentences, referring to how recent studies have 'shifted' to IDPs does not make much sense either. The 'recent' studies cited here are older than two of the 'early' studies in the sentence before. More importantly, many of the 'early' studies cited in the first sentence used IDPs as well. Maybe they did not call their brain measures IDPs, but they were typically from automated processing pipelines in large datasets. Therefore, the distinction made by the authors between 'direct phenotype measurements' in older studies and 'IDPs' in newer studies is not really valid. I think it should be possible for the authors to describe the novelty of their approach while still representing the previous literature correctly.
We thank the reviewer for pointing out these chronological inconsistencies and the flawed distinction between direct phenotype measurements and IDPs. We agree that referring to studies from 2022 and 2024 as 'early' while contrasting them with 'recent' studies from 2018 was confusing. Furthermore, the reviewer is correct that many previous large-scale consortia studies also utilized automated processing pipelines to generate what are fundamentally IDPs.

The distinction we draw is not intended as a strict chronological separation but rather reflects differences in the complexity and dimensionality of imaging-derived phenotypes (IDPs). One line of the work in imaging genetics primarily focused on a limited number of low-dimensional, anatomically interpretable phenotypes, such as regional brain volumes and cortical thickness measures derived from standard structural MRI analyses. A parallel line has shifted towards the extraction of hundreds to thousands of IDPs across multiple imaging modalities. In particular, centralized pipelines and large biobanks such as the UK Biobank have facilitated systematic GWAS of high-dimensional, multimodal phenotypic representations. Accordingly, we revise the introduction to frame the literature in terms of an evolution from targeted, macroscale structural metrics toward large-scale, fine-grained, and increasingly comprehensive IDP sets. We believe this framing more accurately reflects the existing literature while naturally motivating the novelty of our proposed approach.

The revised introduction is as follows: One line of work uses direct morphometric measurements or low-dimensional curated features (Brouwer et al., 2022; Garcia-Marin et al., 2024; Hibar et al., 2015, 2017; Stein et al., 2012) or statistical and mathematical representations of anatomical modeling and population genetics (Brun et al., 2009; Hulshoff Pol et al., 2006). A parallel line has shifted toward high-throughput image-derived phenotypes (IDPs) enabled by large-scale imaging cohorts and standardized processing pipelines. These IDPs features higher-dimensional or data-driven representations of brain structure (Elliott et al., 2018; Smith et al., 2021; Zhao et al., 2019).

Reviewer #2 (Remarks to the Author):

The authors have addressed most of my concerns. I noticed that the authors removed the mention of Yu et al. I am a bit disappointed that the authors seem unwilling to follow my review advice. I think this paper should be cited and discussed since the topic of that paper is closely related to the present study.
We thank the reviewer for pointing this out. We agree that the work by Yu et al. is closely related to the present study and should be acknowledged. We have now reinstated the citation and included this work in the Introduction to better contextualize our study within the existing literature. We appreciate the reviewer's suggestion, which helped improve the manuscript.

The added introduction text is as follows: Another interesting study is by Yu et al., who used SNPs as a supervision signal to train CNN models on 2D MRI images, ultimately selecting the top SNPs based on their prediction performance (Yu et al., 2024). This is essentially a novel approach to conducting non-linear GWAS using a deep learning architecture, with the sacrifice of interpretablity and the cost of train thousands of CNN models.

Reviewer #3 (Remarks to the Author):

The authors answered my questions adequately and rephrased their text when requested. In my opinion, they did so correctly with regard to the Editor's and other reviewers'.

I have two remarks, for which I apologise as I did not raise them in my first review. I am confident that the authors will respond without my intervention.
-The selection of 22,867 out of 37,776 subjects is neither justified nor explained anywhere. Could this be explained?
-Regular practice when performing a GWAS is to consider a sample from a homogeneous population (even if population structure is controlled with the ten PCs in the model). Did the authors only retain subjects with British ancestry (field 21000)? If so, this could be mentioned.

We thank reviewer for the good words.

We reused the same discovery and replication cohorts that were previously established and published by our lab (Patel et al., 2024). The concept of separating the dataset into discovery and replication cohorts follows the framework introduced in the BIG40 paper from Smith et al, 2021 Nature Neuroscience (Smith et al., 2021). In that work, the authors used the following criteria to derive the **discovery and replication cohort**:

- "We consider the 488,377 samples included in the Spring 2018 release of UKB and proceed with a pre-processing and discovery/reproduction paradigm similar to that described in ref. 1. Of the samples, 39,944 are included among the 41,016 samples in UKB for which IDPs are available, after the genotyping quality control procedures for sample removal specified in ref. 53. We removed samples without recent UK ancestry as determined by the in.white.British.ancestry.subset variable in the file ukb_sqc_v2.txt provided in the meta-data for UKB. This variable selects samples based on self-reported ancestry and genetic principal component thresholds. We also removed individuals based on relatedness, forming a maximally unrelated subset using the procedures recommended in ref. 53. This resulted in a maximally unrelated subset of 34,298 samples with recent UK ancestry and accepted genotyping and imaging quality control. We divided this set into a discovery cohort and a reproduction cohort according to a random 2/3 and 1/3 (respective) proportion, resulting in a discovery cohort of 22,865 samples and a reproduction cohort of 11,433 samples."

We applied the same standard sample filtering criteria, including genotype and imaging quality control, self-reported White British ancestry (UK Biobank field IDs 21000 and 22006), unrelatedness, and an additional requirement of concordance between reported and genetically inferred sex, along with exclusion of individuals on the withdrawal list as of 08/21/2023. We further restricted analyses to each participant's first available visit and randomly partitioned the cohort into approximately 2/3

discovery and 1/3 replication sets, matching the discovery cohort size used in BIG40. The final GWAS dataset comprised 37,376 samples corresponding to 35,226 unique individuals (reflecting repeated visits). The discovery cohort included 23,976 samples (22,867 unique individuals), and the replication cohort included 13,400 samples (12,359 unique individuals).

As a modification, we have added the GWAS data selection and split criteria to derive discovery and replication cohort in the Methods section as follows: For the GWAS analysis samples, we reused the discovery and replication cohort previously established in the lab (Patel et al., 2024), following the framework of the BIG40 study (Smith et al., 2021). From UKB participants with both T1-weighted MRI and genomic data available, we applied standard quality control filters for genotype and imaging, White British ancestry (UK Biobank field IDs 21000 and 22006), unrelatedness, concordant reported and genetic sex, and exclusion of withdrawn participants as of 08/21/2023. Analyses were restricted to the first visit, and the cohort was randomly split into ~2/3 discovery and 1/3 replication, matching the discovery cohort size used in BIG40. The final GWAS dataset included 37,376 samples (35,226 unique individuals; reflecting repeating visits), with 23,976 samples (22,867 individuals) in the discovery cohort and 13,400 samples (12,359 individuals) in the replication cohort.

For the second question, as mentioned above, yes, the GWAS cohort is specifically seleted with white British ancestry (field 21000 and 22006).

References:

Patel, K., Xie, Z., Yuan, H., Islam, S. M. S., Xie, Y., He, W., . . . Zhi, D. (2024). Unsupervised deep representation learning enables phenotype discovery for genetic association studies of brain imaging. Commun Biol, 7(1), 414. doi:10.1038/s42003-024-06096-7

Smith, S. M., Douaud, G., Chen, W., Hanayik, T., Alfaro-Almagro, F., Sharp, K., & Elliott, L. T. (2021). An expanded set of genome-wide association studies of brain imaging phenotypes in UK Biobank. Nat Neurosci, 24(5), 737-745. doi:10.1038/s41593-021-00826-4
